# Guardian ubiquitin E3 ligases target cancer-associated APOBEC3 deaminases for degradation to promote human genome integrity

**DOI:** 10.1038/s41467-026-68420-5

**Published:** 2026-01-19

**Authors:** Irene Schwartz, Valentina Budroni, Mathilde Meyenberg, Zuzana Hodakova, Harald Hornegger, Kathrin Hacker, Siegfried Schwartz, Daniel B. Grabarczyk, Julian F. Ehrmann, Sara Scinicariello, David Haselbach, Jörg Menche, Tim Clausen, G. Elif Karagöz, Gijs A. Versteeg

**Affiliations:** 1 Max Perutz Labs, Vienna BioCenter Campus (VBC), Dr. -Bohrgasse 9, Vienna, Austria; 2 University of Vienna, Center for Molecular Biology, Dr. -Bohrgasse 9, Vienna, Austria; 3 Vienna Biocenter PhD Program, a Doctoral School of the University of Vienna and the Medical University of Vienna, Vienna, Austria; 4 CeMM Research Center for Molecular Medicine of the Austrian Academy of Sciences, Vienna, Austria; 5 Ludwig Boltzmann Institute for Network Medicine at the University of Vienna, Vienna, Austria; 6 Research Institute of Molecular Pathology (IMP), Vienna BioCenter Campus (VBC), Vienna, Austria; 7 Medical University of Vienna, Center for Medical Biochemistry, Dr. -Bohrgasse 9, Vienna, Austria; 8 TU Wien, Faculty of Informatics, Vienna, Austria; 9 Medical University of Vienna, Vienna BioCenter (VBC), Vienna, Austria; 10 Faculty of Mathematics, University of Vienna, Vienna, Austria; 11 Present Address: Department of Biological Chemistry and Molecular Pharmacology, Harvard Medical School, Boston, MA USA; 12 Present Address: Program in Cellular and Molecular Medicine, Boston Children’s Hospital, Boston, MA USA

**Keywords:** Ubiquitin ligases, Ubiquitylation

## Abstract

APOBEC family members play crucial roles in antiviral restriction. However, certain APOBEC3 (A3) proteins drive harmful hypermutation in humans, contributing to cancer. The cancer-associated A3 proteins are capable of transiting from the cytosol to the nucleus, where they can cause genome mutations. Here, we uncover a specific set of cellular pathways that protect genomic DNA from the major cancer-associated A3 proteins. Through genetic and proteomic screening, we identify UBR4, UBR5, and HUWE1 as key ubiquitin E3 ligases marking cancer-associated A3B and A3H-I for degradation, thereby limiting A3-driven hypermutation. Mechanistically, UBR5 and HUWE1 recognize A3s in the absence of their RNA binding partner, thus promoting proteasomal degradation of APOBEC3 protein that is not engaged in its antiviral cellular function. Depletion or mutation of the E3 ligases in cells and human cancer samples increases A3-driven genome mutagenesis. Our findings reveal that UBR4, UBR5, and HUWE1 are crucial factors in a ubiquitination cascade that maintains human genome stability.

## Abstract (web summary)

APOBEC deaminases restrict retroviruses but can also mutate human DNA. Here, the authors show that cancerassociated APOBEC3s with low RNA binding, known to enter the nucleus, are selectively recognized by E3 ligases and degraded, eliminating harmful nuclear enzymes, and limiting genome mutation.

## Introduction

The human genome encodes seven members of the APOBEC3 (apolipoprotein B mRNA editing enzyme, catalytic polypeptide-like 3) family of cytidine deaminases^1^. These deaminases play a critical role in innate immunity against retro/lentiviruses by causing hypermutation of the viral cDNA^2,3^. The APOBEC3 (A3) family is under strong selective pressure in humans and other primates, with all seven members (A-H) possessing DNA C-to-U deaminase activity^4–7^. A3H is the oldest and most evolutionarily distant member of the A3 family and contains a unique zinc-coordinating motif in its deaminase domain^8,9^. In addition, it has the most haplotypes in the human population^1,10–12^. These haplotypes differ significantly in terms of their stability, subcellular localization and antiviral activity^13,14^.

In contrast to this host-beneficial function, previous studies have shown that several A3 family members can have detrimental effects in humans by driving hypermutation of cellular DNA. Such hypermutation has been documented in diverse cancer types, thereby contributing to a broader, disadvantageous mutational landscape within these tumors^12,15–18^.

Elevated levels of A3A, A3B, and A3H-I have been associated with mutagenesis in a range of cancers^15–17,19,20^. A3-mediated mutagenesis has been shown to drive some of the most prevalent mutational signatures in cancer, characterized by C-to-T transitions and clustered mutations (kataegis) at TCN trinucleotides^17,21–28^. APOBEC-associated mutational signatures have been identified in more than 70% of cancer types and around 50% of all cancer genomes^17,29,30^. These signatures are prominent in breast, lung, and bladder cancer, as well as other cancers^17,18,31–33^. A3A is overexpressed in a wide spectrum of human cancers and can induce kataegis and omikli, a form of extreme kataegis with more than 100 mutations per megabase^15–17,28,33^. A3B is overexpressed in many cancers and can generate APOBEC-specific SBS2 and SBS13 mutational signatures^17,34,35^. A3H occurs as several haplotypes in the human population, of which only the nuclear haplotype I (A3H-I) is associated with APOBEC signatures in breast and lung cancer, whereas the cytosolic haplotype II (A3H-II) is not^18,32^.

Although these A3 deaminases mutagenize ssDNA substrates, their localization and activity are controlled through binding to double-stranded secondary structures in cellular RNAs^36,37^. In infected cells, cytosolic A3H binding to secondary structures in the viral RNA genome and A3G binding to single-stranded viral RNA^38,39^ is essential for packaging into progeny virions, and subsequent mutagenesis of the viral cDNA during reverse transcription^12–14,40,41^. A3F, A3G, and A3H-II have been reported to have strong virus restrictive properties^10–13^. Importantly, these A3 proteins are not turned-over by the proteasome, and as a consequence of their stability, accumulate at high steady-state intracellular protein concentrations^10–14,40^. In contrast, the A3 members that have been associated with hypermutation signatures in various cancers, such as A3A, A3B, and A3H-I, are predominantly nuclear. These A3s are rapidly turned-over by the proteasome, and consequently are present at low intracellular protein concentrations^15–17,19,20^. A3H-I instability is determined by a single nucleotide polymorphism (SNP), causing a R105G mutation which is associated with increased nuclear localization^18^. It remains unclear how this SNP results in increased nuclear localization and instability. Nuclear localization of A3A, A3B, and A3H-I has been proposed to contribute to cancer hypermutation as it promotes access to genomic DNA^18^.

The unstable A3 family members, though usually present at low nuclear levels, play a greater role in generating cancer-linked APOBEC mutational signatures than the more stable cytosolic variants^18,28^. However, it is unclear how the former group of A3s is more closely associated with cancer given their instability. We hypothesized that: (i) deregulation of the limited protein concentrations of the unstable nuclear A3 members is likely sufficient to drive mutagenesis in cancers, (ii) there are unidentified cellular factors that, under physiological conditions, maintain low nuclear A3 protein levels through active degradation, and (iii) these cellular factors protect against cell-intrinsic genome mutagenesis by specifically keeping the cellular concentrations of potentially harmful nuclear A3 variants low. In addition, we predicted that the absence of these unidentified “guardian” factors would unleash hypermutation through increased nuclear A3 protein levels that compromise host genome integrity.

Through genetic screening and proximity proteomics, we identified UBR4, UBR5, and HUWE1 as E3 ligases that ubiquitinate A3B and A3H-I, thereby targeting them for proteasomal degradation. Consistent with their genome-guardian roles, ablation or mutation of these E3 ligases in cancer cell lines and human cancer samples led to increased APOBEC3-driven hypermutation.

## Results

### Proteasomal degradation controls protein levels of cancer-associated A3s

**Fig. 1:**
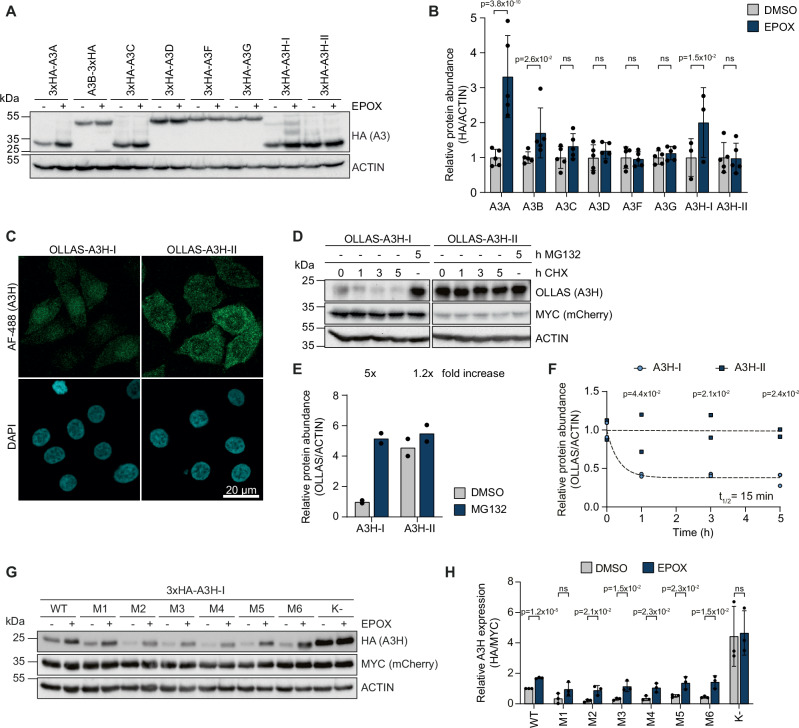
Proteasomal degradation controls protein levels of cancer-associated A3s. **A** HEK-293T cells were transfected with different amounts of 3 x HA-tagged A3 expressing plasmids to achieve similar steady-state A3 protein levels. 24 h. post transfection, cells were treated for 16 h. with EPOX, and protein levels were analyzed by WB, and **B** quantified (means and SD, 2-way ANOVA, ns: p ≥ 0.05, n = 5 (for A3H-I n = 3). **C** RKO cells stably expressing OLLAS-A3H-I/II were fixed, and their subcellular localization determined by immunofluorescence confocal microscopy; scale bar: 20 µm. **D**–**F** Lentiviral expression constructs encoding A3H-I or A3H-II were delivered to RKO cells at different integration rates to obtain comparable A3H-I and A3H-II protein levels in the presence of proteasome inhibitor. Polyclonal cell pools were treated with CHX or MG132 for the indicated times, **D** protein levels analyzed by WB, **E** relative A3H-I and A3H-II protein levels quantified by densitometry (n = 2 biological replicates), and **F** single-step exponential decay curves were calculated, from which protein half-life was derived (means and SD, two-way ANOVA, corrected for multiple comparisons using the Šídák method, n = 2). **G**, **H** HEK-293T cells were transfected with equal amounts of plasmids encoding the indicated MYC-mCherry-P2A-3xHA-tagged A3H-I mutants, in which multiple lysine residues were mutated to arginine. 36 h. Post-transfection, cells were treated with EPOX for 5 h. **G** protein levels determined by WB, and **H** quantified (means and SD, multiple unpaired t-tests (two-sided), not corrected for multiple hypothesis testing, ns: p ≥ 0.05, n = 3). Source data are provided as a Source data file.

Since the cancer-associated A3 family members, A3A, A3B, and A3H-I, only accumulate to low steady-state levels, we hypothesized that the cellular concentrations of these factors are controlled by protein degradation. To test this, constructs encoding all human A3 family members were delivered and expressed at similar steady-state protein levels (Fig. 1A). Subsequently, these cells were treated with the proteasome inhibitor epoxomicin (EPOX), and the effect on the various A3 proteins was determined by Western Blot (WB) analysis (Fig. 1A, B). Inhibition of proteasomal degradation significantly increased protein concentrations of the nuclear and cancer-associated A3A, A3B, and A3H-I proteins, indicating that their intracellular protein levels are substantially determined by proteasomal degradation. In contrast, protein levels of cytoplasmic A3s (A3D, A3F, A3G, and A3H-II), which are important for the innate immune response against retro/lentiviruses, were unaffected (Fig. 1A, B).

To further study the cellular mechanisms governing proteasomal degradation of nuclear, cancer-associated A3 protein levels in cells, we decided to use A3H as a model. The two predominant human A3H haplotypes represent each of the two identified phenotypes: A3H-I is nuclear, cancer-associated, and turned over by the proteasome, whereas A3H-II is cytoplasmic and stable. Stability differences are reflected in reduced accumulation of A3H-I steady-state protein levels, as described for the endogenous protein in primary T lymphocytes^42^. This difference in stability is phenocopied upon exogenous expression in a wide variety of cell lines^12,42,43^. In line with previous reports^13,14,44^, some exogenous A3H-I localized to the cytosol but a substantial portion was nuclear, whereas A3H-II was mostly cytosolic (Fig. 1C).

Subsequently, we set out to measure the protein stability of A3H-I and A3H-II. To this end, we generated polyclonal RKO (human colon carcinoma) and HeLa (human cervical adenocarcinoma) cell lines expressing a stable myc-tagged mCherry internal control, and either A3H-I or A3H-II through a P2A ribosomal skip site (Supplementary Fig. 1A). To compensate for the differences in steady-state protein levels of the two A3H haplotypes, A3H-I cells were transduced with a higher virus-like particle concentration, and, therefore, express more of the mCherry control (Fig. 1D). A3H protein stability was then determined in a chase experiment with the translation inhibitor cycloheximide (CHX), or in the presence of proteasome inhibitor MG132 (Fig. 1D and Supplementary Fig. 1B). Consistent with rapid proteasome-mediated turnover, A3H-I protein accumulated at much lower steady-state levels than A3H-II and was rapidly depleted in the presence of cycloheximide (Fig. 1D and Supplementary Fig. 1B, compare lanes 1 and 6). These levels were increased 4- to 5-fold upon proteasome inhibition (Fig. 1E and S1C). Confirming their different stabilities, A3H-I was degraded with a half-life of 15 min. in RKO cells (Fig. 1F), and 10 min. in HeLa cells (Supplementary Fig. 1D), whereas A3H-II remained stable during the 5 h. chase period.

A3H-I degradation was exclusively dependent on proteasomal degradation, as steady-state protein levels of the two A3H haplotypes were unaffected by inhibitors of autophagy/lysosomal degradation (Supplementary Fig. 1E). Likewise, no differences in mRNA stability in the presence of Actinomycin D (ActD) (Supplementary Fig. 1F), nor secretion (Supplementary Fig. 1G) were measured between the two A3H haplotypes, indicating that A3H-I protein levels were predominantly regulated through proteasomal degradation.

Consistent with this result, A3H-I and A3B (Fig. 1A, B) were ubiquitinated (Supplementary Fig. 1H, I). Interestingly, while A3A was highly unstable (Fig. 1A, B), we consistently found it to be minimally ubiquitinated (Supplementary Fig. 1H, I), which suggested that it may be regulated through a different - possibly ubiquitin-independent - mechanism than A3B and A3H-I.

To identify the residues important for A3H-I turnover, multiple lysine (K) residues in A3H-I were systematically grouped and mutated to arginine (R), based on their position in the A3H structure (Supplementary Fig. 1J, K). All of these clustered K-to-R mutants accumulated at low steady-state protein levels, and were stabilized by proteasome inhibition, indicating that multiple lysines in different structural regions of A3H-I are likely important for its ubiquitination and degradation (Fig. 1G, H, Supplementary Fig. 1A). In agreement with this conclusion and published data^45^, when all lysine residues were mutated to arginine, A3H-I accumulated at 4-fold higher steady-state protein concentrations and were no longer affected by proteasome inhibition (Fig. 1G, H).

Together, these data indicate that protein levels of the nuclear, cancer-associated A3s, A3A, A3B, and A3H-I are regulated through proteasomal degradation, and that degradation of the model protein A3H-I depends on ubiquitination of multiple lysine residues.

### The E3 ligases UBR4, UBR5, and HUWE1 independently mediate turnover of A3B and A3H-I

The results described above positioned A3H-I as an excellent model to identify the cellular machinery that degrades it (and possibly other cancer-associated A3s), allowing its naturally occurring non-cancer-associated variant -A3H-II- to be used as a stable control to determine specificity.

**Fig. 2:**
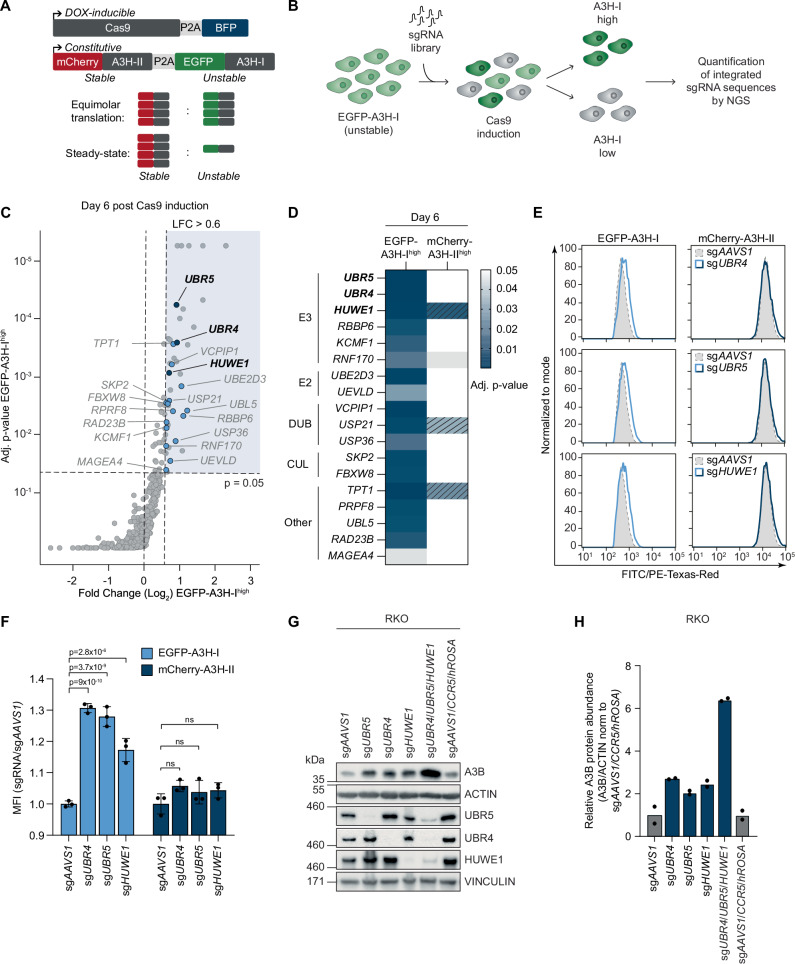
The E3 ligases UBR4, UBR5, and HUWE1 independently mediate turnover of A3B and A3H-I. **A** A monoclonal RKO cell line expressing DOX-inducible Cas9-P2A-BFP and a constitutive mCherry-A3H-II-P2A-EGFP-A3H-I dual reporter was generated (RKO-DOX-Cas9-dualA3H). EGFP-A3H-I and mCherry-A3H-II are synthesized in equimolar amounts, but EGFP-A3H-I shows low steady-state levels due to proteasomal degradation. **B** Schematic of the CRISPR/Cas9 FACS-based screening strategy. Cells were transduced with an sgRNA library targeting ubiquitin-proteasome and autophagy-related genes, selected with G418, induced with DOX for 3 or 6 days, and sorted for the top and bottom 1–2% of EGFP or mCherry fluorescence. sgRNA abundance was determined by next-generation sequencing and compared to unsorted controls. **C** Genes enriched in EGFP-A3H-I^high^ populations at day 6 post induction, with adjusted p-values from MaGECK FDR analysis of three independent replicate sorts. **D** Heatmap of top genes on log_2_ fold-change and p-value grouped by functional categories. Genes enriched in EGFP-A3H-I^high^ cell populations 6 days post Cas9 induction with a log_2_ fold-change >0.6, which were not enriched in mCherry^high^ or GFP^low^ on either day 3 (LFC > 0.45) or day 6 (LFC > 0.6). Adjusted p-values are based on MaGECK FDR analysis of three independent replicate sorts. Dashed lines indicate a log_2_ fold-change <0.6. **E** RKO-DOX-Cas9-dualA3H cells were transduced with individual sgRNAs, induced with DOX for 6 days, and analyzed for EGFP-A3H-I and mCherry-A3H-II mean fluorescence intensity by flow cytometry, with **F** quantification (means and SD, two-way ANOVA with Šídák correction, ns: p ≥ 0.05, n = 3). **G** RKO cells expressing DOX-inducible Cas9 were transduced with sgRNAs targeting UBR4, UBR5, or HUWE1, individually or in combination. Following 6 days of DOX treatment, endogenous A3B protein levels were assessed by WB and **H** quantified (n = 2 biological replicates). Source data are provided as a Source data file.

To identify specific protein stability regulators of A3H-I, but not A3H-II, we set up a CRISPR-based genetic screening platform^46–48^. First, an RKO cell line was established harboring a doxycycline (DOX)-inducible Cas9-P2A-BFP construct, and an mCherry-A3H-II-P2A-GFP-A3H-I dual reporter (Dual-A3H-reporter), driven from an exogenous promoter (Fig. 2A). Inhibitor treatments confirmed that the two fluorophore-tagged A3H fusion proteins phenocopied the stability pattern of their untagged counterparts (Supplementary Fig. 2A).

To screen for regulators of A3H-I stability, the Dual-A3H-reporter cell line was transduced with a ubiquitin-focused lentiviral sgRNA library, Cas9 expression was induced, and cells with the highest or lowest 1–2% EGFP or mCherry fluorescence (EGFP^high^, mCherry^high^, EGFP^low^, and mCherry^low^) were collected after 3 and 6 days. Genomic DNA was isolated from the collected cells, and integrated sgRNA-coding sequences quantified by NGS (Fig. 2B, Supplementary Data 1).

Several sgRNAs were specifically enriched in EGFP-A3H-I^high^ sorted cells, which did not score in the mCherry-A3H-II^high^ sorted pool (Fig. 2C, D). Among the top hits were sgRNAs targeting several E3 ligases, E2 conjugating enzymes, DUBs/proteases, and other degradation-associated genes. The top E3 ligases scored on day 6 were *UBR4*, *UBR5*, and *HUWE1. HUWE1* was also one of the top hits on day 3 (Supplementary Fig. 2B, C). Interestingly, we also found the gene encoding the E2 conjugating enzyme UBE2D3, an E2 conjugating enzyme known to work with UBR5 and HUWE1, as one of the top hits for both days (Fig. 2C, D and S2C, D). These results identified UBR4, UBR5, and HUWE1 as strong, specific candidates mediating A3H-I degradation.

To test the validity and specificity of the screen results, each of these E3 ligases was targeted in isolation. Individual ablation of each of the E3 ligases significantly increased EGFP-A3H-I levels in the Dual-A3H-reporter screening cell line (Fig. 2E, F), as well as a polyclonal cell pool expressing the same construct (Supplementary Fig. 2D), whereas mCherry-A3H-II fluorescence was not affected. We further tested whether the increased A3H-I steady-state protein levels in the E3 ligase knock-out cells stemmed from increased protein stability by inhibiting translation with CHX. Therefore, we generated RKO-DOX-Cas9-MYC-mCherry-P2A-3xHA-A3H-I cells harboring a triple-E3 ligase knock-out, treated them with CHX, and monitored A3H-I protein levels over time by WB (Supplementary Fig. 2E, F). Ablation of all three E3 ligases increased A3H-I half-life from 21 min.to 1.9 h. (Supplementary Fig. 2F), indicating that A3H-I degradation is compromised in the absence of UBR4/UBR5/HUWE1.

Since we found that A3B is also turned over in a proteasome-dependent manner (Fig. 1A, B), we reasoned that the same cellular machinery may be employed to keep its steady-state levels low. Therefore, we set out to determine whether *UBR4*, *UBR5*, or *HUWE1* ablation would also influence the abundance of A3B. Individual knock-out of *UBR4*, *UBR5*, and *HUWE1*, increased endogenous A3B protein levels by 2.5–4-fold in RKO cells (Fig. 2G, H) as well as in THP-1 monocytic cells (Supplementary Fig. 2G, H), without affecting its mRNA levels (Supplementary Fig. 2I). Consistent with the hypothesis that the E3 ligases specifically degrade nuclear A3 members, the predominantly cytoplasmic endogenous A3G remained unaffected by the E3 knock-outs in THP-1 cells (Supplementary Fig. 2G, H). In RKO cells, stability of endogenous A3G could not be evaluated due to low A3G expression in these cells.

Importantly, knock-out of each of the individual ligases increased endogenous A3B protein levels by 2-fold in each case (Fig. 2G, H), whereas simultaneous targeting of *UBR4*, *UBR5*, and *HUWE1* strongly increased A3B levels in an additive fashion to 6-fold in comparison to its matching controls in which three safe harbor loci were targeted (Fig. 2G, H). From these non-epistatic genetic interactions, we concluded that UBR4, UBR5, and HUWE1 each target A3B independently of the other two E3 ligases.

Collectively, these results identified the E3 ligases UBR4, UBR5, and HUWE1 as important functional effectors of A3H-I degradation. This degradation-dependence is conserved for cancer-associated A3B and is relevant in different human cell types. Our results indicate that the three E3 ligases likely function redundantly, and thus poly-ubiquitinate A3B, and possibly also A3H-I, independently of each other.

A3A has been shown to also drive mutagenesis in cancer^15,17,21,33,49^. Since we found that A3A is also highly unstable (Fig. 1A, B), we asked whether A3A was likewise targeted for degradation by UBR4, UBR5, and HUWE1. Unlike A3B and A3H-I, A3A steady-state protein levels were not increased upon targeting of UBR5 or HUWE1 (Supplementary Fig. 3A, B). In contrast, ablation of UBR4 did stabilize A3A (Supplementary Fig. 3A, B). Together with the observation that A3A itself is not substantially ubiquitinated (Supplementary Fig. 1H, I), this indicates that the machinery for marking A3A for degradation is different from A3B and A3H-I.

To get a better understanding of A3A degradation, cells were treated with various inhibitors of ubiquitination and degradation pathways (Supplementary Fig. 3C, D). These results indicate that A3A is exclusively degraded by the proteasome, without major contributions of the lysosomal pathway. Moreover, inhibition of the ubiquitin activating E1 enzyme by TAK-243 or VCP by CB-5083 stabilized A3A. This showed that while A3A is itself not substantially ubiquitinated, it is dependent on ubiquitination and VCP for its turn-over, possibly by co-degradation of a ubiquitinated binding partner.

Taken together, these data indicate that A3A is itself not majorly ubiquitinated, yet degraded in a ubiquitin-dependent manner. Our data indicate that the degradation machinery of A3A differs from A3B/A3H-I. Instead of UBR5 and HUWE1, A3A requires unknown other E3 ligases and VCP for its turn-over. All three unstable APOBEC3 members require UBR4, which we speculate may be in a ubiquitin-chain extending (E4 ligase) capacity.

### UBR5 and HUWE1 form a complex with A3H-I and other unstable A3 deaminases in cells

The above data indicate that UBR4, UBR5, and HUWE1, identified through our specific genetic screen, can directly or indirectly mediate A3 degradation. To complement the genetic screen, we set out to identify specific physical interaction partners of the unstable A3H-I. We reasoned that overlapping factors identified in both approaches would be strong candidates for factors that directly recognize A3B and A3H-I as ubiquitination substrates.

**Fig. 3:**
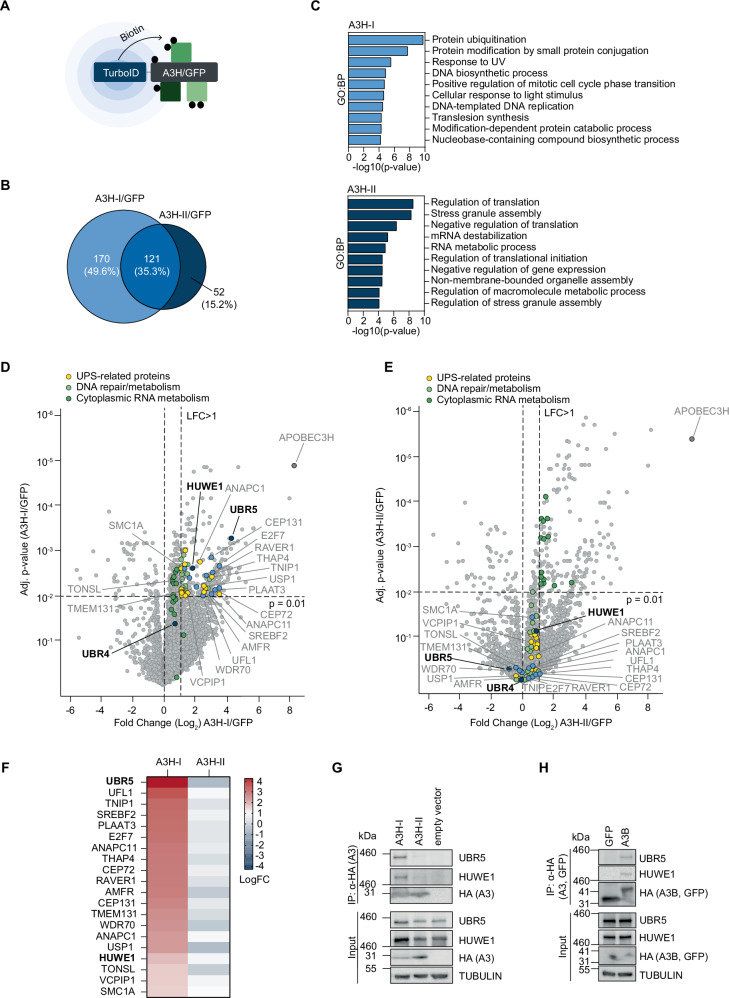
UBR5 and HUWE1 form a complex with A3H-I and other unstable A3 deaminases in cells. **A** Overview of TurboID principle. **B**–**F** Polyclonal RKO-DOX-TID-A3H-I/II/GFP were treated with DOX for 2 days to achieve similar protein levels. Cells were treated with EPOX for 5 h. and supplemented with biotin during the last 15 min. Biotinylated proteins were purified under denaturing conditions and quantified by nLC-MS/MS (mean and SD, n  =  3 biological replicates, moderated t-statistics via the limma-trend method with Benjamini–Hochberg multiple testing correction). **B** Differentially enriched proteins in A3H-I/GFP (light blue, LFC > 1, p-value < 0.01) and A3H-II/GFP (dark blue, LFC > 1, p-value < 0.01) were compared. **C** GO terms for biological processes (GO:BP) of differentially enriched proteins in A3H-I/GFP (light blue, LFC > 1, p-value < 0.01, input: 170 factors derived from **B**) and A3H-II/GFP (dark blue, LFC > 1, p-value < 0.01, input: 52 factors derived from **B**). **D** Differential expression of TID-A3H-I, or (**E**) TID-A3H-II interactors relative to TID-GFP (n = 3). Light blue dots mark factors of enriched proteins in TID-A3H-I samples relative to TID-GFP. Highlighted are the top 20 A3H-I-specific proteins displayed according to the following criteria: A3H-I/GFP LFC > 1, p-value < 0.01, which are not enriched in A3H-II/GFP LFC > 1, p-value < 0.01. **F** Heatmap of enriched proteins in TID-A3H-I samples, or TID-A3H-II samples relative to TID-GFP. Top 20 A3H-I-specific proteins displayed (A3H-I/GFP LFC > 1, p-value < 0.01), which are not enriched in A3H-II/GFP (LFC > 1, p-value < 0.01). HEK-293T cells transfected with different amounts of plasmids encoding **G** 3xHA-A3H-I/II, or **H** 3xHA-GFP or A3B-3xHA to achieve similar steady-state protein levels. 3xHA-tagged proteins were immunoprecipitated, and their interaction with endogenous UBR5 and HUWE1 determined by WB. Source data are provided as a Source data file.

For unbiased identification of specific A3H-I interactors, TurboID (TID) proximity labeling proteomics was performed (Fig. 3A). Three different RKO cell lines stably expressing either DOX-inducible (a) TID-A3H-I, (b) TID-A3H-II, or (c) TID-GFP as a control were generated. Similar expression of each of the three constructs was achieved by inducing transgene expression with different concentrations of DOX. Subsequently, proteins proximal to the TurboID fusion proteins were covalently labeled by the addition of biotin, after which biotinylated proteins were isolated and identified by nLC-MS/MS (Supplementary Data 2).

We first compared the interactomes of A3H-I and A3H-II relative to the TID-GFP control. In line with a recent publication^50^, both A3H haplotypes share many interactors (Fig. 3B), mainly comprising Gene Ontology (GO) terms for biological processes representing regulation of translation, RNA metabolism, and RNA processing (Supplementary Fig. 4A), which are amongst the top scoring hits in both A3H haplotypes.

However, analysis of A3H-I-specific interactors identified protein ubiquitination and DNA-metabolism related processes as the top ranked biological process GO terms (Fig. 3C). Processes related to A3H-II-specific included translation, stress granule assembly, and RNA metabolic processes (Fig. 3C), terms which are partially shared with the A3H-I interactome (Supplementary Fig. 4A). This reinforces the above results, which indicate that only A3H-I is targeted by the ubiquitin-proteasome system. In addition, the enrichment for DNA-metabolism related processes indicates that A3H-I may have access to DNA. As expected, top-ranked GO terms for cellular compartments were enriched for nuclear terms in the case of the A3H-I-specific interactome. In contrast, A3H-II-linked terms predominantly included cytoplasmic stress granules and P-bodies (Supplementary Fig. 4B), underlining the propensity of stable A3s to form high-molecular-weight RNP complexes^51–54^.

Consistent with a high degree of shared interaction partners, most interactors were not unique to either the A3H-I/GFP or A3H-II/GFP haplotype (Fig. 3D, E; unlabeled data points in upper-right quadrant). Amongst the specific proteins significantly enriched in the A3H-I samples were the E3 ligases UBR5 and HUWE1 (Fig. 3D–F), which were also identified as specific genetic interactors of A3H-I (Fig. 2C, D). Although UBR4 peptides were detected by nLC-MS/MS, they were not significantly enriched in any one sample. In line with these MS results, WB analysis detected UBR5 and HUWE1 in streptavidin-enriched lysates from TID-A3H-I expressing cells, but not in lysates from TID-A3H-II or TID-GFP controls (Supplementary Fig. 4C). Consistent with the proximity labeling results, endogenous UBR5 and HUWE1 specifically interacted with exogenously expressed A3H-I (Fig. 3G) and A3B (Fig. 3H) but did neither co-IP with the stable A3H-II haplotype (Fig. 3G), nor A3A (Supplementary Fig. 4d).

Together, these data show that UBR5 and HUWE1 specifically interact with A3B and A3H-I in cells and potentially ubiquitinate them directly. In contrast, UBR4 was not identified as a specific A3B/A3H-I interactor in cells. UBR4 could either affect A3B and A3H-I stability indirectly, or its interaction with these A3s might be too transient to detect in cell-based assays.

### RNA binding protects A3s from E3 ligase binding and ubiquitination, thereby promoting their stability in cells

RNA binding plays an important regulatory role in A3 localization and antiviral activity^41,51,55,56^. The antiviral family members A3H-II and A3G are retained in the cytoplasm through RNA binding^41,51,55,56^. A3H haplotype I and II differ by three amino acids (Supplementary Fig. 5A), with a single glycine at position 105 determining the difference in stability^14,18,32^. In line with these data, mutation of G105R in A3H-I turns it from unstable and partially nuclear, to stable and predominantly cytosolic, phenocopying A3H-II (Supplementary Fig. 5B–D). Conversely, R105G mutation renders A3H-II unstable and partially nuclear, thereby phenocopying A3H-I (Supplementary Fig. 5B–D). Previous work has suggested that the R105G mutation may render the A3H protein less capable of binding dsRNA structures, leading to diminished antiviral potential^14,18,32^. Furthermore, introducing the G105R into A3H-I strongly reduced its ubiquitination (Supplementary Fig. 5E) and interaction with UBR5 and HUWE1 (Supplementary Fig. 5F). Conversely, the A3H-II R105G mutation resulted in its ubiquitination (Supplementary Fig. 5E) and association with UBR5 and HUWE1 (Supplementary Fig. 5F).

**Fig. 4:**
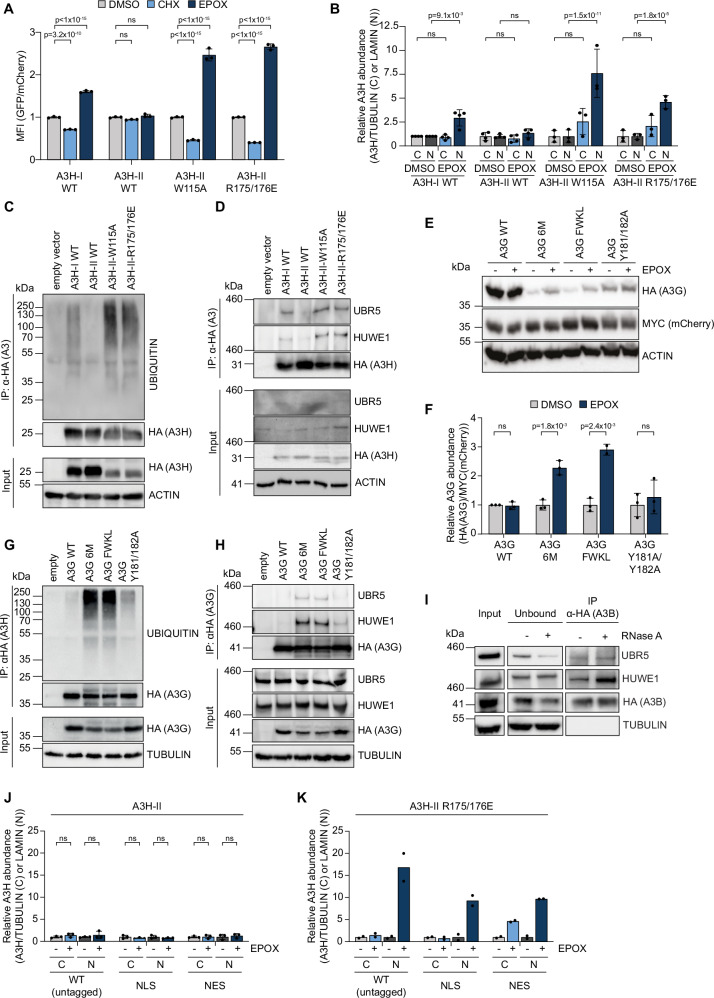
RNA binding protects A3s from E3 ligase binding and ubiquitination, thereby promoting their stability in cells. **A** RKO-mCherry-P2A-EGFP-A3H cells expressing the indicated EGFP-tagged A3H variants were treated for 5 h with EPOX or CHX. mCherry and EGFP-A3H MFI was measured by flowcytometry (means and SD, two-way ANOVA with Tukey-correction, ns: p ≥ 0.05, n = 3). **B** HEK-293T cells were transfected with varying amounts of plasmids encoding 3xHA-tagged A3H to achieve comparable protein levels. After 5 h of EPOX treatment, sub-cellular fractions were analyzed by WB and quantified (means and SD, two-way ANOVA with Tukey correction, ns: p ≥ 0.05, n = 3). HEK-293T cells were transfected as in (**B**), treated with EPOX, and 3xHA-tagged proteins were immunoprecipitated to assess (**C**) ubiquitination or (**D**) interaction with UBR5 and HUWE1 by WB. **E**, **F** HEK-293T cells transiently expressing mCherry-P2A-3xHA-tagged A3G WT or RNA-binding mutants were treated with EPOX for 5 h, followed by WB analysis and quantification (means and SD, multiple unpaired two-sided t-tests with Šídák correction, ns: p ≥ 0.05, n = 3). **G**, **H** Cells were transfected as in (**E**) with adjusted plasmid amounts to equalize protein levels, followed by EPOX treatment and immunoprecipitation to assess (**G**) ubiquitination or (**H**) interaction with UBR5 and HUWE1. **I** HEK-293T cells expressing A3B-3xHA were treated with EPOX, with or without RNase A treatment prior to immunoprecipitation. **J**, **K** HEK-293T cells were transfected with different amounts of plasmids expressing the indicated 3xHA-tagged A3H constructs to achieve similar steady-state protein levels. Following 5 h. of EPOX treatment, cellular fractions were extracted, analyzed by WB, and quantified (means and SD, 2-way ANOVA; not corrected for multiple comparisons, ns: p ≥ 0.05, n = 3 biological replicates for (**J**), n = 2 biological replicates for (**K**)). Source data are provided as a Source data file.

Based on these and prior observations^14,18,32^, we hypothesized that reduced RNA binding leads to the re-localization of cytoplasmic A3H-II to the nucleus and renders it a substrate for proteasomal degradation. Therefore, we tested previously characterized A3H-II mutants (W115A and R175/176E)^41^, which harbor mutations in the α6 helix and the structurally adjacent loop 1, and impair RNA binding. In line with previous reports^41,51,55,56^, diminished RNA binding decreased cytosolic A3H-II levels and increased nuclear localization (Supplementary Fig. 5G). In contrast to the stable wild-type (WT) A3H-II, its RNA-binding mutants were highly unstable: their levels decreased rapidly upon translation inhibition yet stabilized in the presence of a proteasome inhibitor (Fig. 4A, Supplementary Fig. 5H). These results indicated that RNA binding affects two characteristics of cellular A3: (i) retention in the cytosol, and (ii) it prevents its degradation.

Next, we asked in which compartment the respective A3H mutants are degraded. For this experiment, A3H WT and RNA-binding mutant constructs were expressed at similar levels, after which their accumulation upon proteasome inhibition was assessed in the nuclear and cytosolic cell fractions (Fig. 4B, Supplementary Fig. 5I). As expected, A3H-II protein levels in whole cell extracts were not affected by proteasome inhibition, whereas it significantly increased the levels of the unstable A3H-I and A3H-II RNA-binding mutants (Supplementary Fig. 5J, K). Analysis of fractionated samples showed that A3H-I and the RNA-binding mutants of A3H-II predominantly accumulated in the nucleus when their degradation was blocked (Fig. 4B, Supplementary Fig. 5I), indicating that unstable A3H variants are degraded in the nucleus. Moreover, RNA-binding mutants of A3H-II were strongly ubiquitinated (Fig. 4C) and co-immunoprecipitated with UBR5 and HUWE1 (Fig. 4D), indicating that loss of RNA binding renders A3H-II a substrate for degradation by the identified E3 ligases.

These data indicated that RNA binding may be a general mechanism by which A3 stability is regulated. To test this hypothesis, we analyzed the stability of WT A3G compared to three A3G RNA-binding mutants^57^ in cells. Consistent with comparable RNA-binding mechanisms across the A3 family, structural alignment with A3H showed that the helix region of A3G CD1-containing mutations superimposed with the α6 helix of A3H-II, indicating a probable determinant for RNA binding (Supplementary Fig. 6A). Mutations in A3G residues F126, W127, K180, L184 have been previously shown to abolish its ability to bind nucleic acids^58^. Moreover, residues F126 and W127 have been shown to form an essential purine nucleotide binding pocket for RNA, and to be key for A3G packaging into virions and viral restriction^39,59,60^.

In contrast to WT A3G, steady-state protein levels of A3G RNA-binding mutants localized partially to the nucleus (Supplementary Fig. 6B–D), which increased upon proteasome inhibition (Fig. 4E, F, Supplementary Fig. 6E, F), underlining the importance of the integrity of this helix, its ability to bind RNA, and the formation of high molecular weight complexes for protein stability. Notably, A3G-Y181/182 A was stabilized less upon EPOX treatment (Fig. 4E, F), possibly resulting from incomplete disruption of the α-helix by these mutations. Similar to A3H, also levels of RNA-binding mutants of A3G increased only in the nuclear compartment (Supplementary Fig. 6C, D).

In agreement with the above data, which indicate that RNA binding prevents recognition of A3 as substrates for degradation, the A3G 6 M and FWKL mutants were ubiquitinated (Fig. 4G) and co-immunoprecipitated with UBR5 and HUWE1 (Fig. 4H), whereas their WT counterparts did not. Lower levels of A3G Y181/182 A ubiquitination and E3 interaction (Fig. 4G, H) correlated with reduced instability of this mutant compared to WT protein (Fig. 4E, F), potentially stemming from differences in their RNA-binding.

These and previously published results indicated that RNA binding by A3H and possibly other APOBEC3 proteins determines both their cytosolic localization and stability^44^. We hypothesized that there is an intra-cellular equilibrium between RNA-bound (stable) and non-bound (unstable) fractions of A3H-I and possibly also A3B. Even though UBR5 and HUWE1 co-immunoprecipitated with A3B already in the absence of RNase A treatment (Fig. 3H), we reasoned that treatment of cellular extracts with RNAse A would additionally remove cellular RNA from the cellular A3B pool, and allow increased binding of A3B as a substrate by UBR5 and HUWE1. To test this, RKO cells expressing HA-tagged A3B were lysed and incubated with RNase A, after which HA-A3B was IPed, and UBR5 and HUWE1 interaction detected by WB (Fig. 4I). Consistent with our hypothesis that part of the cellular A3B protein pool is stable through engagement of cellular RNA, RNAse A treatment increased both UBR5 and HUWE1 co-IP with A3B (Fig. 4I). These data suggested that part of the cellular A3B pool is stabilized through RNA binding, as it prevents recognition by UBR5 and HUWE1, whereas the unbound cellular pool is targeted for degradation.

These findings and those of other studies indicated that RNA binding affects both localization and stability of A3H and possibly other APOBEC3 proteins^44^. This makes it difficult to determine whether one of the two effects, or both, determines APOBEC3 turn-over. To address this, we asked whether forced nuclear localization of A3H-II by fusion to an NLS was sufficient to render it unstable, and vice versa, whether re-localization of an unstable A3H-II RNA binding mutant to the cytoplasm via an NES-fusion was sufficient to stabilize it. Fusion of either an NLS or NES did not affect A3H-II stability (Supplementary Fig. 6G–I), and thus remained mostly inconclusive as neither of the localization signals effectively superseded the predominantly cytosolic localization or stability of wildtype A3H-II. In contrast, NLS and NES fusions of the unstable A3H-II R175/176E RNA binding mutant caused a partial re-localization of the protein pools to the intended compartments (Supplementary Fig. 6J–L). In both cases, EPOX treatment stabilized both the increased nuclear and cytosolic protein pools (Fig. 4J, K, Supplementary Fig. 6J–L), indicating that the A3H-II RNA binding mutant is likely unstable irrespective of its cellular localization.

This suggests that the reduced stability of the RNA binding mutant is predominantly determined by its exposed RNA binding interface, rather than the sub-cellular localization. These findings are thus most consistent with a model in which unstable A3H variants are recognized by UBR5 and HUWE1 in their non-RNA-bound state, whereas the inherently coupled change in localization plays less of a role in its stability. However, it should be noted that because of the experimental inability to fully re-localize the A3H proteins, we cannot rule out that substrate localization contributes degradation rates.

Collectively, these results show that the RNA-binding ability of A3H-II and A3G are important for their stability. Absence of RNA binding enables UBR5 and HUWE1 engagement, ubiquitination, and eventual proteasomal degradation in cells.

### RNA binding by A3B and A3H proteins determines their recognition and ubiquitination by E3 ligases in vitro

Cell-based assays showed that impaired RNA binding renders A3 proteins substrates for proteasomal degradation. Therefore, we hypothesized that the identified E3 ligases recognize the unoccupied RNA-binding surface of A3 proteins, and that RNA binding thus prevents ubiquitination. However, RNA binding has two confounding effects in cells as it influences both A3 localization, and stability. To test the hypothesis that the E3 ligases recognize unengaged RNA-binding interfaces, we therefore set out to analyze direct E3 ligase targeting of A3H in a cell-free in vitro reconstituted ubiquitination system.

**Fig. 5:**
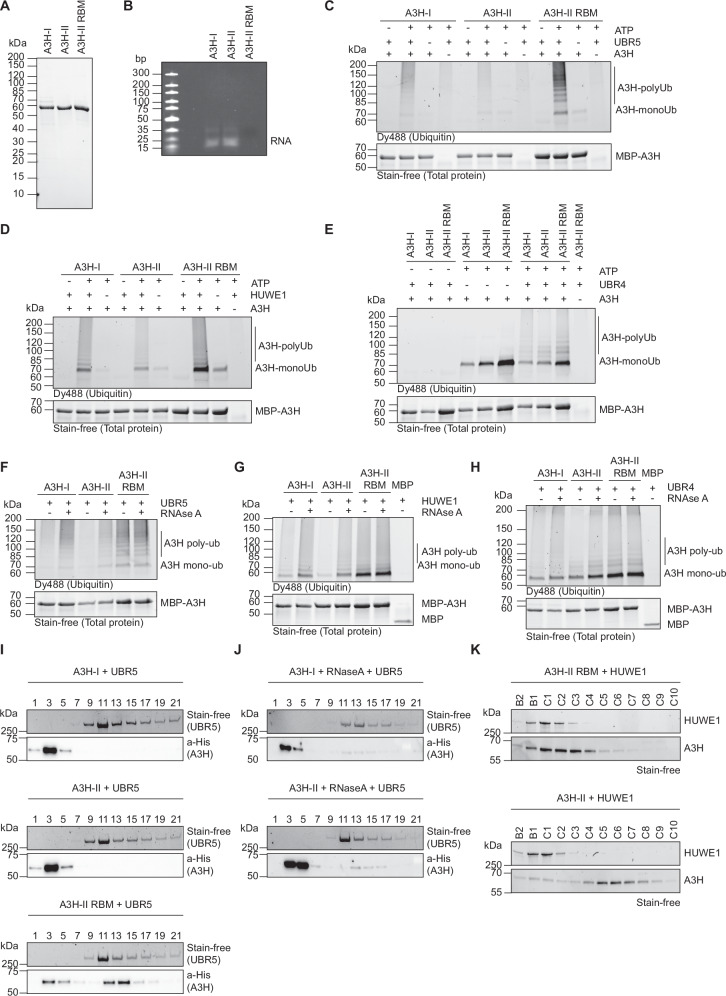
RNA binding by A3B and A3H proteins prevents their recognition and ubiquitination by E3 ligases in vitro. **A** 10x-His-MBP-A3H-I, 10x-His-MBP-A3H-II and 10xHis-MBP-A3H-II-RBM were expressed in *E. coli*, purified by HisTrap and subsequent gel filtration, after which purified proteins were analyzed by PAGE and Coomassie staining. **B** Purified proteins were analyzed by denaturing Urea-TBE PAGE followed by SYBR Gold staining. In vitro ubiquitination assays were performed with recombinant **C** UBR5, **D** HUWE1, or **E** UBR4 and WT A3H-I/II or A3H-RBM as substrates, and in the presence of DyLight488-labeled recombinant ubiquitin. Subsequently, A3H was immunoprecipitated using anti-MBP-coupled beads and the ubiquitination pattern visualized by fluorescent imaging for DyLight488. In vitro ubiquitination assay with recombinant **F** UBR5, **G** HUWE1, **H** UBR4 and A3H-I/II WT or A3H-RBM in the absence or presence of RNase A. Ubiquitinated A3H was visualized as in (**C**–**E**). **I**, **J** Sucrose gradient binding assays of UBR5 and recombinant A3H proteins. **J** Recombinant A3H was pre-incubated with RNase A. **K** Analytical size exclusion chromatography of C-terminally tagged hHUWE1 (inactive) and recombinant A3H. Source data are provided as a Source data file.

First, A3H-I and A3H-II were purified from *E. coli* as previously described^41,55,61,62^. Despite several attempts during purification to remove co-purified bacterial RNA bound to A3H-I WT and A3H-II WT with high salt or extensive RNase A treatment, we were unable to purify these proteins without any residual bound RNA, as both proteins became insoluble and precipitated upon complete removal of RNA (not shown). For that reason, we additionally purified an A3H-II RNA-binding mutant (A3H-II-RBM E56A/ W115A/ R175E/ R176E) from *E. coli*, which has been previously reported to purify as an RNA-free monomer (Fig. 5A)^41^. In cells, this mutant phenocopied the stability of other A3H-II RNA-binding mutants (W115A and R175E/176E) (Supplementary Fig. 7A).

A3H-I eluted exclusively as a monomer in size exclusion chromatography (SEC), whereas A3H-II eluted both as monomer and dimer (Supplementary Fig. 7B). However, both proteins were bound to substantial amounts of short RNA fragments, as visualized by denaturing PAGE analysis (Fig. 5B), consistent with previous reports^41,55,61,62^. In contrast, A3H-II-RBM eluted as a monomer, free from any bound RNA (Fig. 5B and Supplementary Fig. 7B). The SEC data show that all purified A3H substrates were soluble.

Our cell-based data suggested that UBR5, HUWE1, and UBR4 poly-ubiquitinate A3H-I in cells, likely independently of one other (Fig. 2G, H). To validate these results in a cell-free system, we performed in vitro ubiquitination assays using purified proteins and either A3H-I, A3H-II or the RNA-free A3H-II-RBM mutant as substrates. Consistent with our cell-based data, UBR5 and HUWE1 each directly poly-ubiquitinated A3H-II-RBM and A3H-I (Fig. 5C, D). Both enzymes polyubiquitinated the RNA-free A3H-II-RBM mutant most strongly, followed by RNA-bound A3H-I, with A3H-II showing the lowest modification levels (Fig. 5C, D). All three A3 substrates were efficiently mono-ubiquitinated by the E2 conjugating enzyme UBE2A (a.k.a. RAD6A) in the absence of its E3 ligase UBR4 (Fig. 5E). Addition of UBR4 efficiently extended the mono-ubiquitin into poly-ubiquitin chains (Fig. 5E; compare samples 4–6 with 7–9). These data suggest that UBR5 and HUWE1 indeed each poly-ubiquitinate A3H directly. In contrast, UBR4 requires prior substrate mono-ubiquitination by its specific E2 conjugating enzyme UBE2A. Consistent with recent structural and biochemical findings that have indicated that UBR4 synthesizes poly-ubiquitin chains on mono-ubiquitinated or partially ubiquitinated substrates (i.e., and E4 ligase function)^63–66^, UBR4 also seemed to adopt an E4 ubiquitin chain-extending function in our assays. However, UBR4-dependent chain extension is not haplotype-specific, suggesting that UBR4 can likely extend any pre-formed chains on A3H.

Cell-based assays had shown that while A3H-I is turned-over by the proteasome, engineered RNA-binding mutants were substantially more unstable (Fig. 4A, B). Since A3H-I purified from *E. coli* bound to substantial amounts of bacterial RNA, we speculated that it might bind RNA in cells. However, the equilibrium may be shifted to a larger unbound fraction of A3H-I in cells compared to A3H-II. Based on the SEC chromatograms, we predicted that the vast majority of purified WT A3H protein molecules were RNA-bound in our preparations (Supplementary Fig. 7B), possibly not fully mirroring in-cell equilibrium conditions. Consistent with this notion, RNA-bound A3H-I and A3H-II were substantially less ubiquitinated in vitro by each of the three E3 ligases compared to A3H-II-RBM.

We hypothesized that this difference might stem from the co-purified RNA shielding the A3H substrates from E3 ligase engagement and predicted that RNA removal during the reaction would render them susceptible to ubiquitination. To test this, RNase A was added to the ubiquitination reaction mixtures. Under these conditions, both A3H-I and A3H-II were ubiquitinated by UBR5, HUWE1, and UBR4 to a similar extent as the A3H-II RBM (Fig. 5F–H). In contrast, ubiquitination of the RNA-free A3H-II-RBM mutant was independent of RNase A addition. From these data, we concluded that RNA indeed prevents either ubiquitination or E3 ligase recognition of A3H.

Given that ubiquitination of multiple lysine residues compared to lysine residues located in the RNA binding region alone contribute to A3H turnover (Fig. 1G, H), we concluded that it is rather the ability of E3s to recognize and bind to A3H in a non-RNA-bound state. The flexibility of these large E3 ligases would allow ubiquitination of multiple surface lysine residues. To experimentally test whether RNA binding indeed prevents A3H from being recognized by UBR5 and HUWE1 as substrates, we next performed in vitro interaction studies. We employed Analytical Ultracentrifugation to assess UBR5 and A3H protein-protein interaction (Fig. 5I, J, Supplementary Fig. 7C).

Consistent with our cell-based data, RNA-bound recombinant A3H-I and A3H-II did not co-fractionate with UBR5 (Fig. 5I). However, the A3H-II RNA-binding mutant (Fig. 5I, bottom panel) or addition of RNase A to the RNA-bound A3H-I and A3H-II preparation (Fig. 5J) caused UBR5 to co-fractionate. These results indicate that RNA binding indeed prevents A3H from being bound and recognized as a substrate by UBR5.

Likewise, we applied analytical size exclusion chromatography of HUWE1 to assess its interaction with A3H (Fig. 5K). Similar to the results with UBR5, only the RNA-binding mutant showed a strong interaction with HUWE1 (Fig. 5K, bottom panel).

Our cell-based assays indicated that, like A3H-I, A3B was dependent on UBR5, HUWE1, and UBR4 for its turnover, whereas A3A was not (Fig. 2G, H, Supplementary Fig. 3C). Unlike A3H A3B is a dual-domain A3 protein. To test whether either one of the A3B domains or both were direct substrates for these E3 ligases, we purified both the A3B CD1 and CD2 domains, and for comparison A3A (Supplementary Fig. 7E, F). A3A and both A3B domains purified without any RNA (Supplementary Fig. 7E, F). We were unable to purify sufficient full-length A3B for in vitro assays.

Comparable to the A3H-II RNA binding mutant, both the A3B CD1 and CD2 domains were efficiently poly-ubiquitinated by UBR5 (Supplementary Fig. 8A, B), HUWE1 (Supplementary Fig. 8C, D), and UBR4 (Supplementary Fig. 8E, F). In agreement with cell-based data, A3A was substantially less ubiquitinated under these conditions (Supplementary Fig. 8A–F; 2–3-fold). To test whether A3B is directly recognized as a substrate, similar to RNA-free A3H, we performed analytical ultracentrifugation assays with UBR5, which showed that both the A3B CD1 and CD2 domains are directly bound by UBR5, whereas A3A was not (Supplementary Fig. 8G).

Together, these data are consistent with a model in which RNA binding prevents A3H from binding and ubiquitination by the E3 ligases. In the absence of RNA binding, UBR5 and HUWE1 independently poly-ubiquitinate A3B domains and A3H. Similarly, in the absence of RNA binding, mono-ubiquitination by UBE2A was enhanced, which further increased UBR4 poly-ubiquitin chain extension (E4 ligase) activity, extending poly-ubiquitin chains on pre-formed (mono)-ubiquitin marks (Fig. 5H). Based on these findings and published data^67^, it seems likely that UBR4 can additionally amplify poly-ubiquitination in cellular contexts, by targeting substrates partially ubiquitinated by UBR5 or HUWE1, or factors that were not identified in our screens.

It should be noted that changes in A3H-RNA association could affect substrate folding and solubility, which in turn could affect substrate selection. In this context, our analytical ultra-centrifugation assays exclude that RNA-free A3s are aggregated after in vitro assays. Aggregation would have resulted in higher molecular weight formation and the substrate aggregates running at the bottom of the sucrose gradients, which they did not (Fig. 5I–K, Supplementary Fig. 7C, D, Supplementary Fig. 8G). Although we are of the opinion that our collective data is most consistent with the E3 ligases targeting unoccupied nucleic acid binding domains in the A3 substrates, our data cannot exclude that subtle changes in protein folding upon RNA removal influenced their recognitions as substrates.

### E3 ligase loss or mutation increases APOBEC signature mutations

Single Base Substitution (SBS) signature mutations are specific patterns of genetic mutations, categorized based on their types and surrounding sequence context. Unbiased computational analysis has identified over 67 different SBS patterns; among these are two highly APOBEC3-specific signatures (SBS2, SBS13)^24,30^.

**Fig. 6:**
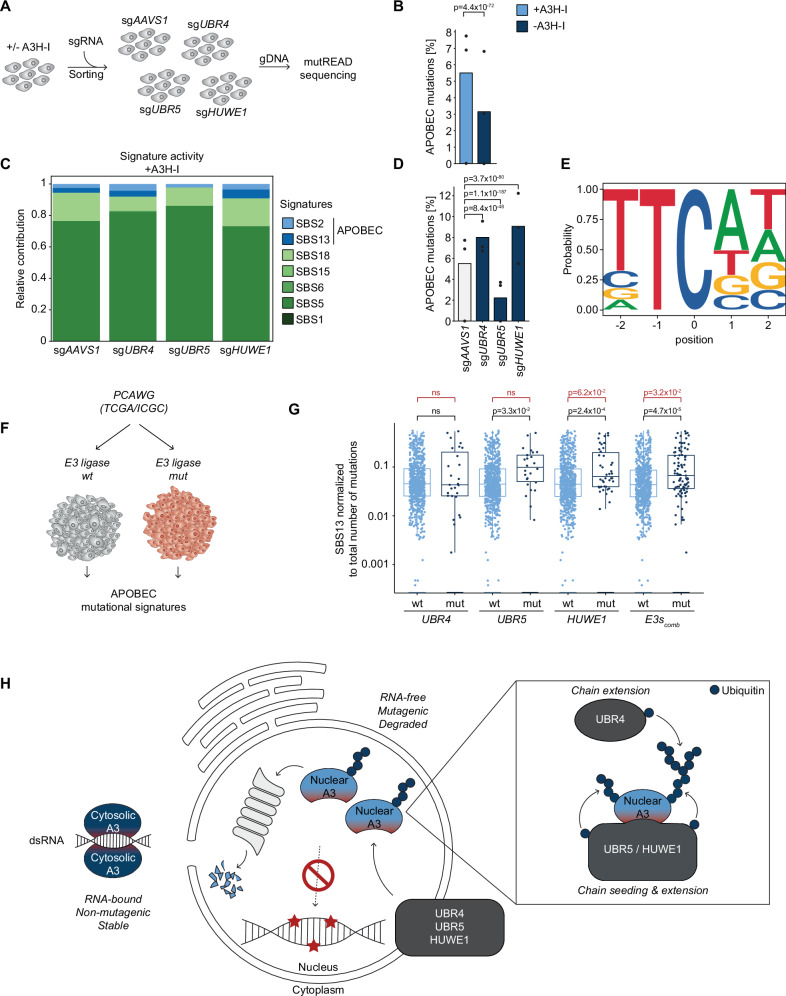
E3 ligase loss or mutation increases APOBEC signature mutations. **A** Schematic of mutREAD sequencing to detect APOBEC signature mutations. UNG2-deficient RKO cells expressing DOX-inducible Cas9 and with or without mCherry-P2A-3xHA-A3H-I overexpression were transduced with sgRNAs targeting *UBR4*, *UBR5*, or *HUWE1*. Following sorting for sgRNA-positive cells, gene editing was induced with DOX for up to 10 days, after which genomic DNA was isolated for mutREAD sequencing. **B** Fraction of APOBEC signature related mutations over all identified mutations in mutREAD sequencing control samples (n = 3 biological replicates, two-sided Fisher’s exact test, no adjustments for multiple comparisons, test statistic = Odds Ratio = 0.5588, p-value = 4.41 × 10^−72^, CI^low^ = 0.5239, CI^high^ = 0.5960). **C** Best-subset signature refitting of samples expressing exogenous A3H-I, averaged per genotype, using APOBEC-associated and colon carcinoma signatures. Each bar represents the mean of three technical replicates scaled to one. **D** Fraction of APOBEC-related mutations in samples with exogenous A3H-I expression (n = 3 biological replicates, two-sided Fisher’s exact test with Bonferroni correction; odds ratio, 95% confidence intervals, and exact p-values shown). **E** Pentanucleotide context preference of APOBEC mutations in control samples expressing A3H-I. **F** Schematic of mutational signature analysis using PCAWG data. **G** TCGA and ICGC cancer samples were grouped by wild-type or mutated status of *UBR4*, *UBR5*, and *HUWE1*. Mutational signatures were normalized to the total number of mutations in each sample. The level of SBS13 APOBEC signature was compared between the groups. “E3s_comb_” are all samples in which at least one of the E3s is mutated. Wilcoxon rank-sum test, two-sided, ns: p > 0.05, n = 2703 samples). Box plots show median, interquartile range, and whiskers to 1.5× IQR; y-axis is log10-transformed. **H** Model: lack of RNA-binding determines A3 nuclear localization and simultaneously targeting by th eE3 ligases, ensuring low nuclear A3 levels.

As ablation of *UBR4*, *UBR5*, and *HUWE1* elevated levels of cancer-associated A3B and A3H-I (Fig. 2E–H), we hypothesized that this would increase A3-dependent mutagenesis (Fig. 6A). To test this hypothesis in an experimentally feasible time frame, we generated an RKO cell line with and without exogenous A3H-I-driven mutagenic activity (Supplementary Fig. 9A) and analyzed APOBEC signature mutations. Cellular gDNA was isolated from these samples, and mutREAD sequencing was performed to determine changes in SBS signature mutations in the gDNA of the analyzed cell pools in an unbiased manner (Fig. 6A)^24,30,68^. This analysis yields a relative distribution of all SBS signature mutations identified in the gDNA of the different genotypes.

RKO cells have no detectable endogenous *A3A* or *A3H* mRNA expression, yet express detectable A3B mRNA and protein levels (Fig. 2G). Therefore, these cells would predominantly accumulate APOBEC mutations driven by the exogenous A3H-I, and potentially by endogenous A3B. Indeed, exogenous A3H-I expression increased APOBEC3-specific signature mutations (Fig. 6B).

Cells with or without exogenous A3H-I were transduced with sgRNAs individually targeting *UBR4*, *UBR5*, or *HUWE1*, sorted for sgRNA-positive cells, after which gene-editing was induced with DOX (Fig. 6A). We measured E3 ligase ablation and upregulation of A3H-I/A3B in our samples by WB (Supplementary Fig. 9B), showing knock-out of each E3 ligase and increased A3B/A3H-I protein levels in these samples.

In *AAVS1*-targeted control cells, two major SBS signatures were identified (Fig. 6C, Supplementary Fig. 9C; SBS5/18; signatures commonly accumulating in experimental systems). In cells without exogenous A3H-I (in which likely most APOBEC-dependent mutagenesis is mediated by A3B given the low mRNA levels of all other A3s in these cells) (Fig.6B), *UBR5* knock-out significantly increased APOBEC3 mutational signatures (Supplementary Fig. 9C–G) in a TTCAT context (Supplementary Fig. 9H). In addition, knock-out of *HUWE1* or *UBR4* also increased specific A3 signature mutations in the presence of elevated levels of exogenous A3H-I (Fig. 6C, D, Supplementary Fig. 9I–K), with an enrichment of mutations occurring in a TTCAT context (Fig. 6E).

Unexpectedly, the loss of UBR5 did not increase APOBEC signature mutations in cells expressing exogenous A3H-I (Fig. 6C, D, Supplementary Fig. 9I–K). The underlying reasons remain to be clarified in future studies. One plausible explanation, however, relates to the known role of UBR5 in the DNA damage response^69–72^. In cells lacking A3H-I, UBR5 knockout may have not only elevated endogenous A3B protein levels but also sensitized the cells to DNA damage, thereby enhancing the detection of APOBEC-associated mutations. In contrast, in A3H-I–expressing cells, the mutational burden from exogenous A3H-I may have already been sufficient to reveal stabilizing effects of UBR4 and HUWE1 knockout within the experimental timeframe. In this context, the heightened sensitivity of UBR5-deficient cells could have led to their selective loss due to excessive mutational toxicity.

Taken together, our data showed that under conditions in which degradation of A3 proteins is compromised, A3-driven signature mutations increase, resulting in an elevated mutational burden on the gDNA.

Lastly, we asked whether our cell-based findings would translate to a human setting. To this end, we tested whether mutation—and thus possible loss of function - of the identified E3 ligases, correlated with a specific increase in APOBEC-signature burden in cancer patient samples. For this purpose, cancer whole-genome sequence samples from the PCAWG study^30,73^ were sorted into bins, in which the gDNA sequence for a particular E3 ligase was either WT (wt) or had at least one SNV or InDel outside of intronic regions (mut) (Fig. 6F). In addition, samples in which any of the three E3 ligase genes (*UBR4*, *UBR5*, *HUWE1*) was mutated (E3s_comb_) were compared to all remaining samples.

Subsequently, SBS mutational signatures were retrieved, and the binned groups of each E3 ligase gene compared in a pairwise fashion. To account for a potential bias in the total mutational burden in the mutant groups of *UBR4*, *UBR5*, *HUWE1*, and unrelated E3 ligases (Supplementary Fig. 10A), mutational counts were normalized to the total number of mutations in each individual sample. This enabled direct comparison of relative SBS signature distributions between sample groups.

In line with our cell-based experiments, *UBR5-* and *HUWE1*-mutated cancer samples had significantly more APOBEC signature mutations (SBS2/SBS13) compared to their respective WT control groups (Fig. 6G and Supplementary Fig. 10B). In contrast to our cell-based data, there was no significant difference in APOBEC signature mutations between wild-type and *UBR4*-mutant samples (Fig. 6G and Supplementary Fig. 10B).

The effect in *UBR5*- and *HUWE1*-mutant samples was specific for APOBEC mutational signatures, as mutations in unrelated E3 ligases did not correlate with significant changes in APOBEC signature mutations (Supplementary Fig. 10C, D). In addition, mutations in *UBR5* or *HUWE1* did not correlate with an increase in other abundant, non-APOBEC signatures (Supplementary Fig. 6E–G; SBS1/SBS5/SBS18). In fact, the frequencies of these non-APOBEC signatures were predominantly decreased in mutant samples (Supplementary Fig. 10E, F), which likely stems from normalization to total mutation counts (Supplementary Fig. 10A). This indicates that -as one would expect- there is a bias toward greater numbers of total mutations in mutated samples, yet a significantly higher fraction of APOBEC signatures specifically occur in *UBR5* and *HUWE1* mutant cancers (Fig. 6G and Supplementary Fig. 10B). In agreement with our cell-based data, this suggests that *UBR5* and *HUWE1* are important for curtailing the levels of specific APOBEC proteins in human cancers thereby limiting APOBEC-driven mutagenesis.

Collectively, this study identifies a hitherto unknown framework of cellular guardians that keeps steady-state levels of nuclear A3 proteins low. Our data supports a model in which the core of this network consists of UBR5 and HUWE1, E3 ligases that directly recognize A3B and A3H-I and mark them for degradation (Fig. 6H). UBR4 may play an indirect, amplifying role through its E4 ubiquitin chain extension activity.

UBR5 and HUWE1 specifically engage A3 proteins in their non-RNA bound states, possibly through unoccupied RNA-binding surfaces. Since RNA binding is important for A3 cytosolic retention, this framework ensures (i) specific targeting of nuclear A3 proteins that pose a risk to our genome, while (ii) leaving cytosolic A3 family members available as antiviral restriction factors. Since broad mutational landscapes in cancers enable escape from therapeutic interventions, our findings enable future studies into the identified E3 ligases as possible diagnostic or therapeutic targets.

## Discussion

High A3 levels have been identified in many cancers, and are correlated with higher A3-induced mutational rates (reviewed in ref. ^74^), with A3A, A3B, and A3H-I being the major sources of these signatures^18,28,34,35^. The prevalence of APOBEC signatures, found in >50% of all cancers and across cancer types^30^, underpins the critical importance of understanding how human cells restrict APOBEC-induced mutagenesis of their own genomes. Thus far, studies have focused on the differential transcriptional regulation of cancer-associated A3s to explain the high prevalence of APOBEC signatures in tumors. However, other major modes of A3 regulation may play a critical role in cancer mutagenesis.

Here, we identified a post-translational regulatory mechanism regulating cellular A3B and A3H-I protein abundance, two major APOBEC signature drivers^18,28,34,35^. The mechanisms we uncovered limit APOBEC-induced mutagenesis in cells. We show that the loss of the central E3 ligases, UBR4, UBR5, and HUWE1, stabilizes A3B and A3H-I proteins, resulting in their accumulation and eventually increasing APOBEC-related mutational burden. Our findings reveal a previously unknown layer of regulation acting to limit cellular A3 protein levels. In the absence of these guardian factors, A3s may broaden the mutational landscape in late-stage cancer and affect the development of therapy resistance^33,75–78^.

### RNA binding stabilizes A3 proteins

RNA binding mediates important A3 regulatory functions, bridging A3s to form high-molecular-weight complexes in the cytoplasm and inhibiting catalytic activity^36,54,79–84^. In an antiviral context, this leads to inhibition of A3 deaminase activity by the viral RNA until it is released during reverse transcription. However, nuclear A3 accumulation is often the result of diminished cytosolic RNA binding^41^, and comes at the risk of accumulation of an active enzyme with access to the host genome. This requires a cellular framework to ensure high levels of cytosolic antiviral A3 variants, while limiting the concentrations of nuclear, active A3 variants in order to preserve genome integrity.

Our data provide a mechanistic explanation of how such selectivity is achieved: E3 ligases target the A3 RNA-binding surface, a region that also determines its subcellular localization based on whether RNA is bound. This ensures that variants that bind less RNA and are more nuclear, simultaneously become more accessible E3 ligase substrates targeted for degradation. Our in vitro experiments showed that RNA binding plays a major mechanistic role in determining whether UBR5 and HUWE1 can engage A3 proteins as substrates (Fig. 5). Cell-based microscopy and fractionation experiments showed that A3H-I and RNA-binding mutants are predominantly nuclear and degraded in the nucleus (Fig. 4). This degradation may be mediated by the large fraction of cellular proteasomes residing in the nucleus^46^. However, it remains to be determined where E3-substrate interaction and ubiquitination take place in cells. Localization of the E3 ligases varies in different cell types, and previous analysis places all three E3 ligases in the nucleus and cytosol^85–92^.

Previous work has shown that A3H-II has strong RNA-binding activity^36,54,79–84^. However, since the R105G mutation lies outside of the RNA-binding interface, it was unclear whether A3H-I has differential RNA-binding ability, and whether this may underlie the phenotypic changes in localization and stability. Our cell-based data consistently showed intermediate nuclear localization and instability phenotypes for A3H-I, in between A3H-II and designed RNA-binding mutants. Moreover, SEC analysis of recombinant A3H-I revealed that it still co-purified with substantial amounts of bound RNA, albeit less than its A3H-II counterpart. Moreover, while A3H-II purified in part as RNA-bound dimers, A3H-I was exclusively obtained as an RNA-bound monomer.

In line with the findings of a recent interaction study^50^, our cell-based TurboID proteomics showed that A3H-II and A3H-I share many interactors related to RNA processes, albeit fewer for A3H-I. This further supports a model in which A3H-I is still bound by RNA in cells, but to a lesser extent than A3H-II. Together, our findings indicate that A3H-I is likely a partial RNA-binding mutant, for which the equilibrium in cells is shifted towards a more unbound state. As a consequence, it may be less amenable to forming high molecular weight RNA/A3 RNP complexes, as previously described^36,54,79–84^, allowing passive diffusion into the nucleus^44^. The R105G mutation and structural changes in its β-sheet may affect the proper positioning of the distal RNA-binding domain. Nevertheless, we cannot rule out additional effects on protein-protein interactions required for high molecular weight complex formation as seen for A3H-II^36,54,79–84^. At least two nuclear and unstable A3H haplotypes arose independently in evolution^10,12,13,42^, suggesting either negative counter-selection of cytosolic variants, or positive selection for nuclear A3 enzymes. In line with this latter notion, various A3 members have been reported to counter infections of unrelated nuclear viruses and transposable elements with DNA genomes or replication intermediates^93–99^. It is, therefore, essential to balance the required nuclear antiviral activity with preventing genomic hypermutation. The identified E3 ligase machinery ensures this balance by only recognizing nuclear A3s that are not engaged with RNA.

### Substrate preference and cooperativity of UBR4, UBR5, and HUWE1

The three identified A3-targeting E3 ligases, UBR5, HUWE1, and UBR4, have previously been linked to the turnover of a diverse array of substrates, thereby exerting control over numerous cellular processes^67,85,86,88,100–118^. This suggests that these E3 ligases possess the ability to identify multiple substrate classes based on broad biochemical or biophysical characteristics.

Supporting this idea, recent structural studies of HUWE1 have revealed three distinct substrate-binding domains^119,120^, facilitating the recognition and ubiquitination of unbound nucleic acid binding proteins as well as ubiquitinated/PARylated substrates. Similarly, UBR5 was recently shown to bind and ubiquitinate unengaged transcription factors^100^, and has the ability to function as a ubiquitin chain elongating E4^103,104^. In combination with our findings in this study, these results indicate that HUWE1 and UBR5 are both important players in recognizing and degrading unengaged DNA- and RNA-binding proteins.

Although comprehensive structural information is currently unavailable for UBR4, its involvement in ubiquitinating aggregation-prone nascent polypeptides during proteotoxic stress^107^, diverse mitochondrial proteins^100,108^, and ER-associated degradation substrates^109^ identified in independent screens suggests that this large E3 ligase may also possess the capability to recognize various substrate classes, potentially contingent upon its interaction with different partners. While UBR4 may define substrate selection in other cellular contexts, our data show that in the context of A3 degradation, it likely plays a role as a chain extending E4 ligase. In line with this, UBR4 was neither enriched in our TurboID experiments, nor identified by co-IP (Fig. 3), pointing towards a more distal engagement. However, it could be that UBR4 is specifically recruited to UBR5- or HUWE1-containing complexes in cells to amplify their ubiquitination, and, thereby, proteasomal degradation. In agreement with previous reports^67,121^, our cell-based (Fig. 2G) and in vitro (Fig. 5) data suggest that UBR4, UBR5, and HUWE1 have redundant functions and cooperate to assemble ubiquitin chains on their substrates.

Our cell-based data indicated a non-epistatic/functionally redundant role for UBR4 with the other two ligases, whereas our in vitro data may indicate that it could contribute in a poly-ubiquitin chain extending E4 ligases capacity, consistent with recent structural and biochemical studies^63–66^. These two possibilities are not mutually exclusive and could both occur in a cellular context. This could suggest that additional E3 ligases contribute to A3 degradation, with which UBR4 cooperates for chain-extension.

### Maintenance of genome integrity

Prior studies have indicated that A3A and A3B are responsible for a substantial part of APOBEC signatures in cancer^28,34^. The presence of the distinct A3H-I haplotype was linked to increased APOBEC signatures in breast and lung cancers^18,32^. However, additional evidence for the contribution of A3H-I to APOBEC3 signatures in other cancers is sparse and little research focuses on A3H-I. We show that APOBEC signatures accumulate in cells in which A3H-I is exogenously expressed, and that impairment of A3H-I and A3B degradation mediated by UBR4, UBR5 and HUWE1 results in an increase in their protein levels, paralleled by an increase in APOBEC signature mutations (Fig. 6). In agreement, we found a significant correlation between *UBR5* or *HUWE1* mutations in human cancer genomes and increased APOBEC signature mutations in these samples. Therefore, analysis of A3 protein levels and/or the mutational status of UBR5 and HUWE1 may prove helpful as a future diagnostic tool that acts as a proxy for the tumor mutational landscape and indicates the likelihood of developing treatment-resistance.

Several proteasome inhibitors are used as therapy for treating multiple myeloma and mantle cell lymphoma, yet relapses and acquired resistance are frequent^122^. The role of APOBEC mutagenesis in cancer therapy and evolution has been suggested to be a double-edged sword. Depending on the level of APOBEC mutagenesis, it could either contribute to greater treatment effectiveness by driving error-catastrophe and synthetic-lethality in cancer cells, while on the other hand it could have detrimental effects by broadening the mutational landscape in tumors, thereby increasing the frequencies of therapeutic resistance^123^. With our finding that proteasomal degradation plays an important role in regulating cancer-associated APOBEC3 protein levels, the application of proteasome inhibitors in cancer therapy and the resulting increase in cellular A3 levels should be assessed accordingly.

### Future directions

Our data present the importance of a previously underrated RNA-dependent regulatory mechanism of APOBEC3 protein activity and localization through proteasomal degradation controlling levels of nuclear APOBEC3s. While A3A is even more unstable than A3B and A3H-I, it is not ubiquitinated (Fig. 1A and Supplementary Fig. 1H), indicating that cancer-associated A3A protein levels are controlled by a different cellular mechanism. The lack of ubiquitination could suggest A3A is degraded in a ubiquitin-independent manner. Moreover, previous work has shown that A3A transcription is influenced by proteasome inhibition^124^, possibly contributing to the observed increase in A3A protein levels.

Nevertheless, it raises the question of why A3A is not a UBR5/HUWE1 substrate, in contrast to A3B and A3H-I. A3A differs from A3B and A3H-I as it does not form large multimeric complexes^125–127^. The protein sequence of single-domain A3A resembles the C-terminal domain (CTD) of A3B and A3G. Even though the CTD is also implicated in RNA-binding, the critical RNA-binding residues comprising bulky hydrophobic and positively charged amino acids in loop7 and the α6-helix are located in the inactive N-terminal domain (NTD) of dual-domain A3s^60,128^. Since the sequence of A3H is similar to the NTD of A3B, we speculate that A3A lacks critical structural or biophysical properties/features/motifs and can therefore not be recognized by the E3 ligases identified in this study. Thus, future studies will be necessary to identify mechanisms through which A3A protein levels are regulated by proteasomal degradation.

In sum, our current data identify a critical mechanism by which A3B and A3H-I are linked to APOBEC mutation signatures, making these cancer-associated A3s a strong starting point for future development of therapeutic and diagnostic tools.

## Methods

### Reagents

All reagents and their sources, including chemicals, antibodies, oligonucleotides, plasmids, recombinant proteins, and software, are available in the Supplementary Methods. Reagents generated in this study are available upon request to the corresponding author.

### Vectors

The lentiviral human ubiquitin-focused sgRNA library consists of 6 sgRNAs per gene for ubiquitin-proteasome system- and autophagy-related genes, and has been described^129^. Lentiviral vectors expressing single or dual sgRNAs from a U6 promoter and eBFP2 or iRFP from a PGK promoter have been described^46^. sgRNA CDSs were cloned in pLentiv2-U6-PGK-iRFP670-P2A-Neo^46^ to perform knock-outs in RKO cell lines. The Dual-A3H-reporter (pLX303-SFFV-MYC-mCherry-A3H-II-P2A-OLLAS-EGFP-A3H-I) was constructed by cloning the ORF of human A3H-I or A3H-II into a modified pLX303 vector^48,130^. cDNAs encoding A3H-I, A3H-II, A3H-I-G105R, A3H-II-R105-G, A3H-II-W115A, A3H-II-R175/176E, A3H-II-E56A-W115A-R175/176E, A3H-I-K-mutants, A3A, A3C, A3D, A3F, A3G, A3G-RNA-binding mutants were synthesized by Twist Bioscience, or generated by fusion PCR and cloned into a modified pLX303 vector for mammalian expression, or into a modified pET47 containing a decahistidine (10×His) tag followed by a Maltose binding protein (MBP) tag and a 3C site vector for bacterial expression. The plasmids, oligos, and sgRNAs used in this study are listed in the Supplementary Methods.

### Cell culture

Unless otherwise indicated, experiments in this study were reproduced at least twice in independent experiments. All cell lines used in this study were maintained at 37 °C with 5% CO2 in a humidified incubator, routinely tested for mycoplasma contamination, and authenticated by STR analysis. Human RKO (sex unspecified; American Type Culture Collection (ATCC) cat. no. CRL-2577, RRID:CVCL_0504) and THP-1 cells (male; ATCC cat no. TIB-202, RRID:CVCL_0006) were cultured in RPMI 1640 (Gibco) supplemented with 10% FBS (Sigma-Aldrich), L-glutamine (4 mM, Gibco), sodium pyruvate (1 mM, Sigma-Aldrich) and penicillin/streptomycin (100 U/ml/100 μg/ml, Sigma-Aldrich). HeLa cells (female; ATCC cat. no. CCL-2, RRID:CVCL_0030), Lenti-293T lentiviral packaging cells (female, Clontech, cat. No. 632180), and HEK-293T cells (female; ATCC cat. No. CRL-3216, RRID: RRID:CVCL_0063) were cultured in Dulbecco’s modified Eagle’s medium (DMEM; Sigma-Aldrich) supplemented with 10% FBS and penicillin/streptomycin (Sigma-Aldrich). All cell lines used in this study are listed in the Supplementary Methods.

### Generation of clonal iCas9 cell lines

THP-1-DOX-Cas9-P2A-GFP cells were generated by transducing THP-1 cells with the pRRL-TRE3G-Cas9-P2A-GFP-PGK-IRES-rtTA3 lentiviral vector^48^. Cas9 expression was induced with 500 ng/ml of Doxycycline hyclate (DOX, Sigma-Aldrich), and single cells were sorted by FACS into 96-well plates using a FACSAria III cell sorter (BD Biosciences) to obtain single-cell-derived clones. To generate the genetic screening cell line (Dual-A3H-reporter), RKO-DOX-Cas9-P2A-BFP cells^129^ were transduced with pLX303-SFFV-MYC-mCherry-A3H-II-P2A-OLLAS-EGFP-A3H-I, and mCherry^+^/GFP^+^ double-positive cells were sorted by FACS into 96-well plates using a FACSAria III cell sorter (BD Biosciences). The mutREAD cell line was generated by co-transducing pLX303-SFFV-MYC-mCherry-P2A-3xHA-A3H-I and DualCRISPR-hU6-sg*UNG2-*mU6-sg*UNG2*-Thy1.1-P2A-Neo into RKO-DOX-Cas9-P2A-GFP^46^. Following Cas9 induction with 200 ng/ml DOX for 6 days, live cells were immunostained for the Thy1.1 surface marker. In brief, cells were washed with PBS and incubated for 15 min at 4 °C in Human BD Fc Block (BD Biosciences) to inhibit non-specific antibody binding. Cells were then stained with APC anti-rat CD90/mouse CD90.1 (Thy-1.1) Antibody (BioLegend, 1:260) for 20 min. at 4 °C. Following two washes, mCherry^+^/Thy1.1^+^ single cells were sorted into 96-well plates. *UNG2* homozygous knock-out was confirmed by PCR amplification, TA-cloning, and Sanger sequencing of the targeted *UNG2* locus. Polyclonal RKO cell lines were obtained by transducing the parental RKO-DOX-Cas9-P2A-BFP or RKO-DOX-Cas9-P2A-GFP cells with the respective lentiviral expression plasmids listed in the Supplementary Methods.

### Transfections

Transfections for analysis by WB were performed by mixing DNA and polyethylenimine (PEI, Polysciences) in a 1:3 ratio (w/w) in DMEM (Sigma-Aldrich) without supplements. Transfection was performed using 1000 ng of total DNA per well. The day before transfection, 2 × 10^5^ HEK-293T cells were seeded in 6-well clusters in fully supplemented DMEM media. 36 h. after transfection cells were harvested, washed with ice cold PBS and stored at −80 °C until further processing.

### Lentivirus production and transduction

Lenti-293T lentiviral packaging cells were transfected with DNA mixes containing lentiviral transfer plasmids, pCRV1-Gag-Pol^131^ and pHCMV-VSV-G^132^ using polyethylenimine (PEI, Polysciences) in a 1:3 μg DNA/µl PEI ratio in non-supplemented DMEM. Virus-containing supernatants were clarified of cellular debris by filtration through a 0.45 μm filter. Virus-like particles were directly used after harvesting or stored at −80 °C. Target cells were transduced in the presence of 5 μg/ml polybrene (Sigma-Aldrich).

### FACS-based CRISPR–Cas9 screens

Lentivirus-like particles were used to transduce RKO-DOX-Cas9-mCherry-A3H-II-P2A-EGFP-A3H-I cells (Dual-A3H-reporter) at a multiplicity of infection (MOI) of less than 0.2 TU/cell, and 500 to 1000-fold library representation. The percentage of library-positive cells was determined after 3 days of transduction by immunostaining for the Thy1.1 surface marker and subsequent flow cytometric analysis. RKO cells with integrated lentiviral vectors were selected with G418 (1 mg/ml, Sigma-Aldrich) for 5 days. After G418 selection, Cas9 genome editing was induced with DOX (350 ng/ml, Sigma-Aldrich), and after 3 days and 6 days, cells were sorted by FACS. Therefore, cells were harvested, washed with PBS, resuspended in supplemented RPMI-1640, and sorted using the FACSAria III cell sorter operated by BD FACSDiva software (v8.0). RKO cells were gated for non-debris, singlets, BFP-positive (Cas9 expression), EGFP-positive, mCherry-positive, and 1–2% of cells with the lowest or highest EGFP or mCherry signals were sorted into PBS. At least 1 × 10^6^ cells were collected for each population at each time point in three independent experiments. Sorted samples were re-analyzed for purity, pelleted, and stored at −80 °C until further processing. Additionally, 10 million cells from an unsorted reference sample corresponding to 1000-fold library representation were collected on each sorting day and stored at −80 °C until further processing.

### Next-generation sequencing library preparation

NGS libraries of sorted and unsorted control samples were processed as previously described^46^. In brief, isolated genomic DNA was subjected to a two-step PCR. The first PCR amplified the integrated sgRNA cassettes, whereas the second PCR introduced the Illumina adapters. Purified PCR products’ size distribution and concentrations were measured using a fragment analyzer (Advanced Analytical Technologies). Equimolar ratios of the obtained libraries were pooled and sequenced on a HiSeq 2500 platform (Illumina). Primers used for library amplification are listed in the Supplementary Methods.

### Analysis of pooled CRISPR screens

The analysis of the genetic screens was carried out as previously described^46^. Three biological replicates of each sort were included in the analysis. In brief, sgRNAs enriched on day 3 and day 6 (post-Cas9 induction) sorted samples were compared to the matching unsorted control populations harvested on the same days using MAGeCK 0.5.9.3^133^. Hits were selected based on log_2_ fold-change and p-value and grouped by functional categories. To exclude unspecific hits, we selected genes enriched in EGFP-A3H-I^high^ cell populations on day 3 with a log_2_ fold-change >0.45 and adj. p-value < 0.05, which were neither enriched in mCherry^high^ on day 3 (log_2_ fold-change >0.45, p-value < 0.05) nor in GFP^low^ on day 3 (log_2_ fold-change >0.45, p-value < 0.05). Similarly, genes enriched in EGFP-A3H-I^high^ cell populations on day 6 with a log_2_ fold-change >0.6 and adj. p-value < 0.05, which were neither enriched in mCherry^high^ on day 3 (log_2_ fold-change >0.45, p-value < 0.05) or day 6 (log_2_ fold-change >0.6, p-value < 0.05) nor in GFP^low^ on day 6 (log_2_ fold-change >0.6, p-value < 0.05) were selected.

### Protein half-life determination

To estimate A3H-I or A3H-II protein half-lives, cells were treated with 200 μg/ml of cycloheximide (CHX, Sigma-Aldrich). At the indicated time points, total protein extracts were generated using 2x Disruption buffer (1.05 M Urea, 0.667 M β-Mercaptoethanol and 0.7% SDS) or RIPA buffer supplemented with 1% SDS and Benzonase (50 mM Tris HCl (pH 7.4), 150 mM NaCl, 1% NP-40, 0.5% Sodium Deoxycholate, 1 mM EDTA, 1% SDS, 1 mM PMSF (Sigma-Aldrich), Benzonase (25 U/ml, Merck) and 1X cOmplete Protease Inhibitor Cocktail (Roche), analyzed by WB, quantified and normalized as indicated. Single exponential decay curves were plotted using GraphPad Prism (v9), from which protein half-lives were calculated.

### Immunofluorescence confocal microscopy

250,000 RKO cells stably expressing OLLAS-A3H-I or OLLAS-A3H-II (RKO-MYC-mCherry-P2A-OLLAS-A3H-I/II), EGFP-A3H-I/II/I-G105R/-II-R105G/II-W115A/II-R175/176E or EGFP-tagged A3G WT and A3G RNA-binding mutants (6 M (F126Y, W127S, K180A, I183A, L184A, I187A), FWKL (F126Y, W127S, K180S, L184S) and Y181A/Y182A) were seeded onto coverslips. After 48 hours, cells were fixed with 4% paraformaldehyde (PFA) for 15 min. In case of antibody staining, cells were permeabilized with 0.25% Triton X-100 in PBS for 5 min, followed by blocking of non-specific sites by incubation with 1% BSA for 30 min. at RT. Coverslips were incubated for 1 h. at RT with primary anti-A3H antibody (Novus Biologicals) 1:100 in 1% BSA, followed by incubation with 1:800 anti-rabbit IgG Alexa Fluor 488 (Abcam) secondary antibody, and incubation for 5 min with 0.4X Hoechst (Thermo Fisher Scientific) in PBS. The coverslips were mounted using ProLong Gold Antifade Mountant (Invitrogen). Images were collected using a Zeiss LSM 980 confocal microscope at 40X magnification for A3H samples and an Airyscan at 63X magnification for A3G samples.

### Subcellular fractionation

Subcellular fractionation was performed as previously described^134^. In brief, two million cells were washed in 1 ml PBS and lysed in 500 µl ice-cold REAP buffer (0.1% NP-40 in 1x PBS) supplemented with 1 mM PMSF (Sigma-Aldrich), 1X cOmplete Protease Inhibitor Cocktail (Roche), and Benzonase. 240 µl of the lysates were collected as whole cell fractions, the remaining lysate was centrifuged at 3000 × *g* for 60 s at 4 °C. 240 µl of supernatants were collected as cytosolic fractions, after which pellets were washed with 500 µl of REAP buffer, collected by centrifugation at 3000 × *g* for 60 s at 4 °C, and then resuspended in 240 µl of REAP buffer (nuclear fraction). All fractions were subsequently supplemented with 6x Laemmli sample buffer (62.5 mM Tris-HCl (pH 6.8), 5.8% Glycerol, 2% SDS, and 1.7% β-Mercaptoethanol), and boiled for 10 min. Equal volumes of fractions were loaded on a 12% SDS polyacrylamide gel.

### Western blot analysis

24-48 hours post seeding, cells were treated with various inhibitors (CHX (200 µg/ml), MG132 (10 µM), EPOX (10 µM), CQ (50 µM), BafA (400 nM), NH4Cl (20 mM), Leu (50 µM), Capzimin (10 µM), TAK-243 (0.5 µM), MLN4924 (20 µM), CB-873 (5 µM)) for the indicated time points or left untreated. Cells were lysed in Disruption buffer or RIPA lysis buffer supplemented with 1% SDS and Benzonase. Lysates were rotated for 30 min at 4 °C and then centrifuged at 18,500 x *g* for 10 min at 4 °C. Supernatants were transferred to new tubes, and protein concentrations were determined using the BCA Protein Assay Kit (Thermo Fisher Scientific). Between 20 and 50 μg of protein per sample was mixed with Laemmli sample buffer and boiled for 10 min. Proteins were loaded on 12% or 15% SDS polyacrylamide gels, based on the molecular weight of the protein of interest, or alternatively 3–8% NuPage gels (Invitrogen) to probe for high molecular weight proteins. Proteins were separated by SDS page using Tris-Glycine (25 mM Tris, 192 mM glycine, 0.1% SDS) or Tris-Acetate (2.5 mM Tricine, 2.5 mM Tris, 0.05% SDS) SDS running buffer, respectively. Proteins were blotted on PVDF or nitrocellulose membranes at 4 °C for 75 min at 300 mA in Towbin buffer (25 mM Tris, pH 8.3, 192 mM glycine and 20% ethanol). Membranes were blocked in 5% BSA in PBS-T for 1 h at RT, and subsequently incubated with primary antibodies diluted in 5% BSA overnight at 4 °C (ARP10 Antibody (Novus, 1:1000), Anti-APOBEC3B Antibody (Abcam, 1:1000), Anti-APOBEC3G (D9C6Z) Rabbit mAb (Cell Signaling Technologies, 1:1000), Anti-MYC antibody (Sigma-Aldrich, 1:5000), HA-Tag (C29F4) Rabbit mAb (Cell Signaling Technology, 1:1000), HA-Tag (6E2) Mouse mAb (Cell Signaling Technology, 1:1000), OLLAS Epitope Tag Antibody (L2) (Novus, 1:4000), Anti-Penta·His Antibody (Quiagen, 1:10.000), LC3B Antibody (Cell Signaling Technology, 1:1000), Ubiquitin (P4D1) (Santa Cruz Biotechnology, 1:1000), Anti-UBR4/p600 antibody (Abcam, 1:1000), Rabbit anti-EDD1 Antibody (Bethyl, 1:1000), Rabbit anti-Lasu1/Ureb1 Antibody (HUWE1) (Bethyl, 1:1000), Monoclonal Anti-α-Tubulin antibody produced in mouse (Sigma-Aldrich, 1:1000), Lamin A/C Antibody (E-1) (Santa Cruz Biotechnology, 1:1000), Monoclonal Anti-Vinculin antibody (Sigma-Aldrich, 1:1000), Anti-beta Actin antibody (HRP) (Abcam, 1:20000)). The next day, the membranes were washed three times for 5 min each with PBS-T and incubated with HRP-coupled secondary antibodies in 5% skimmed milk for 1 h at RT (Anti-rabbit IgG, HRP-linked Antibody (Cell Signaling Technology, 1:3500), Anti-mouse IgG, HRP-linked Antibody (Cell Signaling Technology, 1:3500), Goat-anti-mouse IgG Light Chain HRP (1:5000), Goat Anti-Rat IgG H&L (HRP) (Abcam, 1:50000)) and imaged with the ChemiDoc Imaging System (Bio-Rad). Relative protein levels were quantified using Image Lab (Bio-Rad).

### Co-Immunoprecipitation assays

HEK-293T cells from one confluent 35-mm dish were lysed in 100 μl of Frackelton lysis buffer (10 mM Tris (pH 7.4), 50 mM NaCl, 30 mM Na_4_P_2_O_7_, 50 mM NaF, 2 mM EDTA, 1% Triton X-100, 1 mM DTT, 1 mM PMSF (Sigma-Aldrich), and 1X cOmplete Protease Inhibitor Cocktail (Roche)). Cells were incubated on a rotating wheel at 4 °C for 30 min and subsequently centrifuged at 20,000 x *g* at 4 °C for 30 min. The supernatant was transferred to a new tube and 10 μl (10% of the lysate used for the IPs) was collected as input. 500 μg of lysates were incubated overnight at 4 °C on a rotating wheel with anti-HA antibody (Cell Signaling Technology, 1:100). For indicated samples incubated with RNase A, lysates were incubated with RNase A (100 µg/mL) for 2 h at 4 °C prior to antibody addition. The next day, magnetic beads (Protein A/G Magnetic Beads, Thermo Fisher Scientific) used for anti-HA antibody IPs were blocked by rotation in 3% BSA in Frackelton Buffer for 1 h at 4 °C. 25 μl of beads were added to 500 μg of lysates and rotated for 2 h at 4 °C. Then, the beads were washed five times with 1 ml of Frackelton lysis buffer. Proteins were eluted by boiling in 2X Disruption buffer for 10 min at 95 °C.

### Immunoprecipitations for ubiquitination

HEK-293T cells from confluent 35-mm dishes were lysed in 100 µl of RIPA buffer with 1% SDS (50 mM Tris-HCl (pH 7.4), 150 mM NaCl, 1% SDS, 0.5% sodium deoxycholate, 1% Triton X-100), supplemented with 40 mM N-Ethylmaleimide, 40 mM iodoacetamide, 25 U/ml Benzonase, 1 mM PMSF (Sigma-Aldrich), and 1X cOmplete Protease Inhibitor Cocktail (Roche). Cells were incubated on a rotating wheel at 4 °C for 30 min and centrifuged at 20,000 x *g* at 4 °C for 15 min. Supernatants were transferred to new tubes, and 30 µg of the lysates were collected as input. 500 μg of lysates were incubated overnight at 4 °C on a rotating wheel with an anti-HA antibody (Cell Signaling Technology, 1:100). The next day, magnetic beads (Protein A/G Magnetic Beads, Thermo Fisher Scientific) were blocked by rotation in 3% BSA in RIPA Buffer for 1 h at 4 °C. 25 μl of beads were added to 500 μg of lysates and rotated for 2 h at 4 °C. Subsequently, beads were washed five times with 1 ml of RIPA buffer, supplemented with 300 mM NaCl. Proteins were eluted by boiling in 2X Disruption buffer for 10 min at 95 °C.

### RNA isolation, cDNA synthesis, and qPCR

For mRNA half-life determination, cells were treated with actinomycin D (ActD) at a final concentration of 5 µg/ml for the indicated times, at which point total RNA was isolated. The decay of *APOBEC3H* mRNA was assessed by RT-qPCR. RNA from 1 × 10^6^ cells was isolated using Trizol reagent (Thermo Fisher Scientific) and treated with Turbo DNase (Thermo Fisher Scientific). cDNA was prepared using random hexamer primers and RevertAid Reverse Transcriptase (Thermo Fisher Scientific). Real-time PCR experiments were run on a Mastercycler (Biorad), using the Luna Universal qPCR Master Mix (NEB). Primers for qPCR are listed in the Supplementary Methods.

### TurboID

A3H-I and A3H-II, as well as an EGFP control were cloned into lentiviral plasmid pCW-MYC-TurboID-MCS-PGK-mCherry-P2A-rtTA^48^, for the expression of fusion proteins N-terminally tagged with TurboID. For the TurboID experiment, polyclonal cell lines stably expressing DOX-inducible TurboID-A3H-I, TurboID-A3H-II, and TurboID-EGFP were stimulated with DOX for 48 h to induce the expression of the TurboID fusion constructs. Subsequently, cells were stimulated for 5 h with 10 µM EPOX to inhibit proteasomal degradation, and finally biotinylation was induced for 15 min by addition of 500 µM Biotin (Sigma) to the cell culture medium. Subsequently, cells were washed 4 times with ice-cold PBS, prior to lysis in RIPA lysis buffer (50 mM Tris HCl (pH 7.4), 150 mM NaCl, 1% NP-40, 0.5% Sodium Deoxycholate, 1 mM EDTA, 0.1% SDS, 1 mM PMSF (Sigma-Aldrich) and 1X cOmplete Protease Inhibitor Cocktail (Roche). Lysates were rotated for 30 min. at 4 °C, centrifuged at 18,500 x *g* for 10 min at 4 °C, and protein concentrations were determined by BCA assay. 1200 µg of protein was incubated overnight, rotating at 4 °C with 200 µL of Streptavidin beads (Thermo Scientific), which were acetylated with Sulfo-NHS-Acetate beforehand, as described^135^. Beads were washed twice with 1 ml of RIPA buffer, once with 1 ml of 2 M Urea in 10 mM Tris (pH 8), twice with 1 ml RIPA buffer and five times with 50 mM HEPES (pH 7.8). Three technical replicates were subjected to nLC-MS/MS analysis. Statistical analysis was conducted using moderated t-statistics via the limma-trend method in R and applying the Benjamini–Hochberg multiple testing correction. The TurboID results generated in this study are provided as Supplementary Data 2.

### Sample preparation for mass spectrometry analysis

Beads were resuspended in 50 µl 1 M Urea and 50 mM ammonium bicarbonate. Disulfide bonds were reduced with 2 µl of 250 mM dithiothreitol (DTT) for 30 min at room temperature before adding 2 µl of 500 mM iodoacetamide and incubating for 30 min at room temperature in the dark. Remaining iodoacetamide was quenched with 1 µl of 250 mM DTT for 10 min. Proteins were digested with 150 ng LysC (mass spectrometry grade, FUJIFILM Wako chemicals) at 25 °C overnight. The supernatant was transferred to a new tube and digested with 150 ng trypsin (Trypsin Gold, Promega) in 1.5 µl 50 mM ammonium bicarbonate at 37 °C for 5 h. The digest was stopped by the addition of trifluoroacetic acid (TFA) to a final concentration of 0.5%, and the peptides were desalted using C18 Stagetips^136^.

### Liquid chromatography-mass spectrometry data acquisition

Peptides were separated on an Ultimate 3000 RSLC nano-flow chromatography system (Thermo-Fisher), using a pre-column for sample loading (Acclaim PepMap C18, 2 cm × 0.1 mm, 5 μm, Thermo-Fisher), and a C18 analytical column (Acclaim PepMap C18, 50 cm × 0.75 mm, 2 μm, Thermo-Fisher), applying a segmented linear gradient from 2 to 35% and finally 80% solvent B (80% acetonitrile, 0.1 % formic acid; solvent A 0.1 % formic acid) at a flow rate of 230 nl/min. over 120 min. Eluting peptides were analyzed on an Exploris 480 Orbitrap mass spectrometer (Thermo Fisher) coupled to the column with a FAIMS pro ion-source (Thermo-Fisher) using coated emitter tips (PepSep, MSWil) with the following settings: The mass spectrometer was operated in DDA mode with two FAIMS compensation voltages (CV) set to −45 or −60 and 1.5 s. cycle time per CV. The survey scans were obtained in a mass range of 350–1500 m/z, at a resolution of 60 k at 200 m/z, and a normalized AGC target at 100%. The most intense ions were selected with an isolation width of 1 m/z, fragmented in the HCD cell at 28% collision energy, and the spectra recorded for max. 50 ms. at a normalized AGC target of 100% and a resolution of 15 k. Peptides with a charge of +2 to +6 were included for fragmentation, the peptide match feature was set to preferred, the exclude isotope feature was enabled, and selected precursors were dynamically excluded from repeated sampling for 45 s.

### Data analysis

MS raw data split for each CV using FreeStyle 1.7 (Thermo Fisher) were analyzed using the MaxQuant software package (version 1.6.17.0)^137^ with the Uniprot human reference proteome (version 2020_01)^138^, as well as a database of the most common contaminants. The search was performed with full trypsin specificity and a maximum of two missed cleavages at a protein and peptide spectrum match false discovery rate of 1%. Carbamidomethylation of cysteine residues was set as fixed, oxidation of methionine, phosphorylation (STY), and N-terminal acetylation as variable modifications. For label-free quantification, the “match between runs” only within the sample batch and the LFQ function were activated - all other parameters were left at default. MaxQuant output tables were further processed in R 4.2.1^139^ using Cassiopeia_LFQ^140^. Reverse database identifications, contaminant proteins, protein groups identified only by a modified peptide, protein groups with less than two quantitative values in one experimental group, and protein groups with less than 2 razor peptides were removed for further analysis. Missing values were replaced by randomly drawing data points from a normal distribution model on the whole dataset (data mean shifted by −1.8 standard deviations, a width of the distribution of 0.3 standard deviations). Differential expression analysis was performed with Enrichr^141–143^. In detail, differentially enriched proteins in A3H-I/GFP (light blue, LFC > 1, p-value < 0.01) and A3H-II/GFP (dark blue, LFC > 1, p-value < 0.01) were compared using RStudio (v4.1.3)^144^. GO:CC and GO: BP terms were derived for proteins being specifically enriched in the A3H-I or A3H-II interactome, or for shared interactors.

### Purification of recombinant APOBEC3 proteins

BL21 (DE3) RIPL *E. coli* cells harboring 10xHis-MBP-A3 expression vectors were grown at 37 °C. IPTG at 0.3 mM was added when OD600 reached about 0.6–0.8 for overnight induction at 18 °C. To purify 10xHis-MBP-fused A3, *E. coli* cells expressing MBP-A3H-I/II/II-E56A-W115A-R175/176E, A3A, A3B CD1 wt or A3B CD2 wt were harvested and homogenized in buffer A (25 mM HEPES pH 7.5, 500 mM NaCl, 20 mM imidazole, 5% glycerol, 5 mM β-Mercaptoethanol, 1X EDTA-free cOmplete Protease Inhibitor Cocktail (Roche), 0.5 mM PMSF (Sigma-Aldrich), 100 ug/ml RNase A (Carl Roth)). A3B constructs were homogenized with only 10 µg/mL RNase A (Carl Roth). The clear soluble fraction obtained after centrifugation (45 min at 18.000 x *g*) was loaded onto a HisTrap HP 5 mL, washed with wash buffer (25 mM HEPES pH 7.5, 500 mM NaCl, 20 mM imidazole, 5% glycerol, 5 mM β-Mercaptoethanol, 10 μg/ml RNase A (Carl Roth)) and eluted with buffer B (25 mM HEPES pH 7.5, 500 mM NaCl, 1 M imidazole, 5% glycerol, 5 mM β-Mercaptoethanol). Fractions containing A3 were identified by SDS page, concentrated if possible, and separated by gel filtration chromatography in a Superdex 200 16/600 column (Cytiva) in SEC buffer (25 mM HEPES pH 7.5, 250 mM NaCl, 5% glycerol, 2 mM DTT. The dimer and monomer fractions were collected, concentrated if possible and flash frozen in liquid nitrogen.

Codon-optimized human HUWE1 was synthesized in fragments and assembled into a GoldenBac pGBdest vector using a BsaI-GoldenGate reaction^145^. The construct had a his_8_ tag followed by a rigid enhancer linker (AEAAAKEAAAKEAAAKEAAAKALEAEAAAKEAAAKEAAAKEAAAKA) and then a TEV cleavage site. Human UBR4 was cloned by the same procedure, except the tag used was a C-terminal Strep tag separated from the protein by a 3 °C cleavage site. Plasmids were transformed into DH10 MultiBac cells for bacmid generation. *Spodoptera frugiperda* (Sf9) cells in ESF921 serum-free growth medium (Expression Systems) were transfected with the bacmids for virus amplification, which was monitored using yellow fluorescent protein signal. HUWE1 and UBR4 were then expressed in *Trichoplusia ni* High-Five insect cells (Thermo Fisher) with a density of 1.5 × 10^6^ using a 1:70 inoculation from the V1 stock for 92 h at 21 °C in Insect Xpress Protein-free Insect Cell Medium (Lonza) supplemented with GlutaMAX (GIBCO) and Pen/Strep Amphotericin B (Lonza). Cells were harvested by centrifugation at 700 × *g*, washed in PBS, and then flash frozen and stored at −70 °C. For HUWE1, the thawed pellet was resuspended in 50 mM HEPES pH 7.5, 300 mM NaCl, 0.5 mM TCEP, 20 mM imidazole with Complete EDTA-free Protease inhibitor (Roche) and 20 µl Benzonase (IMP Molecular Biology Service) and lysed using dounce homogenization. The lysate was cleared by centrifugation at 40,000 × *g*. The soluble fraction was then loaded on a 5 ml HisTrap HP column (Cytiva) pre-equilibrated in the lysis buffer using an Äkta Pure 25 system (Cytiva). The column was washed with 10 column volumes of the same buffer, followed by 7 column volumes of the same buffer but with 35 mM imidazole, and then eluted with 300 mM imidazole. The protein was cleaved with TEV protease and simultaneously dialyzed to remove imidazole overnight. The cleaved protein was then reapplied to the HisTrap HP column in 20 mM imidazole and the flow through was collected and concentrated to 1.5 ml. Finally, the protein was applied to a Superose 6 16/60 column (Cytiva) equilibrated in 50 mM HEPES pH 7.5, 150 mM NaCl, 0.5 mM TCEP. Protein-containing fractions were pooled and concentrated. The protein was pure as assessed by SDS-PAGE, and the concentration was estimated by A280 absorption using an extinction coefficient of 251,770 M^−1 ^cm^−1^. For UBR4, the same lysis procedure was used except with phosphate buffered saline (PBS), 0.5 mM TCEP at pH 7.4 as the buffer. The soluble lysate was then loaded on a 5 ml StrepTrap HP column (Cytiva) equilibrated in PBS, washed with the same buffer and then eluted with 2.5 mM desthiobiotin. Eluted UBR4 was further applied to a Resource Q column (Cytiva) equilibrated in PBS for further purification by anion exchange chromatography using a 250–500 mM NaCl gradient. UBR4-containing fractions were pooled and concentrated and the final concentration measured by absorption at 280 nm using a calculated extinction coefficient of 472,140 M^−1 ^cm^−1^ The codon-optimized UBE2A gene was synthesized by Twist Bioscience. The NEB HiFi assembly kit (New England Biolabs) was used to clone the gene into a pET29b expression vector, which contained an N-terminal His6-tag and TEV cleavage site. Expression was performed using *E. coli* BL21 (DE3) cells with IPTG induction at 20 °C overnight. Cells were resuspended in buffer containing 50 mM Tris pH 7.5, 150 mM NaCl, 10 mM imidazole, Complete EDTA-free protease inhibitor cocktail. Cells were lysed by sonication and clarified by centrifugation at 18,500 *g* for 20 min at 4 °C. Clarified lysates were then incubated with Ni-NTA resin (Qiagen) for 1 h at 4 °C with mild agitation. Ni-NTA resin was washed before elution with 150 mM imidazole. UBE2A was further purified by SEC using a Superdex 75 16/600 column (Cytiva) equilibrated in 50 mM Tris, 150 mM NaCl, and 0.5 mM TCEP, pH 7.5. Recombinant human UBR5, Ubiquitin, DyLight488-labeled Ubiquitin, UBE2D3, and UBA1 were purified as described previously^104,120,146^.

### UREA denaturing gel

To determine the amount of bound RNA, equal protein amounts of 10xHis-MBP-A3H-I/II/II-E56A-W115A-R175/176E were denatured by the addition of 2x denaturing buffer (0.6 g/ml urea, 0.1% SDS, 1 mM EDTA pH 8.0, 0.5 mg/ml Xylene Cyanol, 0.5 mg/ml Bromophenol blue) and incubation for 5 min at 95 °C. Subsequently, the RNA was separated from the proteins on a 6%Urea-TBE gel and visualized by SYBR Gold staining.

### In vitro ubiquitination assays

In vitro ubiquitination assays contained 10 μM ubiquitin, 1 μM DyLight488-labeled ubiquitin for in-gel visualization, 0.2 μM E1 (UBA1), 0.4 μM E2 (UBE2A for UBR4, UBE2D3 for UBR5 and HUWE1), 0.4 μM E3 (UBR4, UBR5, HUWE1) and 4 μM substrate protein (10xHis-MBP-A3H), unless indicated otherwise. The assays were performed in 25 mM HEPES pH 7.5, 200 mM NaCl, 5 mM MgCl2, 0.5 mM TCEP (assay buffer) at 30 °C (UBR5) or 37 °C (UBR4 and HUWE1) for 1 h in 11 μl volumes. Reactions were initiated by the addition of 5 mM ATP. Given their low purification yield, reactions with 10xHis-MBP-A3A/A3B-CD1/A3B-CD2 were performed with only half the concentration. After one hour, reactions were diluted in 500 µl immune-precipitation buffer (RIPA buffer supplemented with 40 mM N-Ethylmaleimide (Sigma-Aldrich), 40 mM iodoacetamide (Sigma-Aldrich), 1 mM PMSF (Sigma-Aldrich), and 1X cOmplete Protease Inhibitor Cocktail (Roche) and incubated with 15 µl of anti-MBP couped beads (pre-blocked for 1 h in RIPA with 3 % BSA; NEB) for 2 h at 4 °C. Beads were washed 5 times with RIPA-300 mM NaCl, followed by elution of A3H in 2x Laemmli sample buffer with 2 mM DTT. SDS-PAGE was performed using 4–20% Mini-PROTEAN TGX Stain-Free (BioRad), and ubiquitinated A3H was detected by visualization of DyLight488-ubiquitin using ChemiDoc MP system (Bio-Rad).

### Sucrose gradient binding assays

Density gradient centrifugations were performed by preparing the low-density solution of 10% (w/v) sucrose and high-density solution of 30% (w/v) sucrose in buffer G (100 mM NaCl, 50 mM HEPES pH 7.5, and 0.5 mM TCEP). 100 pmol of an APOBEC construct with 150 pmol of UBR5 or buffer were mixed and dialyzed into bufferG for 45 min at room-temperature and subsequently applied onto the gradient prepared in an open-top 4.2 ml ultracentrifuge tube and run for 16 h at 105,350 *g* in an SW60Ti rotor. For samples treated with RNase A, 100 pmol of an APOBEC construct or 150 pmol of UBR5 were incubated with 1 mg/ml of RNase A at 30 °C or buffer for 30 min and subsequently mixed and dialyzed into buffer. Gradients were manually fractionated into 200 µL fractions and analysed using 4–15% Criterion Stain-free Midi gels (BioRad) with stain-free imaging or WB (TransBlot Turbo BioRad) against the APOBEC His-tag.

### Purification of recombinant HUWE1 for analytical size exclusion analysis

Codon-optimized full-length human HUWE1 (Twist Bioscience) was cloned into the pMEPI vector with an N-terminal 6x-His tag, a 3 °C protease cleavage site, and a C-terminal double Strep-tag. Stable HEK293T cell lines were generated via co-transfection with PiggyBac transposase under a Tet-inducible promoter with puromycin selection. Cells were cultured in FreeStyle 293 medium (GIBCO), and protein expression was induced with 1 µg/mL doxycycline for 48 h. After 24 h, 125 mL Ex-Cell 293 medium (Sigma Aldrich) was added per 0.5 L culture. Cells were harvested by centrifugation (2000 *g*, 20 min), washed in PBS, and flash frozen. Cell pellets were resuspended in Buffer A (30 mM HEPES pH 7.4, 0.3 M NaCl, 0.5 mM TCEP) with protease inhibitors and Benzonase, lysed using a high-pressure cell disruptor (1.0 kBar), and clarified by centrifugation (40,000 *g*, 1 h). The supernatant was loaded onto a StrepTrap HP column (Cytiva), washed with 10 column volumes of Buffer A, and eluted with Buffer A + 5 mM desthiobiotin. Eluate was diluted to 50 mM NaCl in RQA buffer (30 mM HEPES pH 7.4, 1 mM TCEP) and loaded onto a Resource Q column (GE Healthcare). Protein was eluted using a linear gradient (0.05–0.74 M NaCl). Peak fractions were pooled and further purified by size-exclusion chromatography using a Superose 6 10/300 column (Cytiva) in GF buffer (30 mM HEPES pH 7.5, 150 mM NaCl, 2 mM TCEP). Final protein was concentrated and flash-frozen in liquid nitrogen.

### mutREAD sequencing and detection of mutational signatures

Monoclonal RKO-DOX-Cas9-P2A-GFP cell lines that also have a homozygous knock-out of *UNG2*^-/-^ and either nothing or MYC-mCherry-P2A-3xHA-A3H-I over-expressed were generated. As a control the cell line without A3H-I over-expression was transduced with sg*A3B* to generate A3B knock-out cells. These cells were transduced with DualCRISPR-hU6-sgRNA-mU6-sgRNA-iRFP670 targeting indicated genes and bulk sorted for iRFP^+^ cells. Cas9 was induced with DOX and sequential samples collected up to 10 days after induction. Cells were harvested on Day 6 and Day 10 after Cas9 induction. E3 ligase knock-outs were verified by WB and gDNA extracted. DNA was isolated using the DNeasy Blood & Tissue Kit (Qiagen) after which samples were subjected to mutREAD library preparation as described^68^. In brief, 500 ng gDNA was digested with 50 U PstI-HF (NEB) and 50 U ApoI-HF (NEB) together with 0.187 µM annealed mutREAD adapters (see Supplementary Methods), 400 U T4 DNA ligase (NEB), 1 mM ATP in 1X CutSmart buffer. The 50 µl reaction was incubated for 3 h at 30 °C in a thermal cycler and subsequently stopped by addition of 10 µL 50 mM EDTA. DNA fragments were size selected for 400-600 bp fragments performing a 0.6X/0.15X 2-sided size selection using DNA clean beads (VAHTS). Size-selected DNA fragments were amplified using NEB unique multiplexed i5 and i7 primers (E6440) in a total reaction volume of 100 µl together with 2 U Phusion High-Fidelity DNA Polymerase (NEB) and in the presence of 0.2 mM dNTPs and 1X Phusion High-Fidelity buffer. PCR was performed in the following conditions: 98 °C/2 min. denaturation, 12 cycles of amplification at 98 °C/10 s, 65 °C/30 s, 72 °C/30 s and final extension at 72 °C for 5 min. Subsequently, libraries were selected by again performing a 0.6X/0.15X 2-sided size selection after which samples were eluted in 20 μl TE buffer (Tris-EDTA buffer 10 mM Tris-HCl and 0.1 mM EDTA, pH 8) and stored at −20 °C. Quality and fragment sizes were evaluated on a fragment analyzer (Advanced Analytical Technologies) and quantified using Picogreen assay (Invitrogen). Equimolar ratios of the obtained libraries were pooled and 150 bp paired-end sequenced on an Illumina NovaSeq S4 platform.

### Alignment and somatic variant detection

Raw reads were processed according to GATK4 best practices^147^. Briefly, reads were mapped to the GRCh38 human reference genome and single nucleotide substitutions and InDels were called with Mutect2 in tumor-only mode^148^. Resulting variants were filtered by only retaining variants with Mutect2 quality status ‘PASS’ and variant allele frequency (VAF) between 0.3 and 0.7. In order to exclude cell-line specific variants found in the parental clone, we obtained 30x whole genome sequencing (BGITech Global, BGI Genomics Co., Ltd.) from the parental clone (RKO-DOX-Cas9-P2A-GFP) and retained only variants which were not also present in the parental clone.

### Bootstrapped background correction

We applied a bootstrapping strategy to construct an average background mutational profile from the samples obtained 3 days after transduction with the control sgRNA vector targeting *AAVS1* (n = 3). First, to reduce the effect of outliers, the mutational count of all samples was down-scaled proportional to the sample with the lowest mutation count. For each sample, 10,000 bootstrap samples were generated using weighted random multinomial sampling, with the underlying distribution of mutation counts across the 96 features serving as weights. This process yielded 30,000 bootstrapped samples in total, from which the mean mutation count across the 96 mutation channels was computed, resulting in the final average background profile. The average background mutational profile was subsequently subtracted from the mutational matrix of the samples using element-wise subtraction with thresholding at 0.

### Mutational signature analysis

We conducted signature refitting and plotting using the MutationalPatterns package^149^ in R. Refitting was accomplished by incorporating a catalog of signatures frequently active in colon carcinoma, alongside APOBEC-related signatures (SBS2 and SBS13), using the best subset refit approach with the parameter max_delta set to 0.004 (recommended) to obtain a strict estimate of signature activity. The significance of the refitting results was assessed using the signature activity test from the mSigAct package^150^.

### Pentanucleotide context analysis

The nucleotide preference in pentanucleotide contexts was assessed by first collecting the nucleotides up to the +2 and −2 position around all C > T and C > G mutations, filtering all resulting k-mers for NTCNN sequences, and plotting the frequency of the nucleotides per position using plotnineSeqSuite^151^.

### Computational analysis of cancer genome sequencing database

SBS mutational signatures analyzed in the PCAWG study were obtained from the ICGC Data portal^152^. Corresponding VCF files for each sample were either retrieved from the ICGC Data portal or through granted access of the TCGA Research Network^153^. To identify the genotype of a specific gene of interested (E3 ligase) in each sample, the corresponding VCF files (snv and indel) were tested for variants within the location of the coding gene. Samples exhibiting at least one variant in either snv or indel with a Variant Classification not being “Intron”, were categorized as “mut”. Conversely, the remaining samples were categorized as “wt”. SBS mutational signatures were normalized to the total number of mutations in each sample and the fraction of SBS signatures of interest compared between “wt”’ and “mut” groups. Four E3 ligase genes (*HECTD4*, *NEDD4L*, *HECT1*, *PCHF3*), with a similar number of “mut” cancers were randomly selected and used for probing specificity.

### Quantification and statistical analysis

Sample number and number of biological replicates are indicated in the figure legends of all figures. Statistical analyses were performed with the R (v4.0.2) programming environment using RStudio (v4.1.3) or GraphPad Prism 10.0.2.

### Statistics and reproducibility

All Western blots presented within individual figure panels are from the same experiment and gels/blots were processed in parallel. The experiments represented in Figs. 1C, 3G, H, 4G, H, 5A–J were repeated twice independently with similar results. The experiments represented in Fig. 4C, D, were repeated independently three times with similar results. The experiments represented in Figs. 4I and 5K were performed once. The experiments represented in Supplementary Fig. 1E, G, 4C, 5B, E–G, 6B, 8A, C, E, G were repeated twice independently with similar results. The experiments represented in Supplementary Figs. 4D, 7C, 9B were repeated independently three times with similar results. The experiments represented in Supplementary Figs. 1B, 7D, 9A were performed once.

### Reporting summary

Further information on research design is available in the Nature Portfolio Reporting Summary linked to this article.

## Supplementary information

Supplementary Information

Description of Additional Supplementary Files

Supplementary Data 1

Supplementary Data 2

Reporting Summary

Transparent Peer Review file

## Source data

Source data

## Data Availability

Cancer genetic analysis data are available from the ICGC Data portal and through granted access of the TCGA Research Network (https://www.cancer.gov/ccg/research/genome-sequencing/tcga). The mass-spectrometry data generated in this study are available in Supplementary Data 2 and have been deposited to the ProteomeXchange consortium via the PRIDE partner repository with the accession code PXD051267 Public cancer whole-genome sequencing data from ICGC are available through the ICGC Data Portal (public) (https://dcc.icgc.org/releases/PCAWG). The NCBI dbGaP data are under restricted access as per NIH policy, access can be obtained through (https://dbgap.ncbi.nlm.nih.gov/home/).The genetic screen data generated in this study are provided as Supplementary Data 1 and in the Source Data file. Source data are provided with this paper.

## Code availability

Data and code pertaining to cancer genome analysis and sequence context analysis can be found on GitHub (https://github.com/menchelab/apobex)^154^.
