# Supplementary material for: Guardian ubiquitin E3 ligases target cancer-associated APOBEC3 deaminases for degradation to promote human genome integrity
Source: Nat Commun. 2026 Jan 19;17:1723. doi: 10.1038/s41467-026-68420-5 (PMC12913773; doi:10.1038/s41467-026-68420-5)
Supplement: Supplementary file 1 — Supplementary Information [file 41467_2026_68420_MOESM1_ESM.pdf]

Supplementary Methods

Reagents and Resources

REAGENT or RESOURCE	DILUTION	SOURCE	IDENTIFIER
**Antibodies**			
ARP10 Antibody	1:1000 for WB<br>1:100 for IF	Novus<br>1:1000	Cat# NBP1-91682; RRID:AB_11036260
Anti-APOBEC3B Antibody (EPR18138)	1:1000	Abcam<br>1:1000	Cat# ab184990; RRID:AB_2891094
Anti-APOBEC3G (D9C6Z) Rabbit mAb	1:1000	Cell Signaling Technology<br>1:1000	Cat# 43584<br>RRID:AB_2799245
Anti-MYC antibody (4A6)	1:5000	Millipore<br>1:5000	Cat# 05-724; RRID:AB_11211891
HA-Tag (C29F4) Rabbit mAb	1:1000 for WB<br>1:100 for Co-IP	Cell Signaling Technology	Cat# 3724; RRID:AB_1549585
HA-Tag (6E2) Mouse mAb	1:1000 for WB<br>1:100 for Co-IP	Cell Signaling Technology	Cat# 2367; RRID:AB_10691311
OLLAS Epitope Tag Antibody (L2)	1:4000	Novus	Cat# NBP1-06713; RRID:AB_1625979
LC3B Antibody	1:1000	Cell Signaling Technology	Cat# 2775; RRID:AB_915950
Ubiquitin Antikörper (P4D1)	1:1000	Santa Cruz Biotechnology	Cat# sc-8017; RRID:AB_628423
Anti-UBR4/p600 antibody	1:1000	Abcam	Cat# ab86738; RRID:AB_1952666
Rabbit anti-EDD1 Antibody	1:1000	Bethyl	Cat# A300-573A; RRID:AB_2210189
Rabbit anti-Lasu1/Ureb1 Antibody (HUWE1)	1:1000	Bethyl	Cat# A300-486A; RRID:AB_2264590
XBP-1s (E9V3E) Rabbit mAb	1:1000	Cell Signaling Technology	Cat# 40435; RRID: AB_2891025
Monoclonal Anti-α-Tubulin antibody produced in mouse	1:1000	Sigma-Aldrich	Cat# T9026; RRID:AB_477593
Lamin A/C Antibody (E-1)	1:1000	Santa Cruz Biotechnology	Cat# sc-376248; RRID:AB_10991536
Monoclonal Anti-Vinculin antibody	1:1000	Sigma-Aldrich	Cat# V9131; RRID:AB_477629
Penta·His Antibody, BSA-free		Qiagen	Cat# 34660<br>RRID:AB_2619735
Anti-beta Actin antibody (HRP) (AC-15)	1:20000	Abcam	Cat# ab49900; RRID:AB_867494
Anti-rabbit IgG, HRP-linked Antibody	1:3500	Cell Signaling Technology	Cat# 7074; RRID:AB_2099233
Anti-mouse IgG, HRP-linked Antibody	1:3500	Cell Signaling Technology	Cat# 7076; RRID:AB_330924
Goat-anti-mouse IgG Light Chain HRP	1:5000	Jackson ImmunoResearch Labs	Cat# 115-035-174; RRID:AB_2338512
Goat Anti-Rat IgG H&L (HRP)	1:50000	Abcam	Cat# ab97057<br>RRID:AB_10680316
APC anti-rat CD90/mouse CD90.1 (Thy-1.1) Antibody	1:260	BioLegend	Cat# 202526; RRID:AB_1595470
Donkey F(ab')2 Anti-Rabbit IgG - H&L (Alexa Fluor 488), pre-adsorbed	1:800 for IF	Abcam	Cat# ab181346; RRID:AB_2813899

**Bacterial and virus strains**		
BL21-CodonPlus (DE3)-RIPL Competent Cells	Agilent	Cat# 230280
Spodoptera frugiperda (Sf9)	Expression Systems	N/A
High Five™ Cells in Express Five™ Medium	Thermo Fisher Scientific	Cat# B85502
**Biological samples**		
Whole-genome sequencing from PCAWG - PanCancer Analysis of Whole Genomes	ICGC - International Cancer Genome Consortium<br>TCGA - The Cancer Genome Atlas	https://icgc.org/<br>https://www.cancer.gov/ccg/research/genome-sequencing/tcga
**Chemicals, peptides, and recombinant proteins**		
Cycloheximide solution	Sigma-Aldrich	Cat# C4859
MG-132	Sigma-Aldrich	Cat# M7449
Epoxomicin	Gentaur Molecular Products	Cat# 607-A2606
Chloroquine diphosphate salt	Sigma-Aldrich	Cat# C6628
Bafilomycin A1	Santa Cruz Biotechnology	Cat# sc-201550
Ammonium Chloride	Applichem	Cat# A3260
Leupeptin	Enzo Life Science	Cat# ALX260009
Doxycycline hyclate	Sigma-Aldrich	Cat# D9891
G418 disulfate salt	Sigma-Aldrich	Cat# A1720
Actinomycin D	Sigma-Aldrich	Cat # A1410
Benzonase Nuclease	Merck	Cat# 70746
Ribonuclease A	Carl-Roth	Cat# 7156
SYBR™ Gold Nucleic Acid Gel Stain (10,000X Concentrate in DMSO)	Thermo Fisher Scientific	Cat# S11494
Biotin	Sigma-Aldrich	Cat# B4501
Polybrene	Sigma-Aldrich	Cat# TR1003G
Polyethylenimine	Polysciences	Cat# 23966
cOmplete™ Protease Inhibitor Cocktail	Roche	Cat# 11697498001
cOmplete™, EDTA-free Protease Inhibitor Cocktail	Roche	Cat# 11873580001
N-Ethylmaleinimid	Sigma-Aldrich	Cat# E3876
Iodoacetamide	Sigma-Aldrich	Cat# I1149
Hoechst 33258	Thermo Fisher Scientific	Cat# H3569
TRIzol™ Reagent	Invitrogen	Cat# 15596018
Invitrogen™ TURBO™ DNase (2 U/µL)	Thermo Fisher Scientific	Cat# 10792877
RevertAid Reverse Transcriptase (200 U/µL)	Thermo Fisher Scientific	Cat# EP0441
Trypsin Gold	Promega	Cat# V5280
PstI-HF®	New England Biolabs	Cat# R3140
ApoI-HF®	New England Biolabs	Cat# R3566
Phusion® High-Fidelity DNA Polymerase	New England Biolabs	Cat# M0530
ProLong™ Gold Antifade Mountant	Invitrogen	Cat# P36934
T4 DNA Ligase	New England Biolabs	Cat# M0202
PMSF (Phenylmethylsulfonyl fluoride)	Sigma-Aldrich	Cat# P7626
2-Mercaptoethanol	Carl-Roth	Cat# 4227.3
Bovine Serum Albumin; BSA >96%	Sigma-Aldrich	Cat# A2153
Human BD Fc Block	BD Biosciences	Cat# 564220
Recombinant 10xHis-MBP-A3H-I	This study	N/A
Recombinant 10xHis-MBP-A3H-II	This study	N/A
Recombinant 10xHis-MBP-A3H-II RBM	This study	N/A

UBA1	Grabarczyk D.B., Petrova O.A., *et al.*, 2021<br>Ehrmann J.F., Grabarczyk D.B., et al., 2023	N/A
UBE2A	This study	N/A
UBE2D3	Grabarczyk D.B., Petrova O.A., *et al.*, 2021<br>Ehrmann J.F., Grabarczyk D.B., et al., 2023	N/A
UBR4	This study	N/A
UBR5	Hodáková Z., Grishkovskaya I., *et al.*, 2023	N/A
HUWE1	This study	N/A
ATP	Sigma	Cat# A7699
Ubiquitin	Grabarczyk D.B., Petrova O.A., *et al.*, 2021<br>Ehrmann J.F., Grabarczyk D.B., et al., 2023	N/A
Dy488-Ubiquitin	Grabarczyk D.B., Petrova O.A., *et al.*, 2021<br>Ehrmann J.F., Grabarczyk D.B., et al., 2023	N/A
**Critical commercial assays**		
Pierce™ BCA Protein Assay Kits	Thermo Fisher Scientific	Cat# 23225
DNeasy Blood & Tissue Kits	Qiagen	Cat# 69506
Quant-iT™ PicoGreen™ dsDNA Assay Kits and dsDNA Reagents	Invitrogen	Cat# P7589
**Deposited data**		
Genetic screen Dual-A3H-reporter	This study	Suppl. Data 1
TurboID Mass spectrometry	This study	Suppl. Data 2<br>PRIDE: PXD051267
**Experimental models: Cell lines**		
HEK293T	ATCC	CRL-3216
Lenti-X™ 293T Cell Line	Takara	Cat# 632180
HeLa	ATCC	CCL-2
RKO	ATCC	CRL-2577
RKO-DOX-Cas9-P2A-BFP	Michlits G., *et al.*, 2020	N/A
RKO-DOX-Cas9-P2A-GFP	de Almeida M., Hinterndorfer M. *et al.*, 2021	N/A
RKO-MYC-mCherry-P2A-OLLAS-A3H-I	This study	N/A
RKO-MYC-mCherry-P2A-OLLAS-A3H-II	This study	N/A
RKO-DOX-Cas9-mCherry-A3H-II-P2A-EGFP-A3H-I	This study	N/A
RKO-DOX-Cas9-MYC-mCherry-P2A-3xHA-A3H-I	This study	N/A
THP-1	ATCC	TIB-202
THP-1-DOX-Cas9-P2A-GFP	This study	N/A
RKO-DOX-MYC-TurboID-A3H-I-mCherry-P2A-rtTA	This study	N/A
RKO-DOX-MYC-TurboID-A3H-II-mCherry-P2A-rtTA	This study	N/A
RKO-DOX-MYC-TurboID-GFP-mCherry-P2A-rtTA	This study	N/A

RKO-DOX-Cas9-MYC-mCherry-P2A-OLLAS-EGFP-A3H-I	This study	N/A
RKO-DOX-Cas9-MYC-mCherry-P2A-OLLAS-EGFP-A3H-II	This study	N/A
RKO-DOX-Cas9-MYC-mCherry-P2A-OLLAS-EGFP-A3H-II-W155A	This study	N/A
RKO-DOX-Cas9-MYC-mCherry-P2A-OLLAS-EGFP-A3H-II-R175/176E	This study	N/A
RKO-DOX-Cas9-MYC-mCherry-P2A-OLLAS-EGFP-A3H-I-G105R	This study	N/A
RKO-DOX-Cas9-MYC-mCherry-P2A-OLLAS-EGFP-A3H-II-R105G	This study	N/A
RKO-DOX-Cas9-MYC-mCherry-P2A-OLLAS-EGFP-A3H-II-E56A-W155A-R175/176E	This study	N/A
RKO-DOX-Cas9-MYC-mCherry-P2A-3xHA-A3H-I UNG2-/-	This study	N/A
**Oligonucleotides**		
qPCR primer: mCherry-P2A (A3H) fwd: CTCACTACTCAAACAAGCAG	This study	N/A
qPCR primer: mCherry-P2A (A3H) rev: TAACCACCTCCAGTATGC	This study	N/A
qPCR primer: cMYC fwd: AGTCTGGATCACCTTCTG	This study	N/A
qPCR primer: cMYC rev: AGTCTGGATCACCTTCTG	This study	N/A
qPCR primer: A3B fwd: GAGACACATTCTACGACAAC	This study	N/A
qPCR primer: A3B rev: CAGAACCAAGAGAGGAAG	This study	N/A
qPCR primer: GAPDH fwd: GAAGGTGAAGGTCGGAGTC	This study	N/A
qPCR primer: GAPDH rev: GAAGATGGTGATGGGATTTC	This study	N/A
mutREAD adapter: mutREAD-i5-lower_1: /5Phos/TC GCT CAT NNN DAG ATC GGA AGA GCG TCG TGT AGG GAA AGA GTG T	This study	N/A
mutREAD adapter: mutREAD-i5-upper_1: CGC TCT TCC GAT CTH NNN ATG AGC GAT GCA /3Phos/	This study	N/A
mutREAD adapter: mutREAD-i7-lower_1: AAT TGG TAC ACG NNN DAG ATC GGA AGA GCA	This study	N/A
mutREAD adapter: mutREAD-i7-upper_1: GTG ACT GGA GTT CAG ACG TGT GCT CTT CCG ATC THN NNC GTG TAC C	This study	N/A
sgRNA: AAVS1_1: CTGTGCCCCGATGCACAC	This study	N/A
sgRNA: AAVS1_2: GGCGCGTCGCTCGCTCGCTC	This study	N/A
sgRNA: UBR4_1: GGAACCGATTGATATAGCGT	This study	N/A
sgRNA: UBR4_2: GACTGAGGCCACCAGCTG	This study	N/A
sgRNA: UBR5_1: GTTCCAATACATTCAAAG	This study	N/A
sgRNA: UBR5_2: GAAAGGGGTTCTACCAGCA	This study	N/A
sgRNA: HUWE1_1: GTAGCCGAGTTAGCAGCG	This study	N/A
sgRNA: HUWE1_2: GAGATTCCATGATTTCAGAA	This study	N/A
sgRNA: UNG2_1: ATGGACCTAATCAAGCTCAC	This study	N/A
sgRNA: UNG2_2: AGCTGGAAGAAGCACCTCAG	This study	N/A
Seq primer: sgDeepSeq_rev_TGAG: CTCTTTCCCTACACGACGCTCTTCCGATCTNNNNNN CTCATTCCAGCATAGCTCTTAAAC	This study	N/A
Seq primer: sgDeepSeq_rev_TCGA: CTCTTTCCCTACACGACGCTCTTCCGATCTNNNNNN TCGATTCCAGCATAGCTCTTAAAC	This study	N/A
Seq primer: sgDeepSeq_rev_TAGG: CTCTTTCCCTACACGACGCTCTTCCGATCTNNNNNN CCTATTCCAGCATAGCTCTTAAAC	This study	N/A

Seq primer: sgDeepSeq_rev_GTTC: CTCTTTCCCTACACGACGCTCTTCCGATCTNNNNNN GAACTTCCAGCATAGCTCTTAAAC	This study	N/A
Seq primer: sgDeepSeq_rev_GGAT: CTCTTTCCCTACACGACGCTCTTCCGATCTNNNNNN ATCCTTCCAGCATAGCTCTTAAAC	This study	N/A
Seq primer: sgDeepSeq_rev_GAGT: CTCTTTCCCTACACGACGCTCTTCCGATCTNNNNNN ACTCTTCCAGCATAGCTCTTAAAC	This study	N/A
Seq primer: sgDeepSeq_rev_GAAG: CTCTTTCCCTACACGACGCTCTTCCGATCTNNNNNN CTTCTTCCAGCATAGCTCTTAAAC	This study	N/A
Seq primer: sgDeepSeq_rev_CTTG: CTCTTTCCCTACACGACGCTCTTCCGATCTNNNNNN CAAGTTCCAGCATAGCTCTTAAAC	This study	N/A
Seq primer: sgDeepSeq_rev_CTCA: CTCTTTCCCTACACGACGCTCTTCCGATCTNNNNNN TGAGTTCCAGCATAGCTCTTAAAC	This study	N/A
Seq primer: sgDeepSeq_rev_CGAA: CTCTTTCCCTACACGACGCTCTTCCGATCTNNNNNN TTCGTTCCAGCATAGCTCTTAAAC	This study	N/A
Seq primer: sgDeepSeq_rev_CCTA: CTCTTTCCCTACACGACGCTCTTCCGATCTNNNNNN TAGGTTCCAGCATAGCTCTTAAAC	This study	N/A
Seq primer: Fwd1_hybrid_P7_Nras: GCATACGAGATAGCTAGCCACC	This study	N/A
Seq primer: Rev2_p5_sgDeepSeq: AATGATACGGCGACCACCGAGATCTACACTCTTTCC CTACACGACGCT	This study	N/A
Seq primer: Fwd2_p7_sgDeepSeq: CAAGCAGAAGACGGCATACGAGATAGCTAGCCACC	This study	N/A
NEBNext® Multiplex Oligos for Illumina® (96 Unique Dual Index Primer Pairs), Set 1 and 2	New England Biolabs	Cat#: E6440
**Recombinant DNA**		
pRRL-TRE3G-Cas9-P2A-GFP-PGK-IRES-rtTA3	Johannes Zuber, IMP	N/A
DualCRISPR-hU6-sgRNA-mU6-sgRNA-EF1as-iRFP	de Almeida M., Hinterndorfer M. *et al*., 2021	N/A
DualCRISPR-hU6-sgRNA-mU6-sgRNA-EF1as-BFP	de Almeida M., Hinterndorfer M. *et al*., 2021	N/A
DualCRISPR-hU6-sgRNA-mU6-sgRNA-EF1as-Thy1.1-P2A-NeoR	de Almeida M., Hinterndorfer M. *et al*., 2021	N/A
PRRL-PBS-U6-sgRNA-EF1as-Thy1-P2A-NeoR (sgETN)	Johannes Zuber, IMP	N/A
pLX303-SFFV-MYC-mCherry-P2A-3xHA-A3A	This study	N/A
pTR600-A3B-3xHA	Ooms M., *et al*., 2012	N/A
pLX303-SFFV-MCS	This study	N/A
pLX303-SFFV-MYC-mCherry-P2A-3xHA-A3C	This study	N/A
pLX303-SFFV-MYC-mCherry-P2A-3xHA-A3D	This study	N/A
pLX303-SFFV-MYC-mCherry-P2A-3xHA-A3F	This study	N/A
pLX303-SFFV-MYC-mCherry-P2A-3xHA-A3G	This study	N/A
pLX303-SFFV-MYC-mCherry-P2A-3xHA-A3H-I	This study	N/A
pLX303-SFFV-MYC-mCherry-P2A-3xHA-A3H-II	This study	N/A
pLX303-SFFV-MYC-A3H-I	This study	N/A
pLX303-SFFV-MYC-A3H-II	This study	N/A
pLX303-SFFV-Gaussia luciferase-MYC	This study	N/A
pLX303-SFFV-MYC-mCherry-P2A-OLLAS-A3H-I	This study	N/A
pLX303-SFFV-MYC-mCherry-P2A-OLLAS-A3H-II	This study	N/A
pLX303-SFFV-MYC-mCherry-P2A-OLLAS-A3H-I-K117R/K121R	This study	N/A

pLX303-SFFV-MYC-mCherry-P2A-OLLAS-A3H-I-K161R/K168R/K174R/K181R	This study	N/A
pLX303-SFFV-MYC-mCherry-P2A-OLLAS-A3H-I-K117R/K121R/K161R/K168R/K174R/K181R	This study	N/A
pLX303-SFFV-MYC-mCherry-P2A-OLLAS-A3H-I-K16R/K27R/K117R/ K121R/K161R/K168R/K174R/K181R	This study	N/A
pLX303-SFFV-MYC-mCherry-P2A-OLLAS-A3H-I-K50R/K51R/K52R	This study	N/A
pLX303-SFFV-MYC-mCherry-P2A-OLLAS-A3H-I-K27R/K50R/K51R/K52R/K64R/K97R/K121R/K153R	This study	N/A
pLX303-SFFV-MYC-mCherry-P2A-OLLAS-A3H-I-all K to R	This study	N/A
pLX303-SFFV-MYC-mCherry-A3H-II-P2A-OLLAS-EGFP-A3H-I	This study	N/A
pCW-MYC-TurboID-MCS-PGK-mCherry-P2A-rtTA	Scinicariello S., *et al*., 2023	N/A
pCW-MYC-TurboID-A3H-I-PGK-mCherry-P2A-rtTA	This study	N/A
pCW-MYC-TurboID-A3H-II-PGK-mCherry-P2A-rtTA	This study	N/A
pCW-MYC-TurboID-GFP-PGK-mCherry-P2A-rtTA	This study	N/A
pLX303-SFFV-MYC-mCherry-P2A-OLLAS-EGFP-A3H-I	This study	N/A
pLX303-SFFV-MYC-mCherry-P2A-OLLAS-EGFP-A3H-II	This study	N/A
pLX303-SFFV-MYC-mCherry-P2A-OLLAS-EGFP-A3H-II-W155A	This study	N/A
pLX303-SFFV-MYC-mCherry-P2A-OLLAS-EGFP-A3H-II-R175E/R176E	This study	N/A
pLX303-SFFV-MYC-mCherry-P2A-OLLAS-EGFP-A3H-I-G105R	This study	N/A
pLX303-SFFV-MYC-mCherry-P2A-OLLAS-EGFP-A3H-II-R105G	This study	N/A
pLX303-SFFV-MYC-mCherry-P2A-3xHA-A3H-I	This study	N/A
pLX303-SFFV-MYC-mCherry-P2A-3xHA-A3H-II	This study	N/A
pLX303-SFFV-MYC-mCherry-P2A-3xHA-A3H-II-W155A	This study	N/A
pLX303-SFFV-MYC-mCherry-P2A-3xHA-A3H-II-R175E/R176E	This study	N/A
pLX303-SFFV-MYC-mCherry-P2A-3xHA-A3H-I-G105R	This study	N/A
pLX303-SFFV-MYC-mCherry-P2A-3xHA-A3H-II-R105G	This study	N/A
pLX303-SFFV-MYC-mCherry-P2A-3xHA-A3G-F126Y/W127S/K180A/I183A/L184A/I187A	This study	N/A
pLX303-SFFV-MYC-mCherry-P2A-3xHA-A3G-F126Y/W127S/K180S/L184S	This study	N/A
pLX303-SFFV-MYC-mCherry-P2A-3xHA-A3G-Y181A/Y182A	This study	N/A
pET-47b(+)-6his-3C-MCS	Elif Karagöz, Max Perutz Labs, Medical University of Vienna	N/A
pET-47b(+)-10his-MBP-3C-MCS	This study	N/A
pET-47b(+)-10his-MBP-3C-A3H-I	This study	N/A
pET-47b(+)-10his-MBP-3C-A3H-II	This study	N/A
pET-47b(+)-10his-MBP-3C-A3H-II-E56A/W115A/R175E/R176E	This study	N/A
pLX303-MYC-mCherry-P2A-OLLAS-EGFP-A3H-II-E56A/W155A/R175E/R176E	This study	N/A
**Software and algorithms**		
GraphPad Prism 10.0.2	GraphPad Software Inc.	RRID:SCR_002798
R (v4.0.2) programming environment using RStudio (v4.1.3)	R	RRID:SCR_001905
FlowJo 10.7.1	FlowJo	RRID:SCR_008520
ImageJ 1.5.4	ImageJ	RRID:SCR_003070
**Other**		

Luna Universal qPCR Master Mix	New England Biolabs	Cat# M3003S
Pierce™ Protein A/G Magnetic Beads	Thermo Fisher Scientific	Cat# 88803
NuPAGE™ 3 to 8%, Tris-Acetate, 1.0–1.5 mm, Mini Protein Gels	Invitrogen	Cat# EA03785BOX
Pierce™ Streptavidin Magnetic Beads	Thermo Fisher Scientific	Cat# 88816
Anti-MBP Magnetic Beads	New England Biolabs	Cat# E8037S
VAHTS DNA Clean Beads	Vazyme	Cat# N411
4–20% Mini-PROTEAN® TGX Stain-Free™ Protein Gels	BioRad	Cat# 4568094

Supplementary Figures

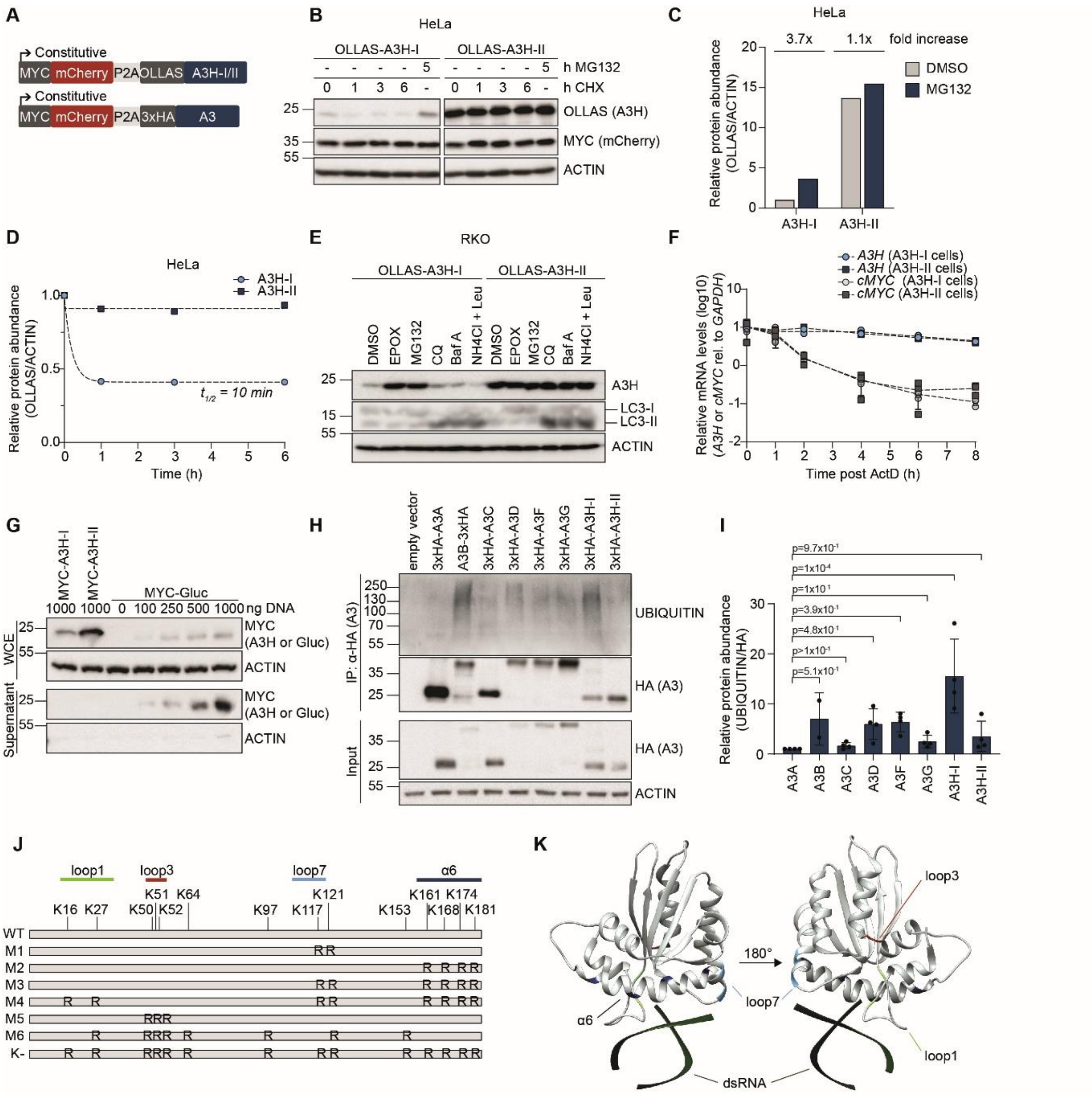

Supplementary Figure 1. Proteasomal degradation controls protein levels of cancer-associated A3s. **(a)** Schematic representation of the lentiviral mCherry-P2A-OLLAS-A3 and mCherry-P2A-3xHA-A3 expression constructs used in this study. The ribosomal skip site P2A ensures equimolar translation of the stable internal control mCherry and tagged A3 protein. **(b-d)** Lentiviral constructs encoding mCherry-P2A-OLLAS-A3H-I/II were stably integrated in HeLa cells by lentiviral transduction. Polyclonal cell pools were treated with MG132 or CHX for the indicated times, followed by **(b)** analysis of protein levels by WB, **(c)** quantification of relative A3H-I and A3H-II protein levels upon proteasome inhibition, or **(d)** translation inhibition, by calculating single-step exponential decay curves to derive protein half-live. **(e)** RKO cells expressing the indicated OLLAS-tagged-A3H haplotypes were treated with different proteasome inhibitors (EPOX, MG132), or autophagy/lysosomal degradation inhibitors (chloroquine (CQ)), bafilomycin A (BafA), or leupeptin (Leu)) for 5 h. LC3-I to LC3-II conversion was detected as a marker for inhibition of lysosomal degradation. **(f)** RKO cells stably expressing OLLAS-A3H were treated for the indicated times with Actinomycin D (ActD), after which relative exogenous *APOBEC3H* and endogenous *cMYC* mRNA levels were quantified by RT-qPCR. **(g)** HEK-293T cells were transfected with plasmids encoding MYC-tagged A3H-I/II or Gaussia luciferase (MYC-Gluc). 48 h. post transfection, MYC-tagged protein levels in the supernatant and the whole cell extract (WCE) were analyzed by WB. **(h-i)** HEK-293T cells were transfected with different amounts of the indicated 3xHA-A3 plasmids to achieve similar steady-state A3 protein levels. After treatment with EPOX for 5 h., 3xHA-tagged proteins were immunoprecipitated from cell lysates, and

their ubiquitination analyzed by **(h)** WB with a total ubiquitin antibody, and **(i)** quantified (means and SD, n = 4). **(j)** Schematic representation of lysine positions in A3H-I. Lysine residues were systematically grouped based on their proximity in the A3H structure and mutated to arginine (M1-M6). In K-, all 14 lysine residues were mutated to arginine. **(k)** The positions of the mutated lysine residues in the A3H-II crystal structure are highlighted in different colors (PDB: 6B0B). Source data are provided as a Source Data file.

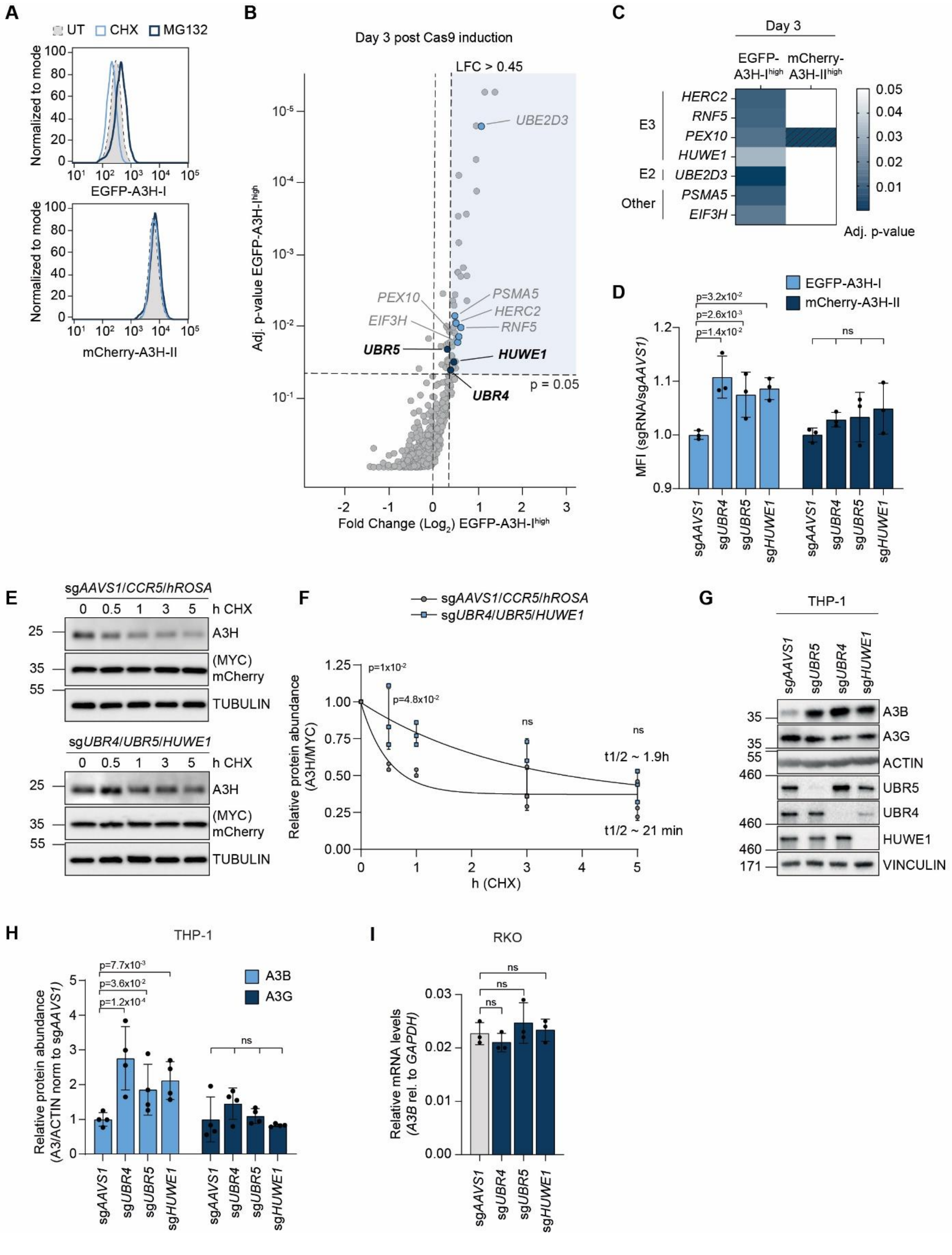

Supplementary Figure 2. The E3 ligases UBR4, UBR5, and HUWE1 independently mediate turnover of A3B and A3H-I. (a) Monoclonal RKO-DOX-Cas9-dualA3H cells were treated with CHX or EPOX for 5 h., followed by analysis of EGFP-A3H-I or mCherry-A3H-II levels by flow cytometry. **(b)** Targeted genes enriched in EGFP-A3H-I^{high} sorted cell populations 3 days post Cas9 induction. **(c)** Heatmap of top genes on log_2 fold-change and p-value grouped by functional categories. Genes enriched in EGFP-A3H-I^{high} cell populations 3 days post Cas9 induction with a log_2 fold-change > 0.45 which were not enriched in mCherry^{high} or GFP^{low} on the same day. Dashed lines indicate a log_2 fold-change < 0.45. Adjusted p-values are based on MaGECK analysis of three independent replicate sorts. E3 (E3 ligases), E2 (E2 conjungating enzymes). **(d)** Polyclonal RKO-DOX-Cas9-dualA3H cells were transduced with sgRNA vectors targeting *UBR4*, *UBR5*, or *HUWE1*. EGFP-A3H-I and mCherry-A3H-II abundance was analyzed by flow cytometry 6 days after Cas9 induction.

The mean fluorescence intensity (MFI) was quantified (2-way ANOVA, corrected for multiple comparisons using the Dunnett method, n = 3, means and SD). **(e)** RKO-DOX-Cas9-MYC-mCherry-P2A-3xHA-A3H-I cells were transduced with sgRNA simultaneously targeting either the three E3 ligases or control loci (*AAVS1*, *CCR5*, *hROSA*), gene editing induced with DOX for 6 days and then treated with CHX for different times. A3H-I protein levels were determined by WB and **(f)** the half-life quantified from single-step exponential decay curves, statistics were calculated by 2-way ANOVA, corrected for multiple comparisons using the Šídák method, (n = 3). Data represent mean and SD. **(g)** THP-1 cells harboring DOX-inducible Cas9 were transduced with sgRNAs targeting *UBR4*, *UBR5*, or *HUWE1*, and sorted for sgRNA-positive cells. Gene editing was induced with DOX for 3 days, after which endogenous A3B and A3G protein levels were determined by WB, and **(h)** quantified (means and SD, 2-way ANOVA, not corrected for multiple comparisons, ns: $p \geq 0.05$, n = 4). **(i)** RKO cells harboring DOX-inducible Cas9 were transduced with sgRNAs targeting *UBR4*, *UBR5*, or *HUWE1*, and sorted for sgRNA-positive cells. Gene editing was induced with DOX for 6 days, after which endogenous *A3B* mRNA levels were determined by RT-qPCR (means and SD, one-way ANOVA, ns: $p \geq 0.05$, n = 3). Source data are provided as a Source Data file.

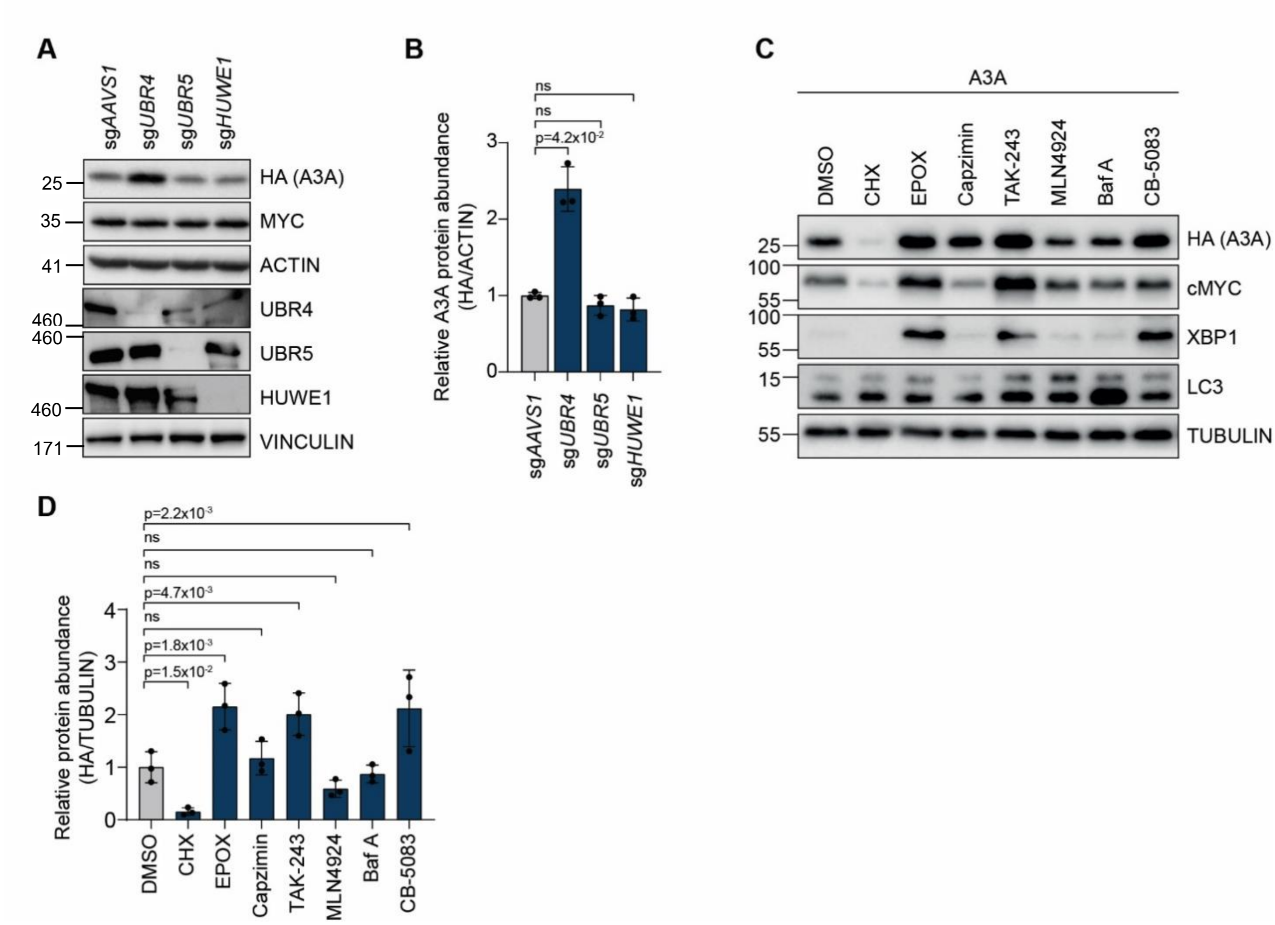

Supplementary Figure 3. The E3 ligases UBR4, UBR5, and HUWE1 independently mediate turnover of A3B and A3H-I. (a-b) RKO cells stably expressing 3xHA-A3A were transduced with individual sgRNAs targeting the indicated genes. Subsequently, Cas9 expression was induced by DOX 6 days and 3xHA-A3A levels analyzed by **(a)** WB and **(b)** quantified (means and SD, unpaired t-tests (two-sided), ns: p ≥ 0.05, n = 3). **(c-d)** HEK-293T cells were transfected with a MYC-mCherry-P2A-3xHA-tagged A3A construct. 48 h post transfection, cells were treated with different inhibitors for 5 h and the levels of HA-A3A analyzed by **(c)** WB and **(d)** quantified (means and SD, one-way ANOVA, not corrected for multiple comparisons, ns: p ≥ 0.05, n = 3). Source data are provided as a Source Data file.

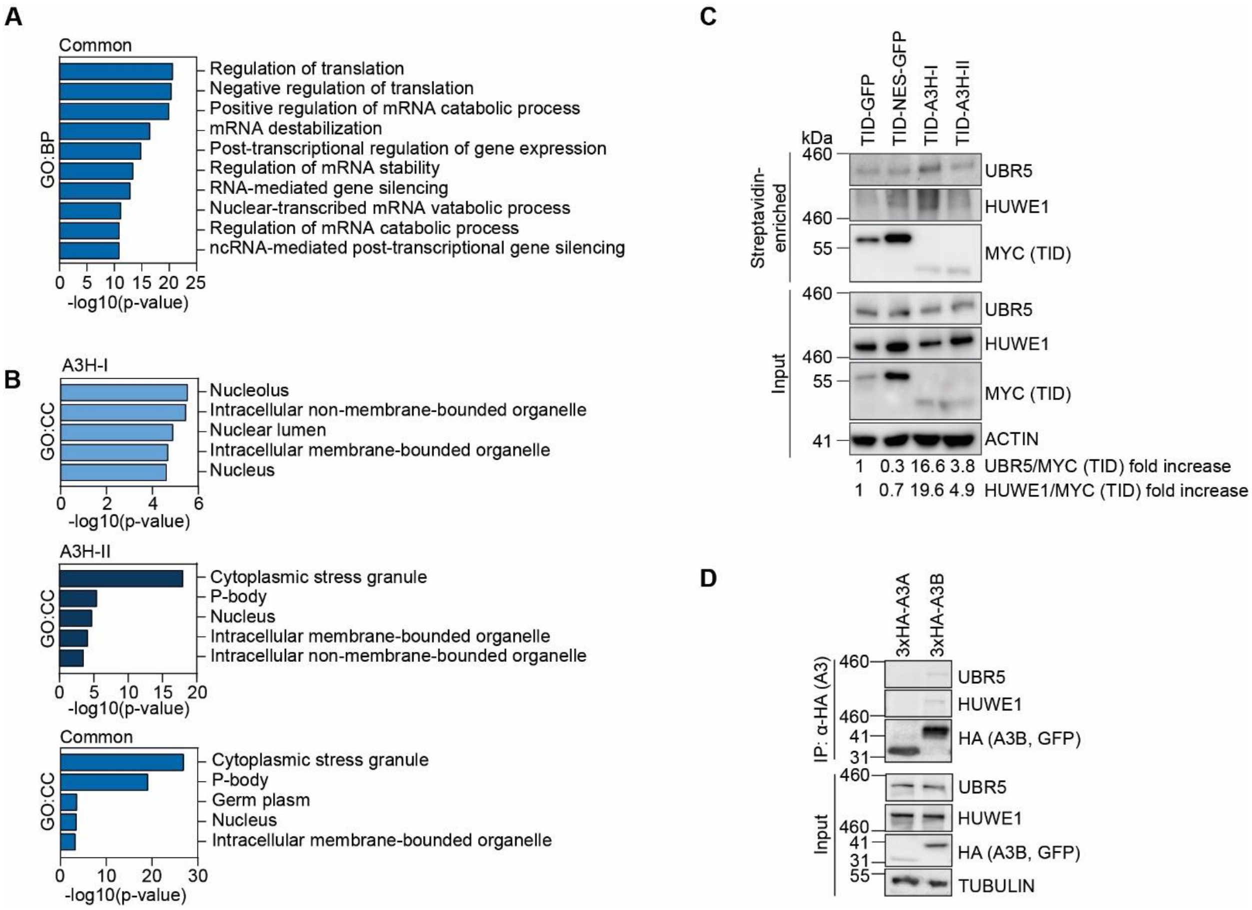

Supplementary Figure 4. UBR5 and HUWE1 form a complex with A3H-I and other unstable A3 deaminases in cells. (a-b) Polyclonal RKO-DOX-TID-A3H-I/II/GFP were treated with different concentrations of DOX for 2 days to achieve similar protein levels. Subsequently, cells were treated with EPOX for 5 h and the culture media supplemented with biotin during the last 15 min. Biotinylated proteins were purified under denaturing conditions and quantified by nLC-MS/MS. Statistical analysis was conducted using moderated t-statistics via the limma-trend method in R and applying the Benjamini–Hochberg multiple testing correction. Data represent biological replicates as mean values +/- SD, n = 3. **(a)** GO terms for biological processes (GO:BP) of differentially enriched proteins shared between A3H-I/GFP and A3H-II/GFP (LFC > 1, p-value < 0.01, input: 121 factors derived from Fig. 3b) **(b)** GO terms for cellular compartments (GO:CC) of differentially enriched proteins in A3H-I/GFP (light blue, input: 170 factors derived from Fig. 3b), A3H-II/GFP (dark blue, input: 52 factors derived from Fig. 3b) and proteins shared between both A3H-I and A3H-II/GFP ("common", medium blue, input: 121 factors derived from Fig. 3b) (LFC > 1, p-value < 0.01). **(c)** Polyclonal RKO-DOX-TID-A3H-I/II/GFP cells were treated with different concentrations of DOX for two days to achieve similar protein levels. Subsequently, cells were treated with EPOX for 5 h., during the last 15 min of which, exogenous biotin was added to the culture media. Biotinylated proteins were purified, and their interaction with endogenous UBR5 and HUWE1 detected by WB. Relative abundance of UBR5/MYC (TID) or HUWE1/MYC(TID) was quantified. Densitometry values are listed. **(d)** HEK-293T cells were transiently transfected with different amounts of plasmids encoding for 3xHA-A3A or catalytically-inactive 3xHA-A3B-E255A to achieve similar steady-state protein levels. Subsequently, 3xHA-tagged proteins were immunoprecipitated, and their interaction with endogenous UBR5 and HUWE1 determined by WB. Source data are provided as a Source Data file.

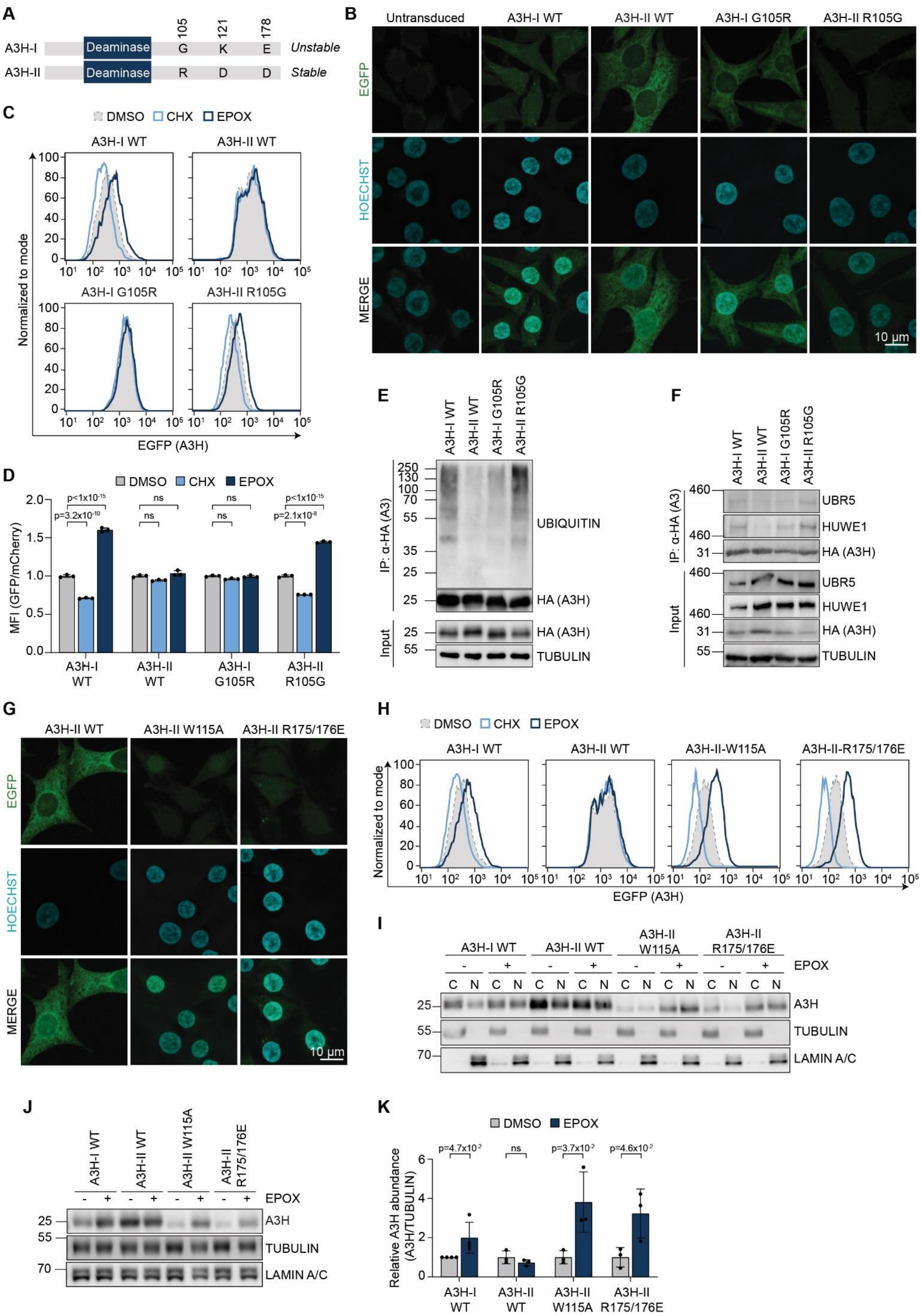
A
105
121
178
A3H-I
Deaminase
G
K
E
Unstable
A3H-II
Deaminase
R
D
D
Stable
B
Untransduced
A3H-I WT
A3H-II WT
A3H-I G105R
A3H-II R105G
EGFP
HOECHST
MERGE
10 µm
C
DMSO
CHX
EPOX
A3H-I WT
A3H-II WT
A3H-I G105R
A3H-II R105G
Normalized to mode
EGFP (A3H)
D
DMSO
CHX
EPOX
MFI (GFP/mCherry)
p<1x10-15
p=3.2x10-10
ns
p=2.1x10-8
A3H-I WT
A3H-II WT
A3H-I G105R
A3H-II R105G
E
A3H-I WT
A3H-II WT
A3H-I G105R
A3H-II R105G
IP: α-HA (A3)
UBIQUITIN
HA (A3H)
Input
TUBULIN
F
A3H-I WT
A3H-II WT
A3H-I G105R
A3H-II R105G
IP: α-HA (A3)
UBR5
HUWE1
HA (A3H)
Input
TUBULIN
G
A3H-II WT
A3H-II W115A
A3H-II R175/176E
EGFP
HOECHST
MERGE
10 µm
H
DMSO
CHX
EPOX
A3H-I WT
A3H-II WT
A3H-II-W115A
A3H-II-R175/176E
Normalized to mode
EGFP (A3H)
I
A3H-I WT
A3H-II WT
A3H-II W115A
A3H-II R175/176E
EPOX
C
N
A3H
TUBULIN
LAMIN A/C
J
A3H-I WT
A3H-II WT
A3H-II W115A
A3H-II R175/176E
EPOX
A3H
TUBULIN
LAMIN A/C
K
DMSO
EPOX
p=4.7x10-2
ns
p=3.7x10-2
p=4.6x10-2
Relative A3H abundance (A3H/TUBULIN)
A3H-I WT
A3H-II WT
A3H-II W115A
A3H-II R175/176E

Supplementary Figure 5. RNA binding protects A3s from E3 ligase binding and ubiquitination, thereby promoting their stability in cells. (a) Schematic overview of the three differential amino acid positions in A3H-I and A3H-II. **(b)** Confocal microscopy of RKO-mCherry-P2A-EGFP-A3H-I/II/I-G105R/II-R105G cells. Maximum intensity projections of 11 slices are displayed (scale bar = 10 µm). **(c-d)** RKO-mCherry-P2A-EGFP-A3H cells expressing the indicated EGFP-tagged A3H variants were treated for 5 h. with EPOX or CHX, followed by measurement of the mean fluorescence intensity (MFI) of EGFP-A3H by flow cytometry. **(d)** Quantification of (c) (means and SD, 2-way ANOVA, corrected for multiple comparisons using the Tukey method, ns: $p \geq 0.05$, $n = 3$). **(e-f)** Different amounts of the indicated 3xHA-A3H plasmids were transiently transfected in HEK-293T cells to achieve similar steady-state A3 protein levels, followed by 5 h. of EPOX treatment and immunoprecipitation of 3xHA-tagged proteins from the cell lysates. Their **(e)** ubiquitination or **(f)** interaction with UBR5 and HUWE1 was analyzed by WB. **(g)** Confocal microscopy of RKO-mCherry-P2A-EGFP-A3H cells expressing the indicated EGFP-tagged A3H variants. Maximum intensity projections of 11 slices are displayed (scale bar = 10 µm). **(h)** FACS plots corresponding to Figure 4a. RKO-mCherry-P2A-EGFP-A3H cells expressing the indicated EGFP-tagged A3H variants were treated for 5 h. with EPOX or CHX, after which mCherry and EGFP-A3H abundances were measured by flow cytometry. **(i)** HEK-293T cells were transfected with different amounts of plasmids expressing the indicated 3xHA-tagged A3H constructs to achieve similar steady-state protein levels. Following 5 h. of EPOX treatment, whole cell extracts (Fig. S4j), cytoplasmic (C) and nuclear (N) fractions were extracted, analyzed by WB. **(j)** Whole cell extract samples corresponding to (i) and Fig. 4c. **(k)** Quantification of (j) (means and SD, multiple unpaired t-tests (two-sided), not corrected for multiple comparisons, ns: $p \geq 0.05$, $n = 3$). Source data are provided as a Source Data file.

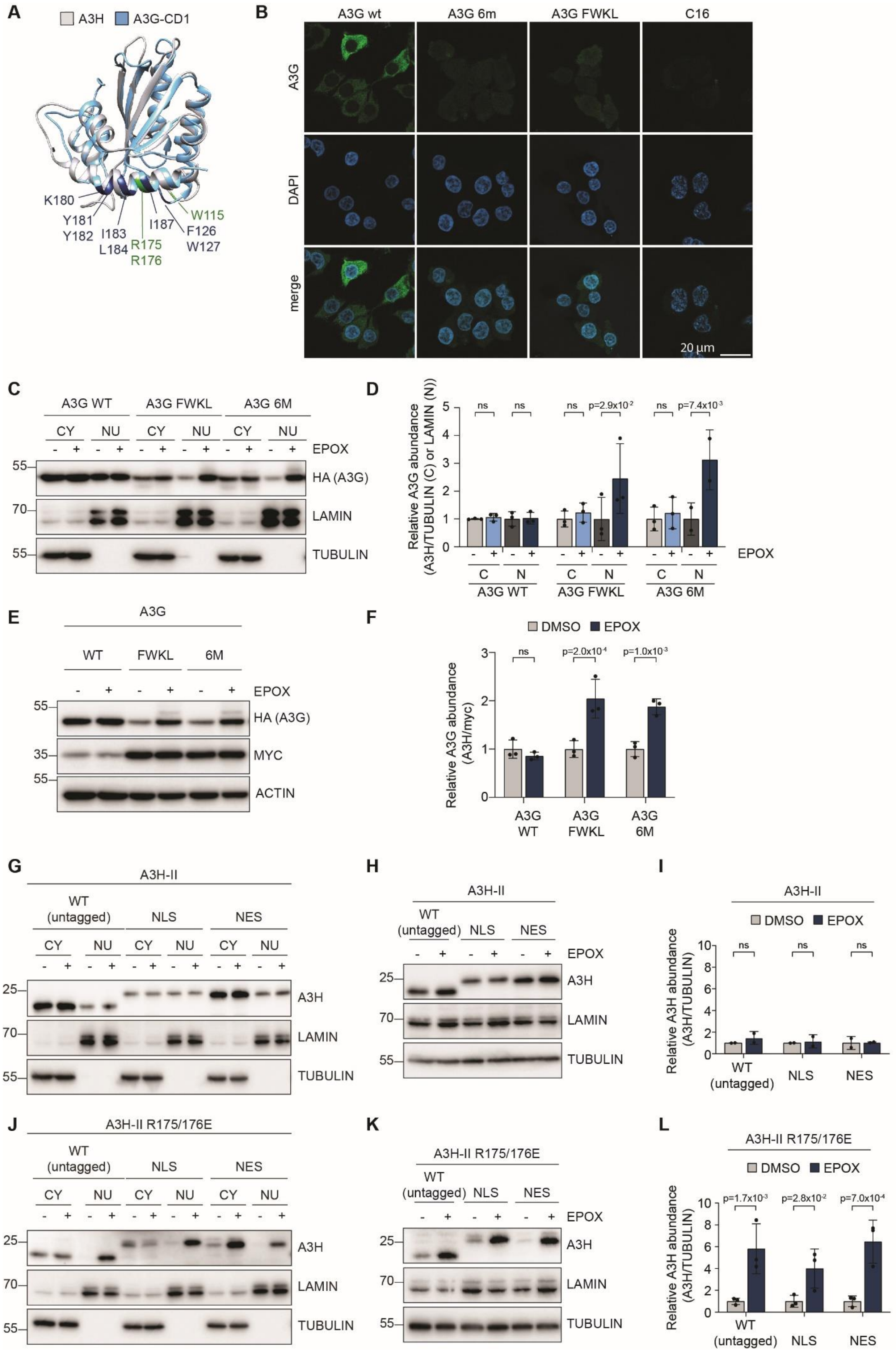
A
A3H
A3G-CD1
K180
Y181
Y182
I183
L184
R175
R176
I187
W115
F126
W127
B
A3G wt
A3G 6m
A3G FWKL
C16
A3G
DAPI
merge
20 µm
C
A3G WT
A3G FWKL
A3G 6M
CY
NU
EPOX
HA (A3G)
LAMIN
TUBULIN
D
Relative A3G abundance (A3H/TUBULIN (C) or LAMIN (N))
ns
p=2.9x10-2
p=7.4x10-3
A3G WT
A3G FWKL
A3G 6M
E
A3G
WT
FWKL
6M
EPOX
HA (A3G)
MYC
ACTIN
F
DMSO
EPOX
Relative A3G abundance (A3H/myc)
ns
p=2.0x10-4
p=1.0x10-3
A3G WT
A3G FWKL
A3G 6M
G
A3H-II
WT (untagged)
NLS
NES
CY
NU
A3H
LAMIN
TUBULIN
H
A3H-II
WT (untagged)
NLS
NES
EPOX
A3H
LAMIN
TUBULIN
I
A3H-II
DMSO
EPOX
Relative A3H abundance (A3H/TUBULIN)
ns
WT (untagged)
NLS
NES
J
A3H-II R175/176E
WT (untagged)
NLS
NES
CY
NU
A3H
LAMIN
TUBULIN
K
A3H-II R175/176E
WT (untagged)
NLS
NES
EPOX
A3H
LAMIN
TUBULIN
L
A3H-II R175/176E
DMSO
EPOX
Relative A3H abundance (A3H/TUBULIN)
p=1.7x10-3
p=2.8x10-2
p=7.0x10-4
WT (untagged)
NLS
NES

Supplementary Figure 6. RNA binding protects A3s from E3 ligase binding and ubiquitination, thereby promoting their stability in cells. (a) Structural alignment of A3H-II (PDB:6B0B) and A3G-CD1 (PDB: 5K81). Relevant amino acid residues mutated in the various RNA-binding mutants are colored in green (A3H-II) or dark blue (A3G). **(b)** Confocal microscopy of RKO-mCherry-P2A-EGFP-A3G wt and mutant cells. (scale bar = 20 µm). **(c-f)** HEK-293T cells were transfected with different amounts of plasmids expressing the indicated 3xHA-tagged A3G constructs to achieve similar steady-state protein levels. Following 5 h. of EPOX treatment, cytoplasmic (C) and nuclear (N) fractions were extracted, analyzed by **(c)** WB and **(d)** quantified (means and SD, 2-way ANOVA, corrected for multiple comparisons using the Tukey method, ns: $p \geq 0.05$, $n = 3$). **(e)** WB and **(f)** quantification (means and SD, 2-way ANOVA, corrected for multiple comparisons using the Šídák method, ns: $p \geq 0.05$, $n = 3$) of whole cell extract samples corresponding to (c-d). **(g-i)** HEK-293T cells were transfected with different amounts of plasmids expressing the indicated 3xHA-tagged A3H-II constructs to achieve similar steady-state protein levels. Following 5 h. of EPOX treatment, cytoplasmic (C) and nuclear (N) fractions were extracted and analyzed by **(g)** WB. **(h)** WB and **(i)** quantification (2-way ANOVA, corrected for multiple comparisons using the Šídák method, ns: $p \geq 0.05$, $n = 3$) of whole cell extract samples corresponding to (g) and Fig. 4j. **(j-l)** HEK-293T cells were transfected with different amounts of plasmids expressing the indicated 3xHA-tagged A3H-II R175/176E constructs to achieve similar steady-state protein levels. Following 5 h. of EPOX treatment, cytoplasmic (C) and nuclear (N) fractions were extracted and analyzed by **(j)** WB. **(k)** WB and **(l)** quantification (2-way ANOVA, not corrected for multiple comparisons, ns: $p \geq 0.05$, $n = 3$) of whole cell extract samples corresponding to (j) and Fig. 4k. Source data are provided as a Source Data file.

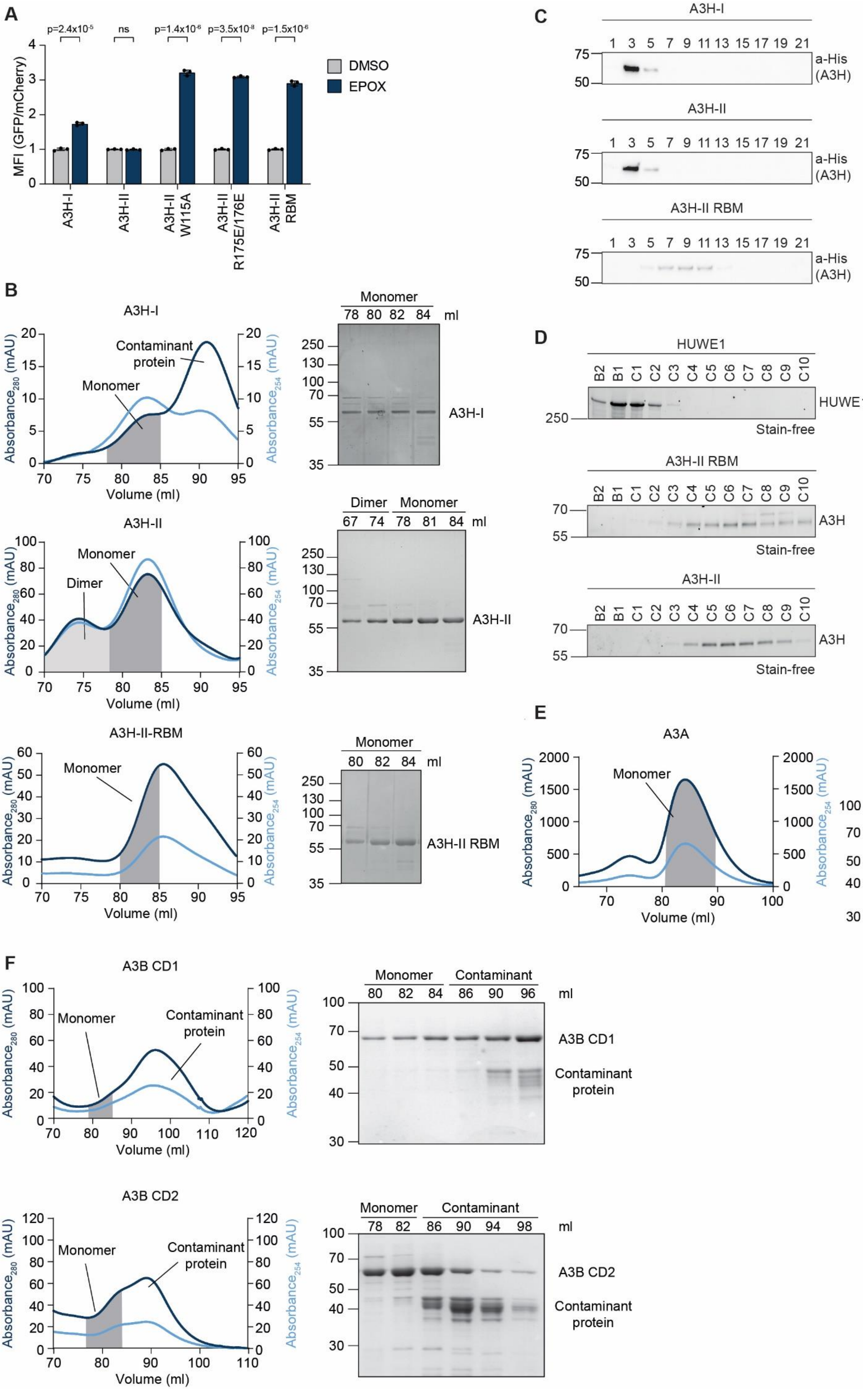
A
p=2.4x10-5
ns
p=1.4x10-6
p=3.5x10-8
p=1.5x10-6
MFI (GFP/mCherry)
DMSO
EPOX
A3H-I
A3H-II
A3H-II W115A
A3H-II R175E/176E
A3H-II RBM
B
A3H-I
Contaminant protein
Monomer
Absorbance280 (mAU)
Absorbance254 (mAU)
Volume (ml)
Monomer
78 80 82 84 ml
A3H-I
A3H-II
Dimer
Monomer
Dimer
Monomer
67 74 78 81 84 ml
A3H-II
A3H-II-RBM
Monomer
Monomer
80 82 84 ml
A3H-II RBM
C
A3H-I
A3H-II
A3H-II RBM
1 3 5 7 9 11 13 15 17 19 21
a-His (A3H)
D
HUWE1
B2 B1 C1 C2 C3 C4 C5 C6 C7 C8 C9 C10
HUWE1
Stain-free
A3H-II RBM
A3H
A3H-II
E
A3A
Monomer
Monomer
80 82 86 ml
A3A
F
A3B CD1
Monomer
Contaminant protein
Monomer
Contaminant
80 82 84 86 90 96 ml
A3B CD1
Contaminant protein
A3B CD2
Monomer
Contaminant protein
Monomer
Contaminant
78 82 86 90 94 98 ml
A3B CD2
Contaminant protein

Supplementary Figure 7. RNA binding by A3B and A3H proteins prevents their recognition and ubiquitination by E3 ligases in vitro. (a-b) HEK-293T cells were transiently transfected with EGFP-tagged A3H-I/II or A3H-II-RBM. After 48 h., cells were treated with EPOX for 5 h., followed by **(a)** measurement of EGFP-A3H fluorescence by flow cytometry, and quantified (means and SD, multiple unpaired t-tests (two-sided), corrected for multiple comparisons using the Holm-Šídák method, ns: $p \geq 0.05$, $n = 3$). **(b)** SEC profiles of 10x-His-MBP-A3H-I, 10x-His-MBP-A3H-II and 10x-His-MBP-A3H-II-RBM. Dark blue lines depict the absorbance at 280 nm representing eluted protein, light blue lines at 254 nm representing nucleic acid bound to the protein. Monomeric or dimeric fractions were pooled separately and analyzed by Coomassie stained SDS PAGE (right insets). **(c)** Sucrose gradient fractionation pattern of individual recombinant proteins (corresponding to Fig. 5i-j). **(d)** Analytical size exclusion chromatography of individual recombinant proteins, c-terminally tagged hHUWE1 (inactive) or recombinant A3H. **(e-f)** SEC profiles of **(e)** 10x-His-MBP-A3A or **(f)** 10x-His-MBP-A3B-CD1 and 10x-His-MBP-A3B-CD2. Dark blue lines depict the absorbance at 280 nm representing eluted protein, light blue lines at 254 nm representing nucleic acid bound to the protein. Monomeric fractions devoid of contaminant proteins were pooled and analyzed by Coomassie stained SDS PAGE (right insets). Source data are provided as a Source Data file.

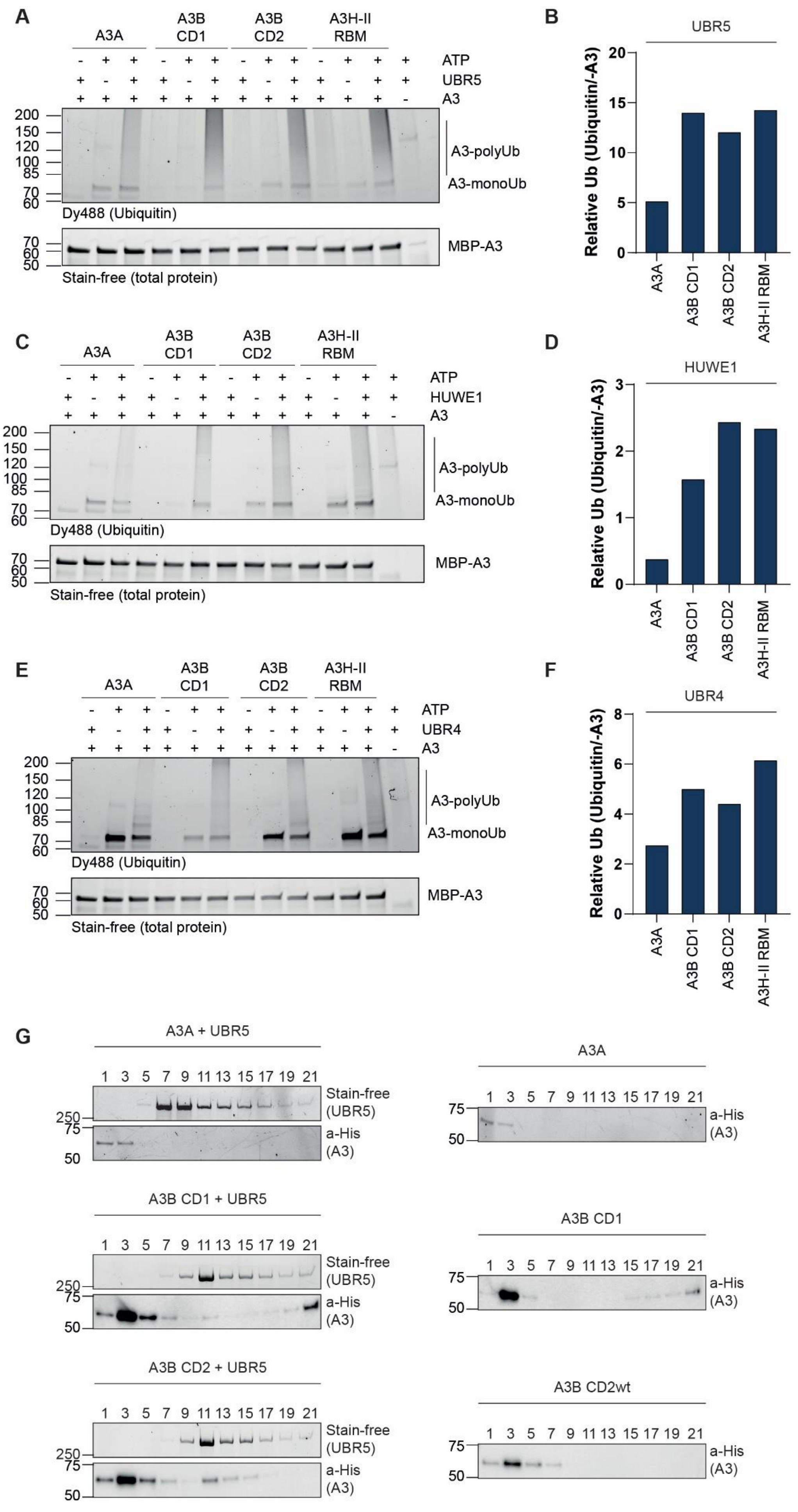
A
A3A
A3B CD1
A3B CD2
A3H-II RBM
ATP
UBR5
A3
200
150
120
100
85
70
60
A3-polyUb
A3-monoUb
Dy488 (Ubiquitin)
MBP-A3
50
Stain-free (total protein)
B
UBR5
Relative Ub (Ubiquitin/-A3)
20
15
10
5
0
C
HUWE1
D
3
2
1
E
UBR4
F
8
6
4
G
A3A + UBR5
1 3 5 7 9 11 13 15 17 19 21
Stain-free (UBR5)
250
75
a-His (A3)
A3B CD1 + UBR5
A3B CD2 + UBR5
A3B CD2wt

Supplementary Figure 8. RNA binding by A3B and A3H proteins prevents their recognition and ubiquitination by E3 ligases in vitro. (a-f) *In vitro* ubiquitination assays were performed with recombinant **(a-b)** UBR5, **(c-d)** HUWE1, or **(e-f)** UBR4 and A3A, A3B-CD1 or A3B-CD2 as substrates, and in the presence of DyLight488-labeled recombinant ubiquitin. Subsequently, A3 proteins were immunoprecipitated using anti-MBP-coupled beads and the ubiquitination pattern visualized by fluorescent imaging for DyLight488. **(g)** Sucrose gradient binding assays of UBR5 and recombinant A3. Source data are provided as a Source Data file.

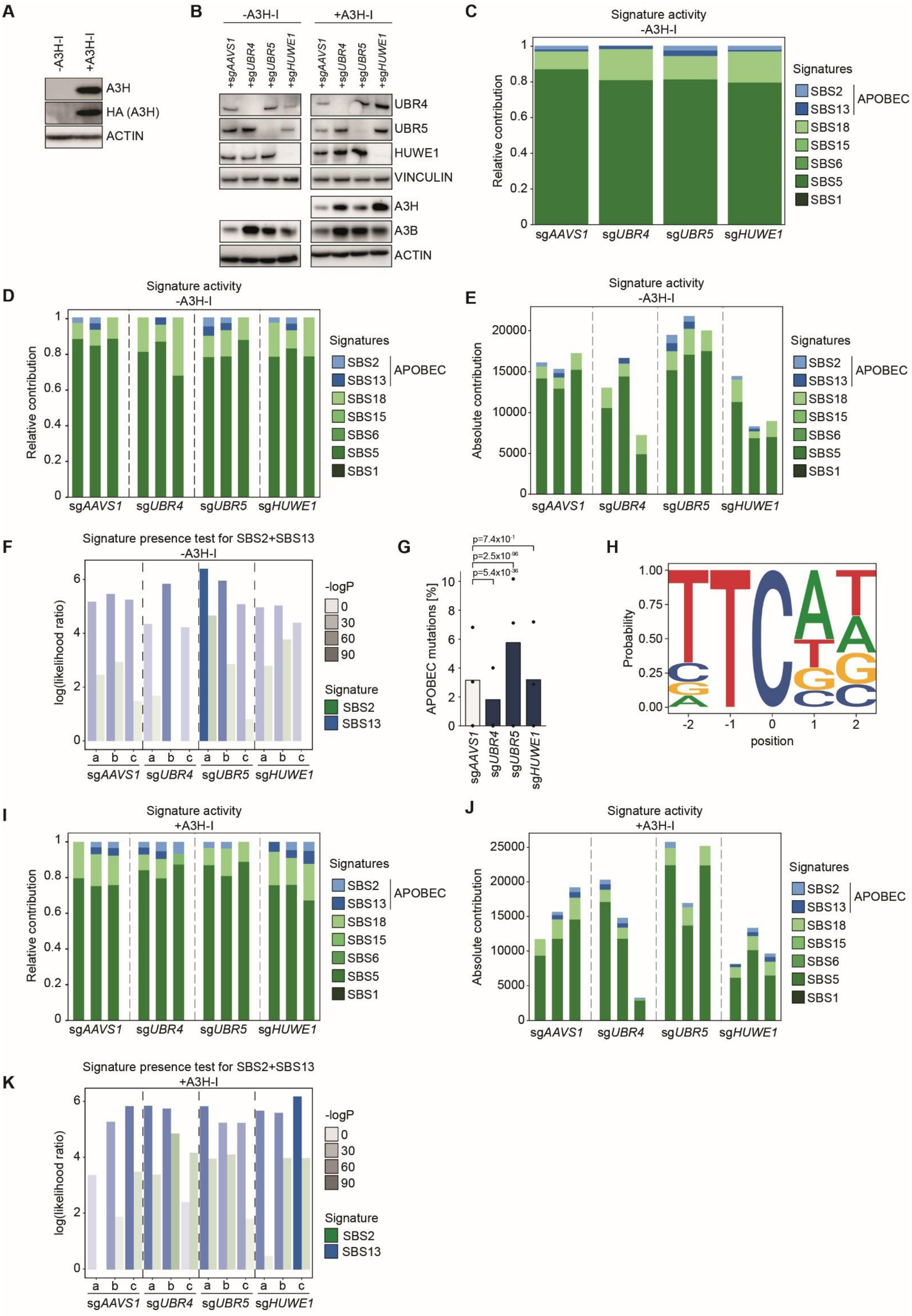
A
-A3H-I
+A3H-I
A3H
HA (A3H)
ACTIN
B
-A3H-I
+A3H-I
+sgAAVS1
+sgUBR4
+sgUBR5
+sgHUWE1
UBR4
UBR5
HUWE1
VINCULIN
A3H
A3B
ACTIN
C
Signature activity
-A3H-I
Relative contribution
sgAAVS1
sgUBR4
sgUBR5
sgHUWE1
Signatures
SBS2
SBS13
APOBEC
SBS18
SBS15
SBS6
SBS5
SBS1
D
Signature activity
-A3H-I
Relative contribution
E
Signature activity
-A3H-I
Absolute contribution
F
Signature presence test for SBS2+SBS13
-A3H-I
log(likelihood ratio)
-logP
Signature
G
p=7.4x10-1
p=2.5x10-96
p=5.4x10-36
APOBEC mutations [%]
H
Probability
position
I
Signature activity
+A3H-I
Relative contribution
J
Signature activity
+A3H-I
Absolute contribution
K
Signature presence test for SBS2+SBS13
+A3H-I
log(likelihood ratio)

Supplementary Figure 9. E3 ligase loss or mutation increases APOBEC signature mutations. (a) Cell lysates from a *UNG2*$^{-/-}$ RKO cell line expressing DOX-inducible Cas9 and mCherry-P2A-3xHA-A3H-I were analyzed by WB for transgene expression. **(b)** Cells with or without exogenous A3H-I expression were transduced with sgRNAs targeting *UBR4*, *UBR5*, or *HUWE1*, and subsequently sorted for sgRNA-positive cells. Gene editing was induced with DOX for up to 10 days, after which the protein levels of the targeted E3 ligases were analyzed by WB. **(c-f)** Best subset signature refitting of cells without A3H-I overexpression, using signatures related to overactivity of APOBEC family enzymes and signatures commonly active in colon carcinoma, the parent tumor type for the model cell line RKO. Each bar represents the signature refitting results, **(c)** averaged per genotype or individual replicates **(d)** scaled to 1 to show the relative signature contribution or **(e)** unscaled to show the absolute signature contribution in terms of number of mutations for individual replicates. **(f)** Signature presence test of samples without A3H-I overexpression for the indicated signatures. Genotypes (n = 3, a-c), are divided by vertical dashed lines. **(g)** Fraction of APOBEC signatures over all identified mutations in samples without A3H-I overexpression. **(h)** Pentanucleotide context preference of control samples without A3H-I overexpression. **(i-k)** Best subset signature refitting of samples with exogenous A3H-I expression, using signatures related to overactivity of APOBEC family enzymes and signatures commonly active in colon carcinoma, the parent tumor type for the model cell line RKO. Each bar represents the signature refitting results, **(i)** scaled to 1 to show the relative signature contribution or **(j)** unscaled to show the absolute signature contribution in terms of number of mutations for individual replicates. **(k)** Signature presence test of samples with exogenous A3H-I expression for the indicated signatures. Genotypes (n = 3, a-c), are divided by vertical dashed lines. The y-axis shows the log of the likelihood ratio, representing the maximum likelihood of the data, given a refit model including the signature of interest over the maximum likelihood of the data, given a refit model excluding the signature of interest. A log(likelihood ratio) greater than 0 indicates a significant activity of the signature of interest. The translucence of the bars (-log(p)) indicates the level of significance of the likelihood ratio test compared to the null hypothesis, which assumes that the mutational profile can be reasonably reconstructed without the signature of interest. Source data are provided as a Source Data file.

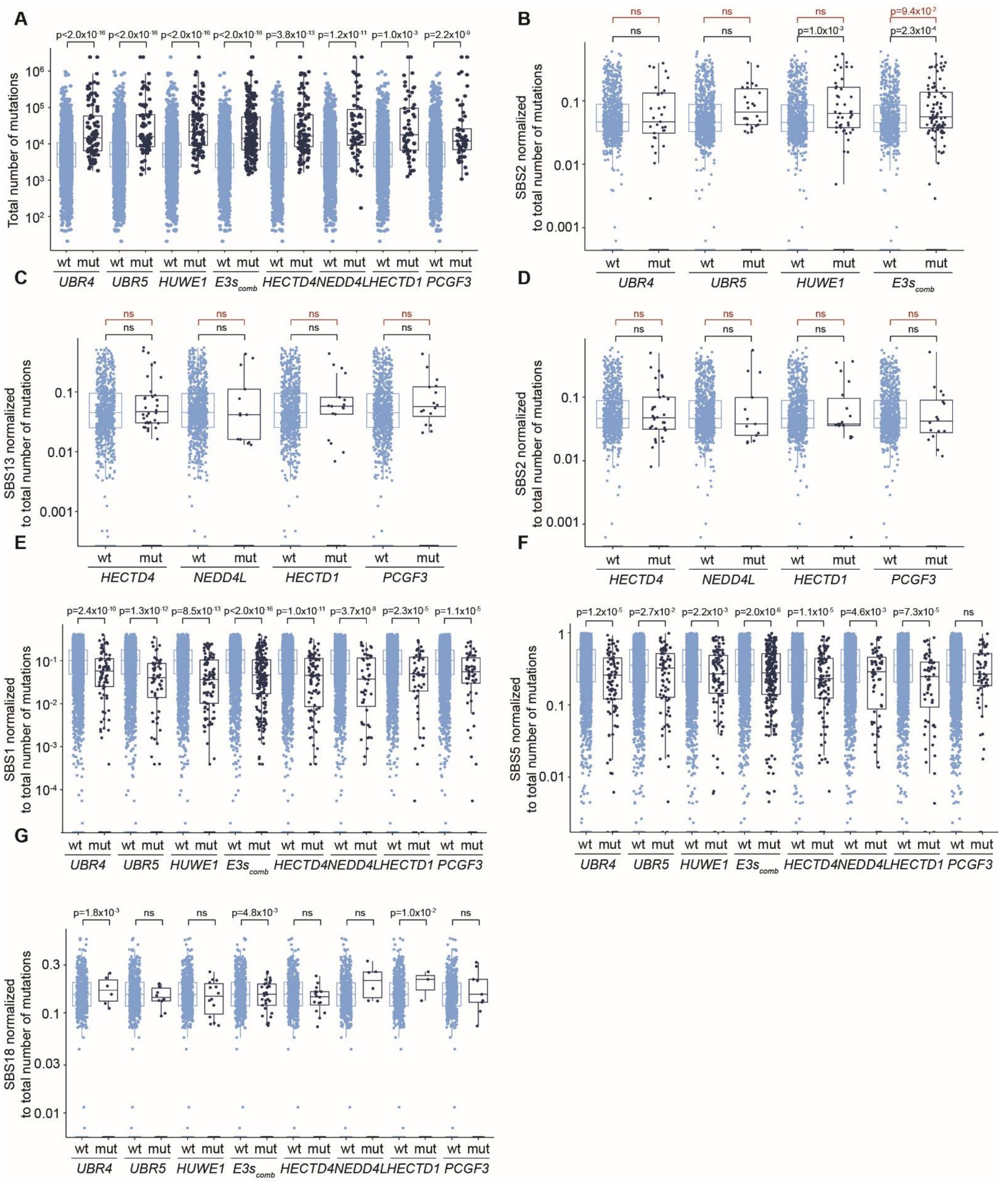

Supplementary Figure 10. E3 ligase loss or mutation increases APOBEC signature mutations. (a-g) Cancers from TCGA/ICGC were grouped, based on whether the indicated E3 ligase genes were wild-type (wt) or mutated (mut), and the **(a)** total number of mutations per group plotted. **(b-g)** Subsequently, mutational signatures were normalized to the total number of mutations in each sample, and the levels of indicated signatures compared between two groups. "E3s$_{comb}$" compares all samples, in which *UBR4*, *UBR5* and *HUWE1* are either all wild-type (wt), or at least one of the E3 ligase genes was mutated (mut). Data represent Wilcox rank sum test (two-sided) between wt and mut of each genotype, ns: p > 0.05, n = 2703 cancer genome samples). Box plots show the median (centre line), interquartile range (25th-75th

percentiles; box), and whiskers extending to the minimum and maximum values within 1.5x the interquartile range.
