# Supplementary material for: Guardian ubiquitin E3 ligases target cancer-associated APOBEC3 deaminases for degradation to promote human genome integrity
Source: Nat Commun. 2026 Jan 19;17:1723. doi: 10.1038/s41467-026-68420-5 (PMC12913773; doi:10.1038/s41467-026-68420-5)
Supplement: Supplementary file 2 — Description of Additional Supplementary Files [file 41467_2026_68420_MOESM2_ESM.pdf]

Description of Additional Supplementary Files

File Name: Supplementary Data 1

Description: Genetic screen data table.

Values for all genes in all sorted populations at two time points after Cas9 induction. Values reflect LFC and adj. p-values from MaGECK FDR analysis of three independent replicate sorts.

File Name: Supplementary Data 2

Description: Proximity labelling interaction screen data table.

Proteins proximal to the TurboID fusion proteins were covalently labelled by addition of biotin, after which biotinylated proteins were isolated and identified by nLC-MS/MS. Data from n = 3 biological replicates.
