# Supplementary material for: Guardian ubiquitin E3 ligases target cancer-associated APOBEC3 deaminases for degradation to promote human genome integrity
Source: Nat Commun. 2026 Jan 19;17:1723. doi: 10.1038/s41467-026-68420-5 (PMC12913773; doi:10.1038/s41467-026-68420-5)
Supplement: Supplementary file 5 — Reporting Summary [file 41467_2026_68420_MOESM5_ESM.pdf]

nature portfolio

Corresponding author(s): Gijs A. Versteeg

Last updated by author(s): Nov 13, 2025

Reporting Summary

Nature Portfolio wishes to improve the reproducibility of the work that we publish. This form provides structure for consistency and transparency in reporting. For further information on Nature Portfolio policies, see our Editorial Policies and the Editorial Policy Checklist.

Statistics

For all statistical analyses, confirm that the following items are present in the figure legend, table legend, main text, or Methods section.

n/a	Confirmed	
☐	☒	The exact sample size (*n*) for each experimental group/condition, given as a discrete number and unit of measurement
☐	☒	A statement on whether measurements were taken from distinct samples or whether the same sample was measured repeatedly
☐	☒	The statistical test(s) used AND whether they are one- or two-sided<br>*Only common tests should be described solely by name; describe more complex techniques in the Methods section.*
☒	☐	A description of all covariates tested
☒	☐	A description of any assumptions or corrections, such as tests of normality and adjustment for multiple comparisons
☐	☒	A full description of the statistical parameters including central tendency (e.g. means) or other basic estimates (e.g. regression coefficient) AND variation (e.g. standard deviation) or associated estimates of uncertainty (e.g. confidence intervals)
☐	☒	For null hypothesis testing, the test statistic (e.g. *F*, *t*, *r*) with confidence intervals, effect sizes, degrees of freedom and *P* value noted<br>*Give P values as exact values whenever suitable.*
☒	☐	For Bayesian analysis, information on the choice of priors and Markov chain Monte Carlo settings
☒	☐	For hierarchical and complex designs, identification of the appropriate level for tests and full reporting of outcomes
☒	☐	Estimates of effect sizes (e.g. Cohen's *d*, Pearson's *r*), indicating how they were calculated

Our web collection on statistics for biologists contains articles on many of the points above.

Software and code

Policy information about availability of computer code

Data collection	No new software/code was used for new primary data collection.
Data analysis	MS raw data split for each CV using FreeStyle 1.7 (Thermo Fisher), were analyzed using the MaxQuant software package (version 1.6.17.0). MaxQuant output tables were further processed in. R (v4.0.2) programming environment using RStudio (v4.1.3). Statistical analysis on TurboID data was conducted with the limma v.3.54.2 package in R. Genetic screen data were analyzed using MAGeCK 0.5.9.3. GraphPad Prism (v10.2.2) was used for data analysis, statistical testing and single exponential decay curve fitting. BD FACSDiva software (v8.0) and FlowJo 10.7.1 were used for analysis of flow cytometry data. ImageJ 1.5.4 was used for microscopy figure analyses. Data and code pertaining to cancer genome analysis and sequence context analysis can be found on https://github.com/menchelab/apobex and is available under DOI 10.5281/zenodo.17953820.

For manuscripts utilizing custom algorithms or software that are central to the research but not yet described in published literature, software must be made available to editors and reviewers. We strongly encourage code deposition in a community repository (e.g. GitHub). See the Nature Portfolio guidelines for submitting code & software for further information.

Data

Policy information about availability of data

All manuscripts must include a data availability statement. This statement should provide the following information, where applicable:

- Accession codes, unique identifiers, or web links for publicly available datasets
- A description of any restrictions on data availability
- For clinical datasets or third party data, please ensure that the statement adheres to our policy

All data generated or analysed during this study are included in the manuscript and supporting files. Source data are provided with this paper. The genetic screen data generated in this study are provided in the Source Data file. The mass-spectrometry data generated in this study have been deposited in the PRIDE database under accession codes PXD051267.

Research involving human participants, their data, or biological material

Policy information about studies with human participants or human data. See also policy information about sex, gender (identity/presentation), and sexual orientation and race, ethnicity and racism.

Reporting on sex and gender	does not apply
Reporting on race, ethnicity, or other socially relevant groupings	does not apply
Population characteristics	does not apply
Recruitment	does not apply
Ethics oversight	does not apply

Note that full information on the approval of the study protocol must also be provided in the manuscript.

Field-specific reporting

Please select the one below that is the best fit for your research. If you are not sure, read the appropriate sections before making your selection.

☒ Life sciences ☐ Behavioural & social sciences ☐ Ecological, evolutionary & environmental sciences

For a reference copy of the document with all sections, see nature.com/documents/nr-reporting-summary-flat.pdf

Life sciences study design

All studies must disclose on these points even when the disclosure is negative.

Sample size	Sample sizes were not predetermined using formal statistical power calculations. Instead, sample sizes were chosen based on established standards in the field for reverse genetics, genetic screens, proteomics, and in vitro ubiquitination assays, as well as on prior published studies using comparable experimental designs. Experiments were performed with sufficient biological and technical replicates to ensure reproducibility and robustness of the observed effects. Sample sizes were sufficient to yield consistent and statistically interpretable results across independent experiments.<br><br>Samples were allocated into experimental groups based on predefined experimental variables such as genotype, genetic perturbation, or biochemical condition. Allocation was therefore deterministic rather than random and reflected the specific hypotheses being tested. Because comparisons were made between well-defined molecular conditions processed in parallel using identical protocols, potential covariates were inherently controlled. Randomization was not relevant to this study, as no heterogeneous subject populations or confounding variables were involved.
Data exclusions	No data were excluded from analysis
Replication	All Western blots presented within individual figure panels are from the same experiment and gels/blots were processed in parallel. The experiments represented in Fig. 1c, 3g, 3h, 4g, 4h, 5a, 5b, 5c, 5d, 5e, 5f, 5g, 5h, 5i, 5j were repeated twice independently with similar results. The experiments represented in Fig. 4c, 4d, were repeated independently three times with similar results. The experiments represented in Fig. 4i and 5k were performed once. The experiments represented in Supplementary Fig. 1e, 1g, 4c, 5b, 5e, 5f, 5g, 6b, 8a, 8c, 8e, 8g were repeated twice independently with similar results. The experiments represented in Supplementary Fig. 4d, 7c, 9b were repeated independently three times with similar results. The experiments represented in Supplementary Fig. 1b, 7d, 9a were performed once.
Randomization	Does not apply;
Blinding	Blinding was not applied in this study because it was not relevant to the experimental design. The experiments consisted of molecular and

Blinding	biochemical assays including reverse genetics, genetic screens, proteomics, and in vitro ubiquitination assays. Group allocation was defined by genotype, construct, or experimental condition and outcomes were measured using objective and quantitative readouts such as protein abundance, modification status, or interaction profiles. Data collection and analysis did not involve subjective assessment, and all samples were processed using standardized protocols. Therefore, blinding would not have influenced data acquisition or interpretation.

Reporting for specific materials, systems and methods

We require information from authors about some types of materials, experimental systems and methods used in many studies. Here, indicate whether each material, system or method listed is relevant to your study. If you are not sure if a list item applies to your research, read the appropriate section before selecting a response.

Materials & experimental systems

n/a	Involved in the study
☐	☒ Antibodies
☐	☒ Eukaryotic cell lines
☒	☐ Palaeontology and archaeology
☒	☐ Animals and other organisms
☒	☐ Clinical data
☒	☐ Dual use research of concern
☒	☐ Plants

Methods

n/a	Involved in the study
☒	☐ ChIP-seq
☐	☒ Flow cytometry
☒	☐ MRI-based neuroimaging

Antibodies

Antibodies used

All antibodies were purchased commercially, and are all described in the Suppl. Methods

ARP10 Antibody 1:1000 for WB
1:100 for IF Novus
1:1000 Cat# NBP1-91682; RRID:AB_11036260
nti-APOBEC3B Antibody (EPR18138) 1:1000 Abcam
1:1000 Cat# ab184990; RRID:AB_2891094
Anti-APOBEC3G (D9C6Z) Rabbit mAb 1:1000 Cell Signaling Technology
1:1000 Cat# 43584
RRID:AB_2799245
Anti-MYC antibody (4A6) 1:5000 Millipore
1:5000 Cat# 05-724; RRID:AB_11211891
HA-Tag (C29F4) Rabbit mAb 1:1000 for WB
1:100 for Co-IP Cell Signaling Technology Cat# 3724; RRID:AB_1549585
HA-Tag (6E2) Mouse mAb 1:1000 for WB
1:100 for Co-IP Cell Signaling Technology Cat# 2367; RRID:AB_10691311
OLLAS Epitope Tag Antibody (L2) 1:4000 Novus Cat# NBP1-06713; RRID:AB_1625979
LC3B Antibody 1:1000 Cell Signaling Technology Cat# 2775; RRID:AB_915950
Ubiquitin Antikörper (P4D1) 1:1000 Santa Cruz Biotechnology Cat# sc-8017; RRID:AB_628423
Anti-UBR4/p600 antibody 1:1000 Abcam Cat# ab86738; RRID:AB_1952666
Rabbit anti-EDD1 Antibody 1:1000 Bethyl Cat# A300-573A; RRID:AB_2210189
Rabbit anti-Lasu1/Ureb1 Antibody (HUWE1) 1:1000 Bethyl Cat# A300-486A; RRID:AB_2264590
XBP-1s (E9V3E) Rabbit mAb 1:1000 Cell Signaling Technology Cat# 40435;
RRID: AB_2891025
Monoclonal Anti-α-Tubulin antibody produced in mouse 1:1000 Sigma-Aldrich Cat# T9026; RRID:AB_477593
Lamin A/C Antibody (E-1) 1:1000 Santa Cruz Biotechnology Cat# sc-376248; RRID:AB_10991536
Monoclonal Anti-Vinculin antibody 1:1000 Sigma-Aldrich Cat# V9131; RRID:AB_477629
Penta·His Antibody, BSA-free Qiagen Cat# 34660
RRID:AB_2619735
Anti-beta Actin antibody (HRP) (AC-15) 1:20000 Abcam Cat# ab49900; RRID:AB_867494
Anti-rabbit IgG, HRP-linked Antibody 1:3500 Cell Signaling Technology Cat# 7074; RRID:AB_2099233
Anti-mouse IgG, HRP-linked Antibody 1:3500 Cell Signaling Technology Cat# 7076; RRID:AB_330924
Goat-anti-mouse IgG Light Chain HRP 1:5000 Jackson ImmunoResearch Labs Cat# 115-035-174; RRID:AB_2338512
Goat Anti-Rat IgG H&L (HRP) 1:50000 Abcam Cat# ab97057
RRID:AB_10680316
APC anti-rat CD90/mouse CD90.1 (Thy-1.1) Antibody 1:260 BioLegend Cat# 202526; RRID:AB_1595470
Donkey F(ab')2 Anti-Rabbit IgG - H&L (Alexa Fluor 488), pre-adsorbed 1:800 for IF Abcam Cat# ab181346; RRID:AB_2813899

Validation

Antibodies detecting UBR4, UBR5, HUWE1 were validated by knock-out (Suppl. Fig. 2c, 3a, 9b). APOBEC3B were validated in cell lines in which the gene expressing A3B was knocked out.; these data are not included in the manuscript. None of the other used antibodies were validated by knock-out.

Eukaryotic cell lines

Policy information about cell lines and Sex and Gender in Research

Cell line source(s)	All cell lines are described in the Suppl. Methods:<br><br>HEK293T ATCC CRL-3216<br>Lenti-X™ 293T Cell Line Takara Cat# 632180<br>HeLa ATCC CCL-2<br>RKO ATCC CRL-2577<br>RKO-DOX-Cas9-P2A-BFP Michlits G., et al., 2020 N/A<br>RKO-DOX-Cas9-P2A-GFP de Almeida M., Hinterndorfer M. et al., 2021 N/A<br>RKO-MYC-mCherry-P2A-OLLAS-A3H-I This study N/A<br>RKO-MYC-mCherry-P2A-OLLAS-A3H-II This study N/A<br>RKO-DOX-Cas9-mCherry-A3H-II-P2A-EGFP-A3H-I This study N/A<br>RKO-DOX-Cas9-MYC-mCherry-P2A-3xHA-A3H-I This study N/A<br>THP-1 ATCC TIB-202<br>THP-1-DOX-Cas9-P2A-GFP This study N/A<br>RKO-DOX-MYC-TurboID-A3H-I-mCherry-P2A-rtTA This study N/A<br>RKO-DOX-MYC-TurboID-A3H-II-mCherry-P2A-rtTA This study N/A<br>RKO-DOX-MYC-TurboID-GFP-mCherry-P2A-rtTA This study N/A<br>RKO-DOX-Cas9-MYC-mCherry-P2A-OLLAS-EGFP-A3H-I This study N/A<br>RKO-DOX-Cas9-MYC-mCherry-P2A-OLLAS-EGFP-A3H-II This study N/A<br>RKO-DOX-Cas9-MYC-mCherry-P2A-OLLAS-EGFP-A3H-II-W155A This study N/A<br>RKO-DOX-Cas9-MYC-mCherry-P2A-OLLAS-EGFP-A3H-II-R175/176E This study N/A<br>RKO-DOX-Cas9-MYC-mCherry-P2A-OLLAS-EGFP-A3H-I-G105R This study N/A<br>RKO-DOX-Cas9-MYC-mCherry-P2A-OLLAS-EGFP-A3H-II-R105G This study N/A<br>RKO-DOX-Cas9-MYC-mCherry-P2A-OLLAS-EGFP-A3H-II-E56A-W155A-R175/176E This study N/A<br>RKO-DOX-Cas9-MYC-mCherry-P2A-3xHA-A3H-I UNG2-/- This study N/A
Authentication	Cell lines used in this study were authenticated by STR
Mycoplasma contamination	Cell lines were routinely tested for mycoplasma, and found to be mycoplasma-negative.
Commonly misidentified lines<br>(See ICLAC register)	None of the used cell lines are commonly misidentified lines.

Plants

Seed stocks	does not apply
Novel plant genotypes	does not apply
Authentication	does not apply

Flow Cytometry

Plots

Confirm that:

☒ The axis labels state the marker and fluorochrome used (e.g. CD4-FITC).

☒ The axis scales are clearly visible. Include numbers along axes only for bottom left plot of group (a 'group' is an analysis of identical markers).

☐ All plots are contour plots with outliers or pseudocolor plots.

☐ A numerical value for number of cells or percentage (with statistics) is provided.

Methodology

Sample preparation	Sample preparation is described in the manuscript and its associated files.

Instrument	Samples were collected using FACS Aria III cell sorter operated by BD FACSDiva software (v8.0), Bio-Rad ZE5 Cell Analyzer operated by Everest software, BD LSRFortessa Cell Analyzer operated by FACSDiva software.
Software	Samples were analyzed and plotted using FlowJo v10.8.1 software.
Cell population abundance	Live-sorted samples were routinely checked for purity using negative as well as positive controls, and only analyzed when the appropriate cell population was present.
Gating strategy	Gating strategy for the genetic screen is described in the materials and methods section. Cells were gated for live, single as well as relevant fluorophore-positive populations using negative as well as positive control samples.

☒ Tick this box to confirm that a figure exemplifying the gating strategy is provided in the Supplementary Information.
