# Supplementary material for: Guardian ubiquitin E3 ligases target cancer-associated APOBEC3 deaminases for degradation to promote human genome integrity
Source: Nat Commun. 2026 Jan 19;17:1723. doi: 10.1038/s41467-026-68420-5 (PMC12913773; doi:10.1038/s41467-026-68420-5)

Extended Data Fig. 1a.

Boxes indicate regions shown in figure. Each image is the same membrane stained with the indicated antibody.

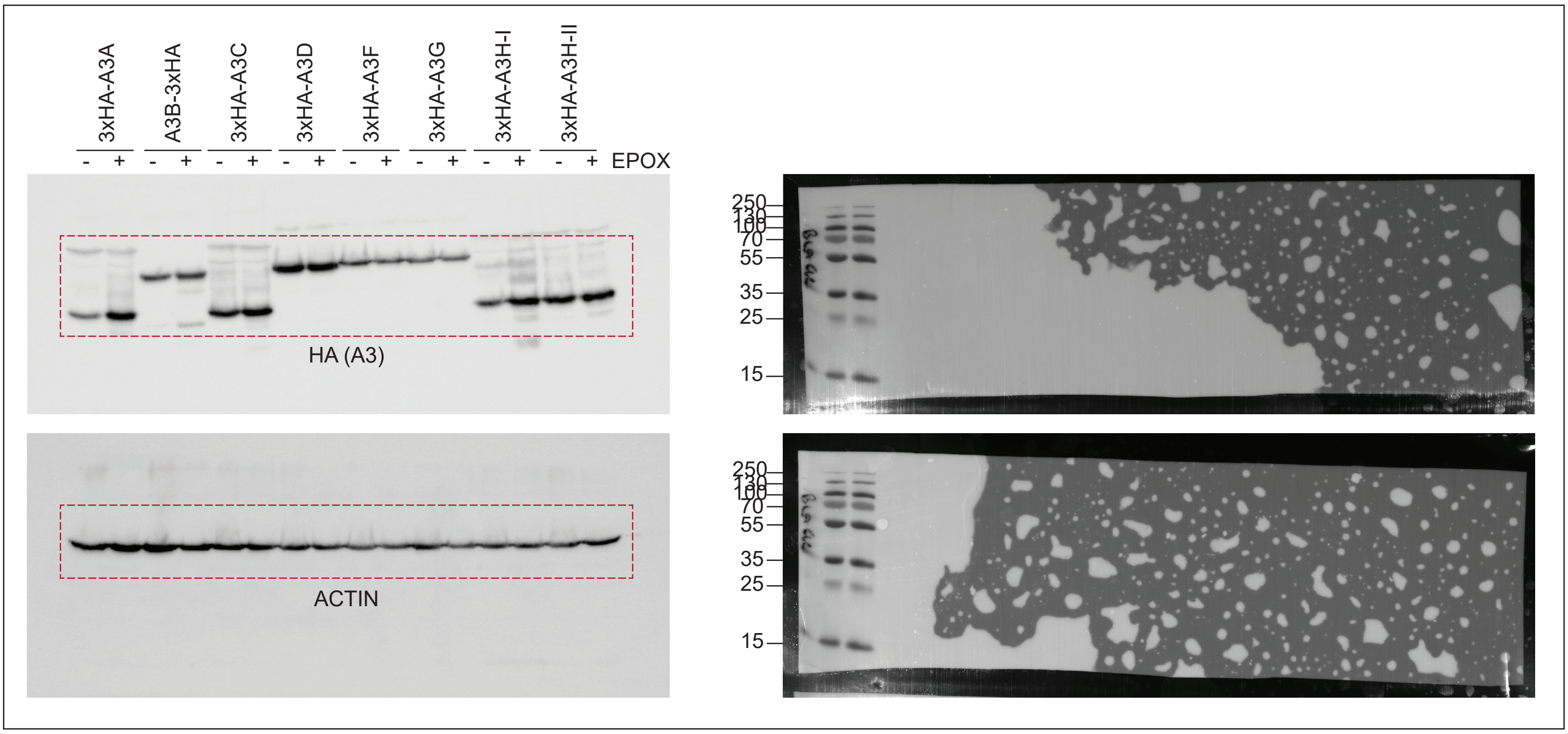

Extended Data Fig. 1d.
Boxes indicate regions shown in figure. Each image is the same membrane stained with the indicated antibody.

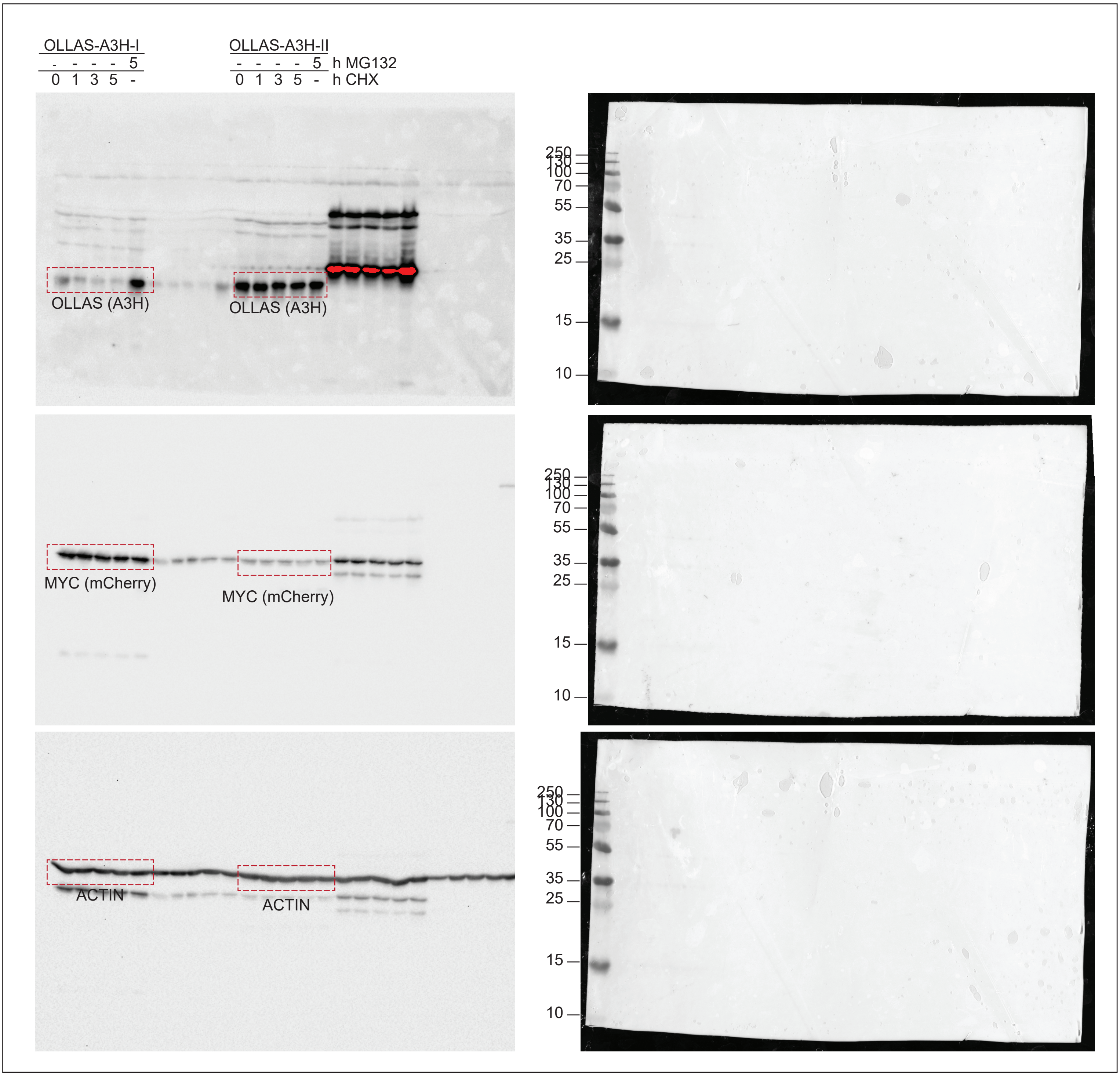

Extended Data Fig. 1g.
Boxes indicate regions shown in figure. Each image is the same membrane stained with the indicated antibody.

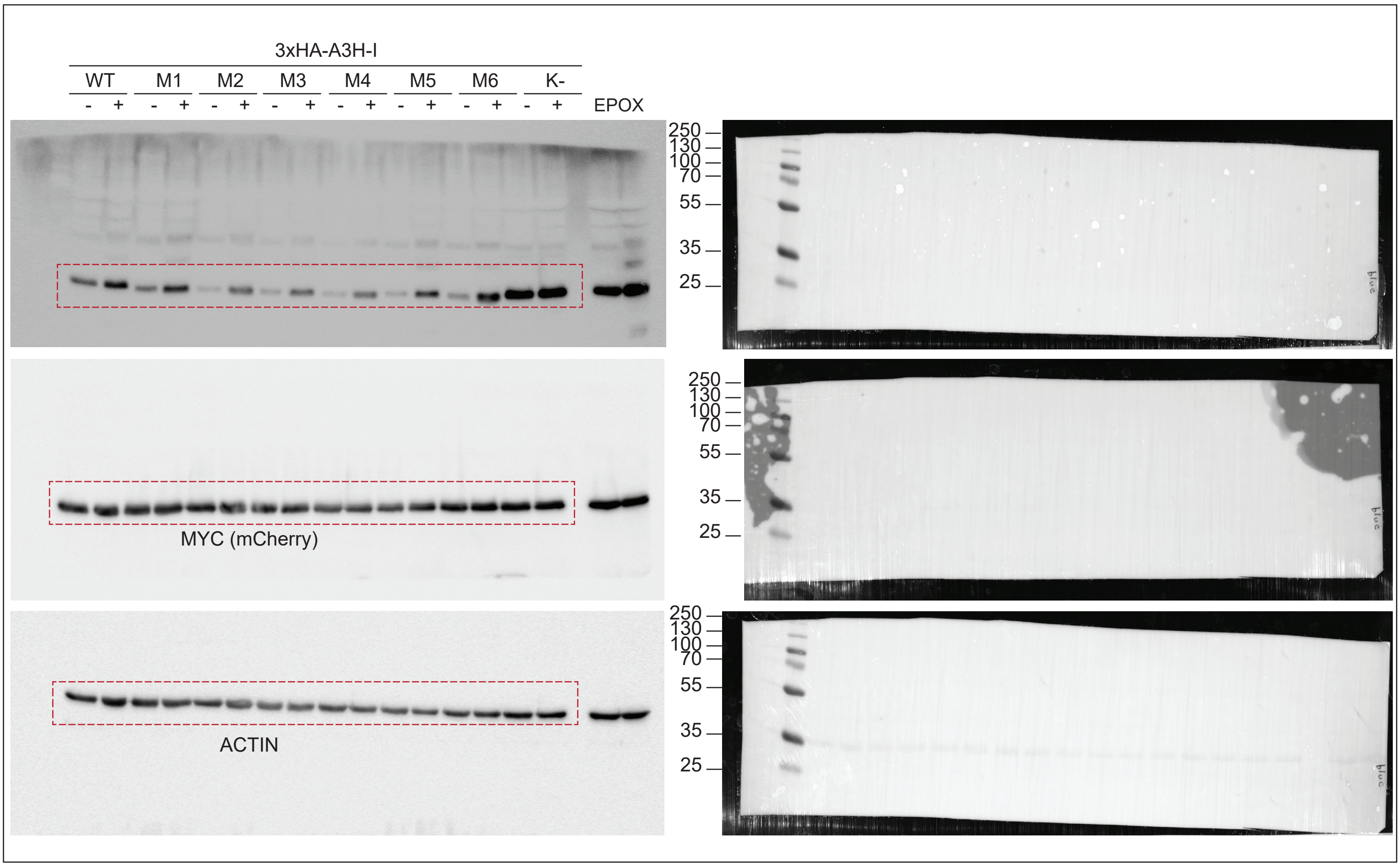

Supplement: Supplementary file 7 — Source data [file 41467_2026_68420_MOESM7_ESM.zip › Source data WB/Figure 1/Figure 1.pdf]
