# Supplementary material for: Guardian ubiquitin E3 ligases target cancer-associated APOBEC3 deaminases for degradation to promote human genome integrity
Source: Nat Commun. 2026 Jan 19;17:1723. doi: 10.1038/s41467-026-68420-5 (PMC12913773; doi:10.1038/s41467-026-68420-5)
Supplement: Supplementary file 7 — Source data [file 41467_2026_68420_MOESM7_ESM.zip › Source data WB/Figure 5/Figure 5.pdf]

Extended Data Fig. 5a
Box indicates region shown in figure. Gel was imaged with Trp fluorescence.

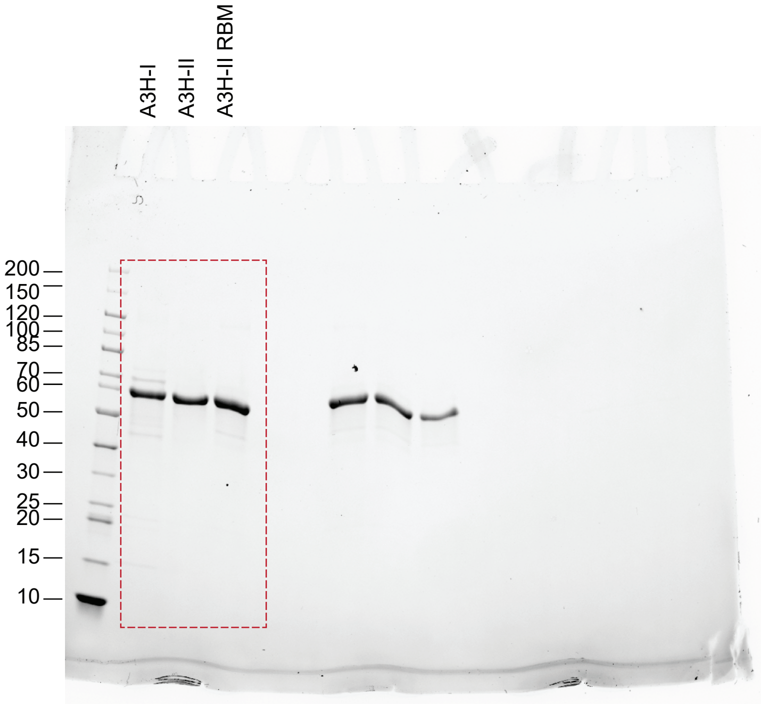

Extended Data Fig. 5b
Box indicates region shown in figure. SYBR Gold stained TBE-Urea gel.

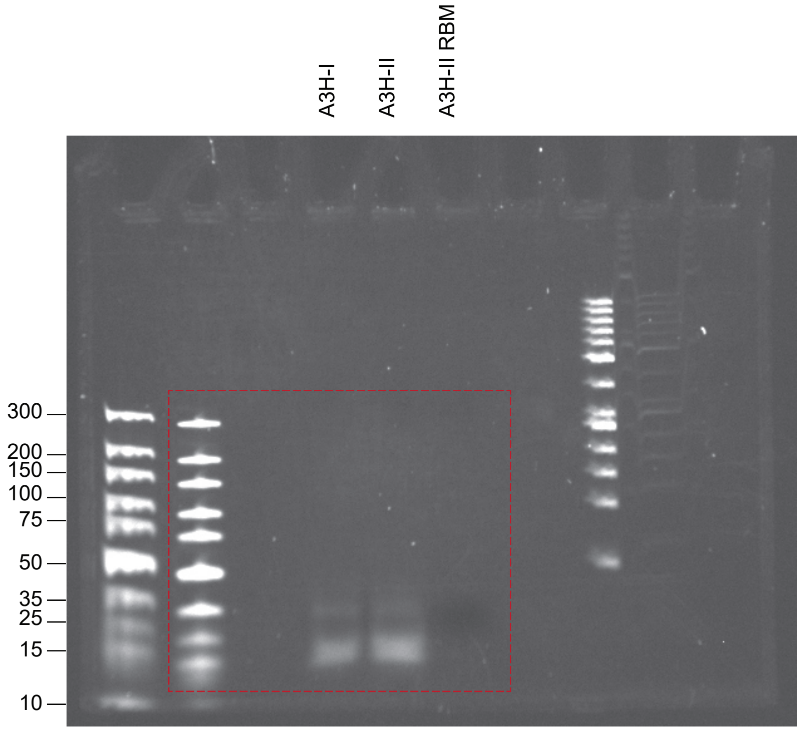

Extended Data Fig. 5c

Boxes indicate regions shown in figure. Same gel was imaged for in-gel fluorescence for dighlight-488 and Trp fluorescence.

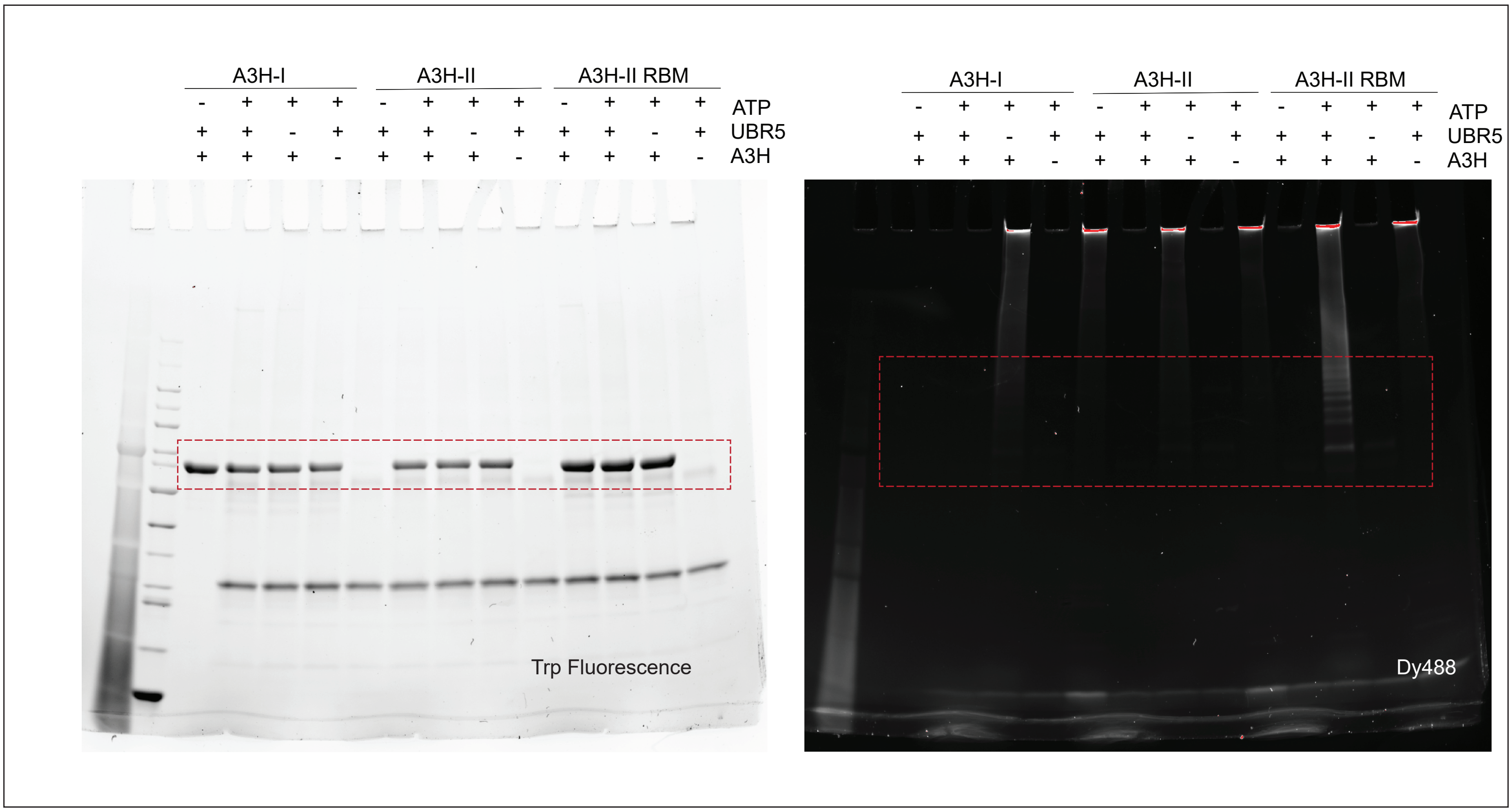

Extended Data Fig. 5d

Boxes indicate regions shown in figure. Same gel was imaged for in-gel fluorescence for dighlight-488 and Trp fluorescence.

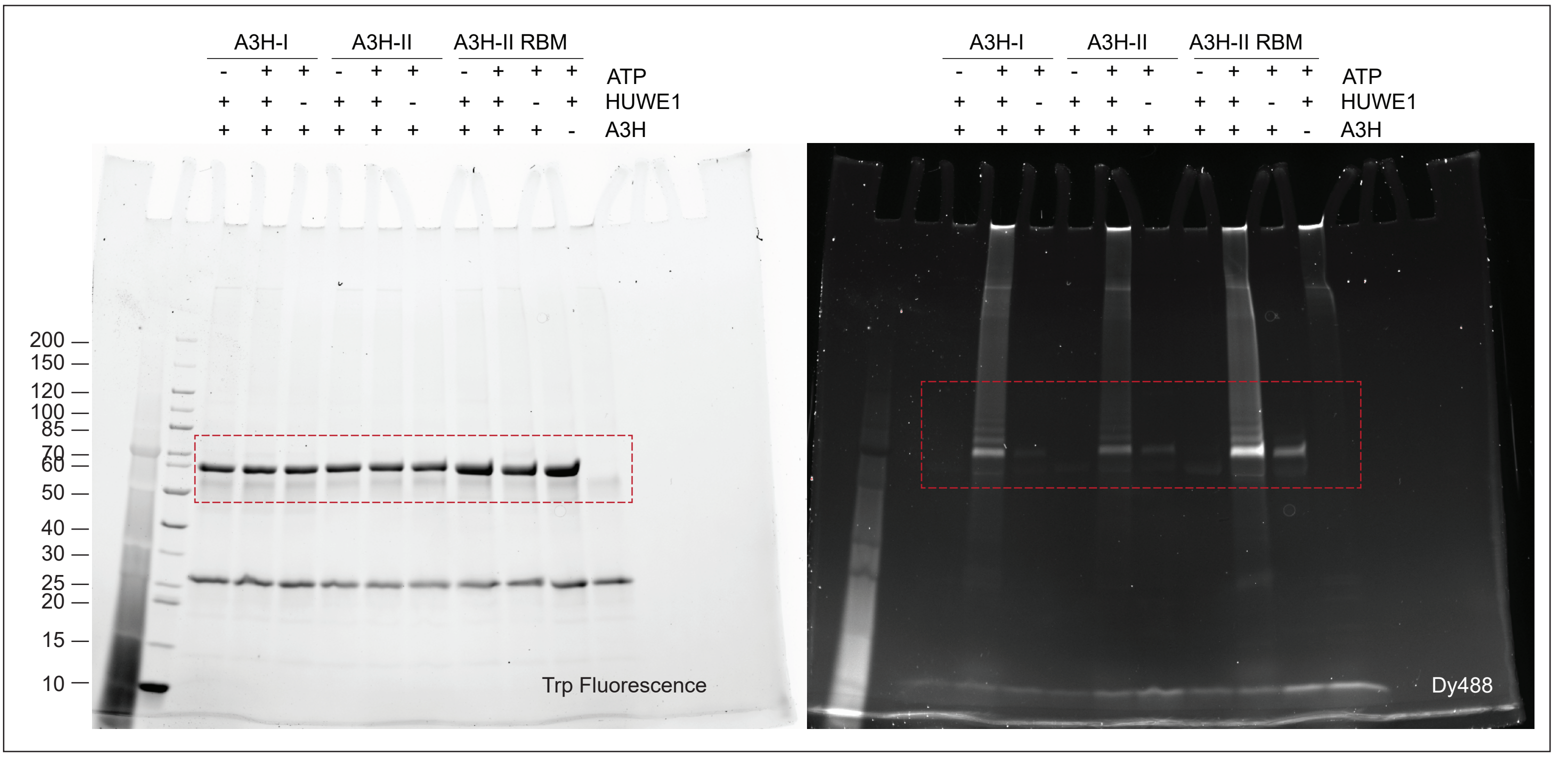

Extended Data Fig. 5e

Boxes indicate regions shown in figure. Same gel was imaged for in-gel fluorescence for dighlight-488 and Trp fluorescence.

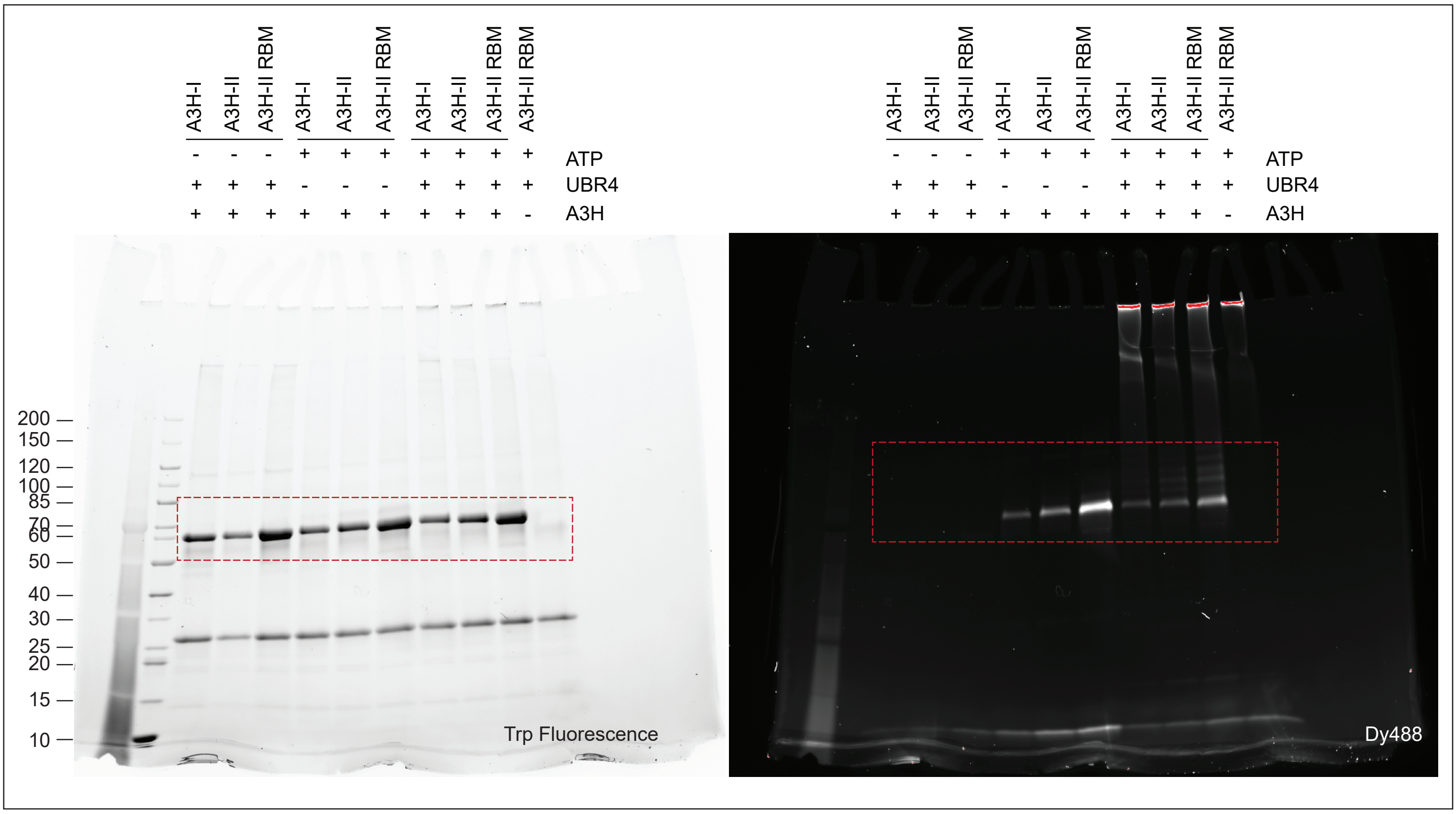

Extended Data Fig. 5f

Boxes indicate regions shown in figure. Same gel was imaged for in-gel fluorescence for dighlight-488 and Trp fluorescence.

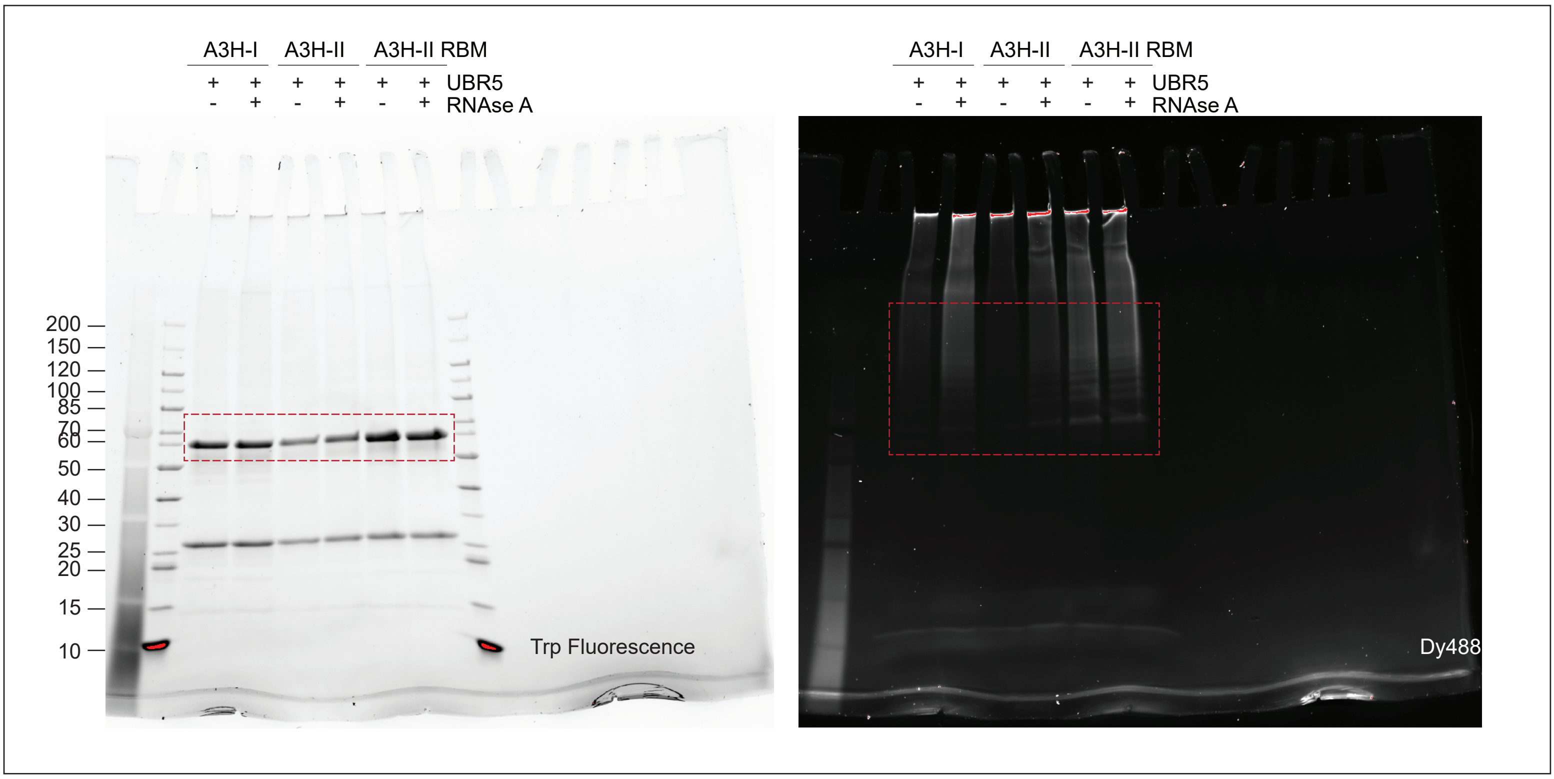

Extended Data Fig. 5g

Boxes indicate regions shown in figure. Same gel was imaged for in-gel fluorescence for dighlight-488 and Trp fluorescence.

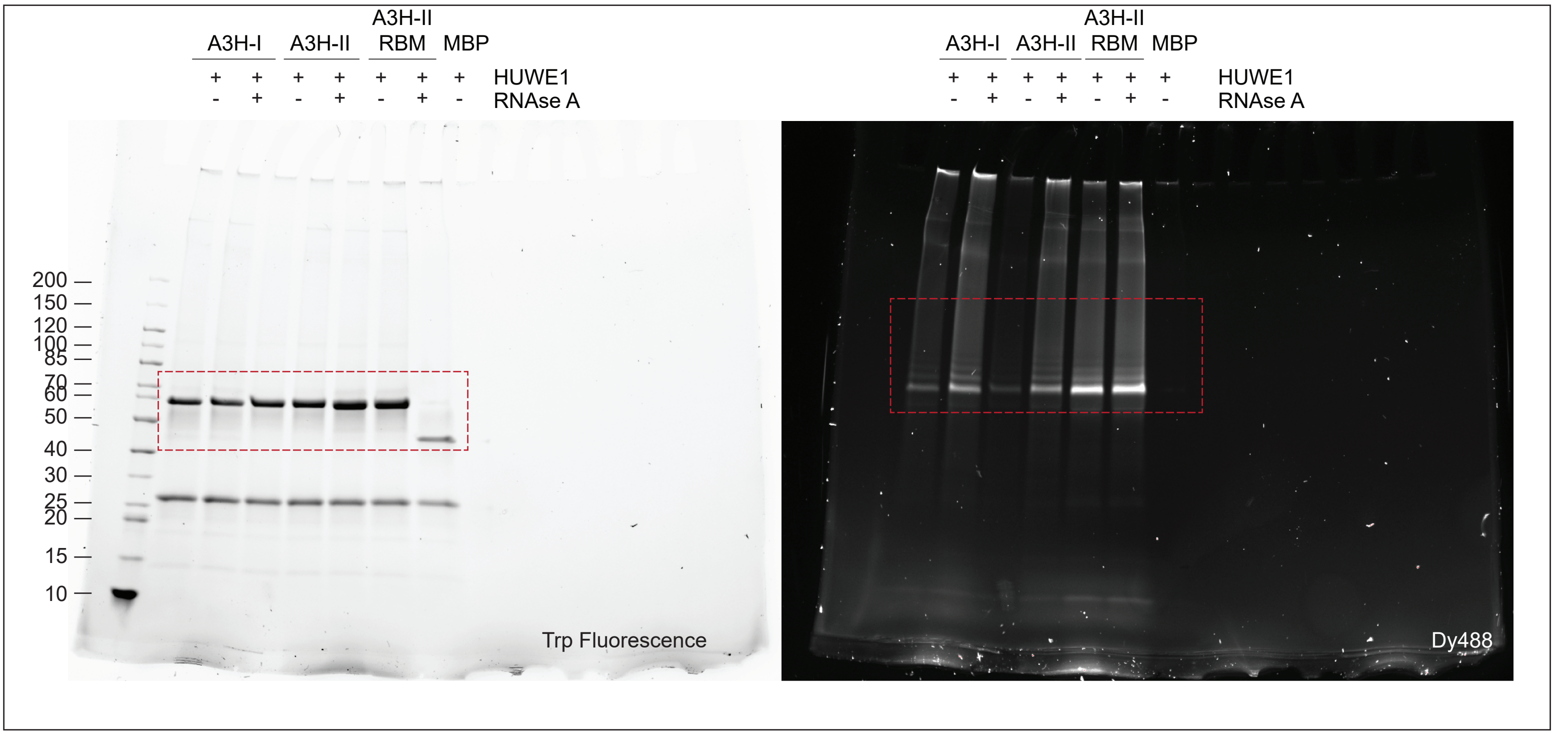

Extended Data Fig. 5h

Boxes indicate regions shown in figure. Same gel was imaged for in-gel fluorescence for dighlight-488 and Trp fluorescence.

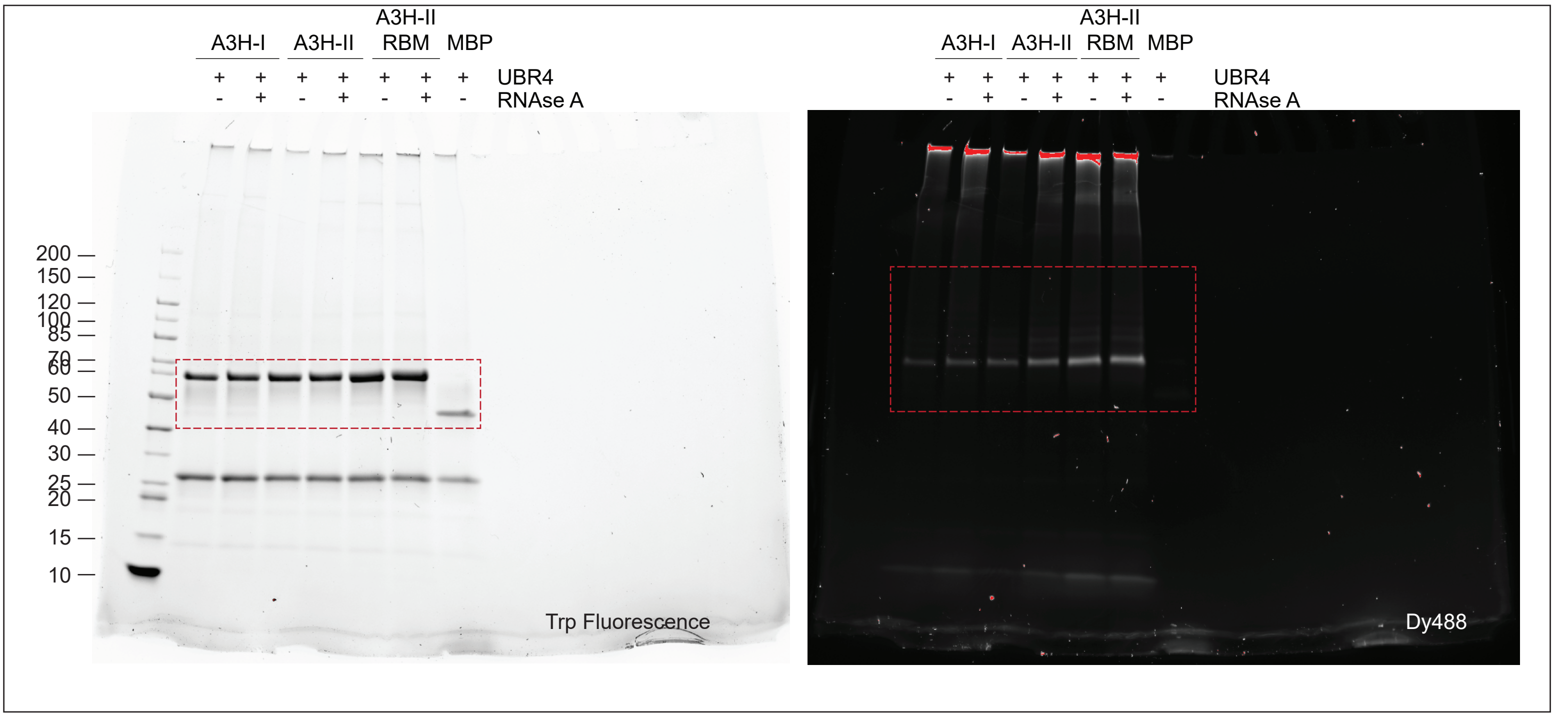

Extended Data Fig. 5i

Boxes indicate regions shown in figure. Gels were imaged for in-gel fluorescence and subsequently transfered onto a membrane and stained with the indicated antibody.

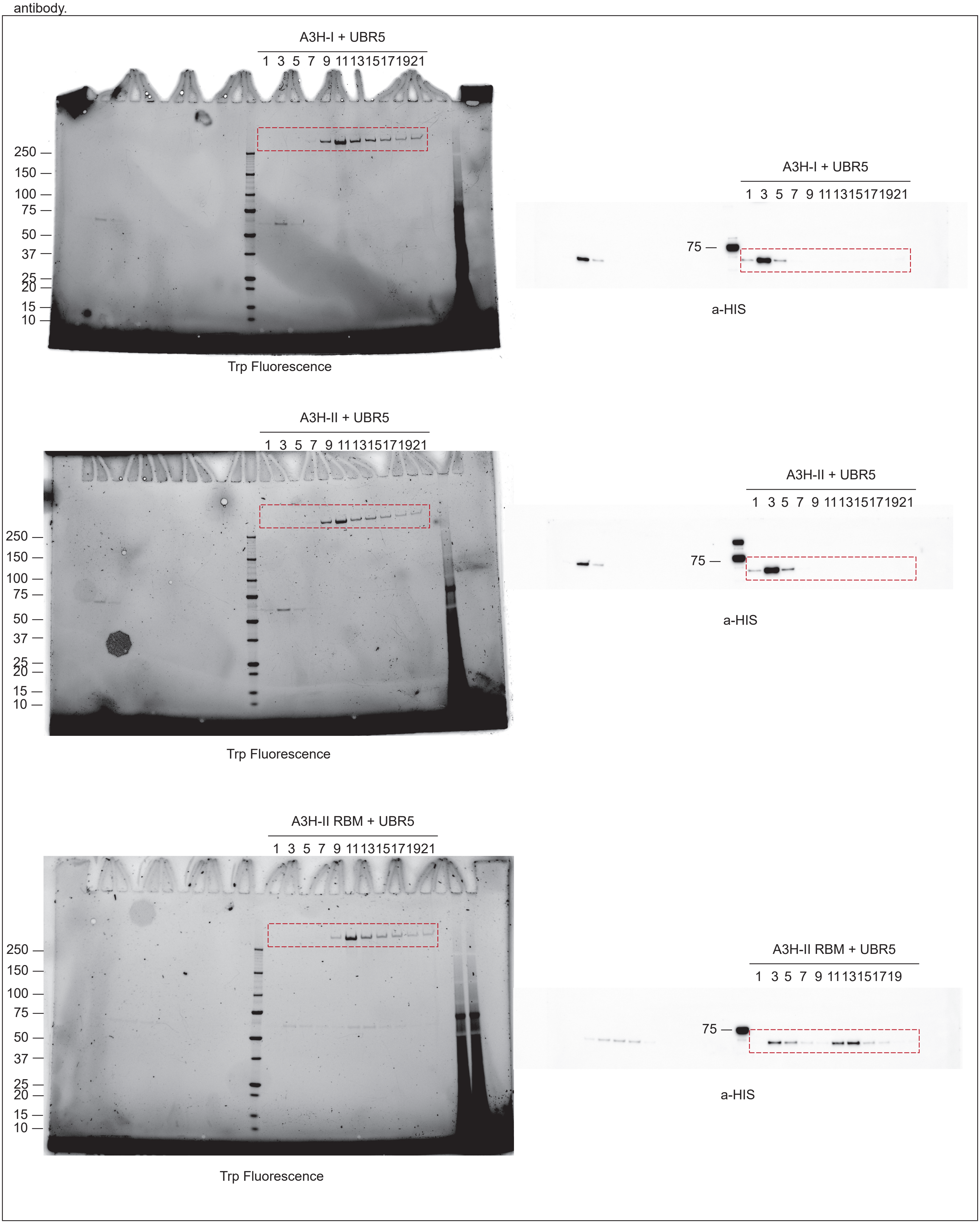

Extended Data Fig. 5j

Boxes indicate regions shown in figure. Gels were imaged for in-gel fluorescence and subsequently transfered onto a membrane and stained with the indicated antibody.

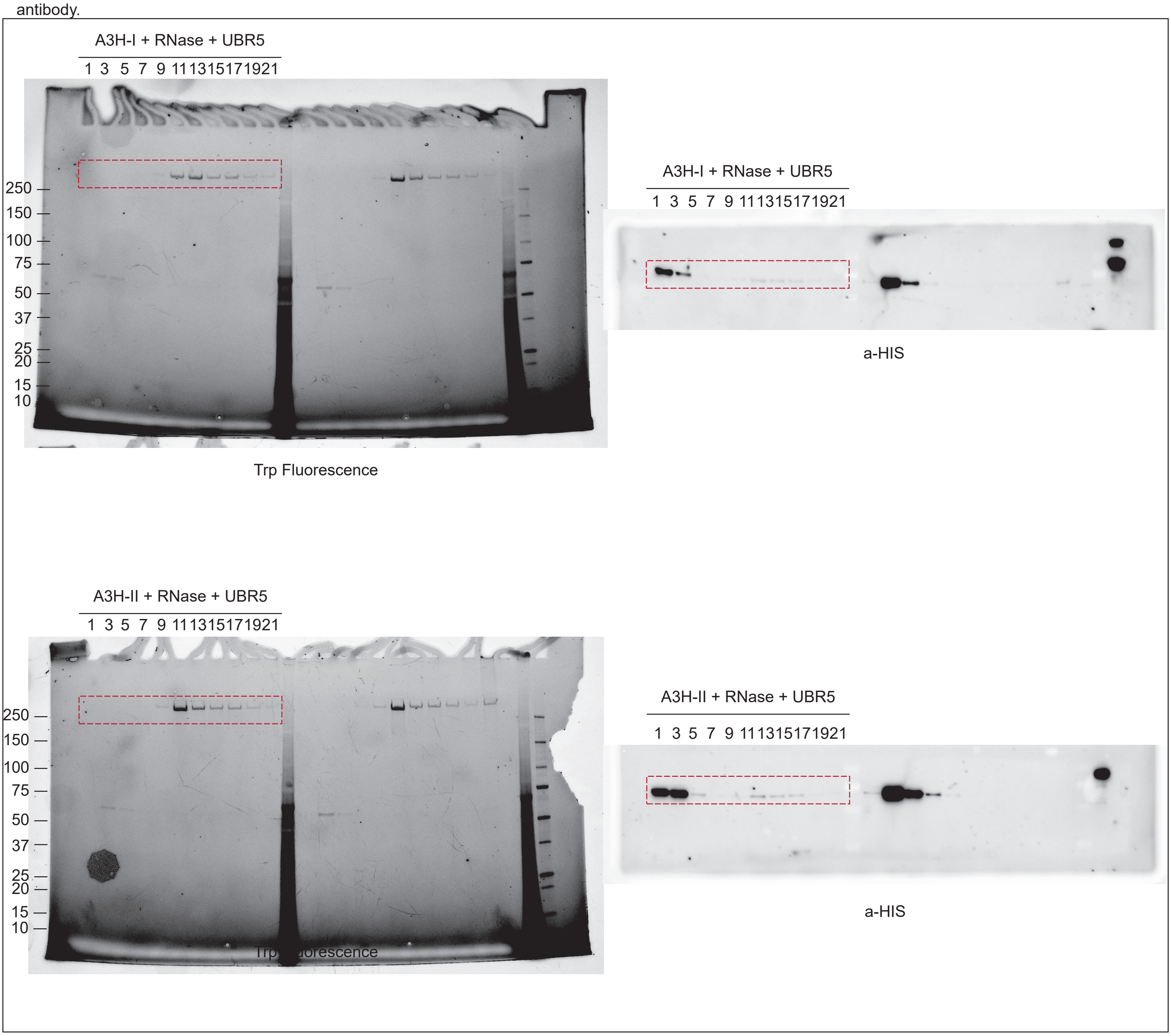

Extended Data Fig. 5k

Boxes indicate regions shown in figure. Gels were imaged for in-gel fluorescence.

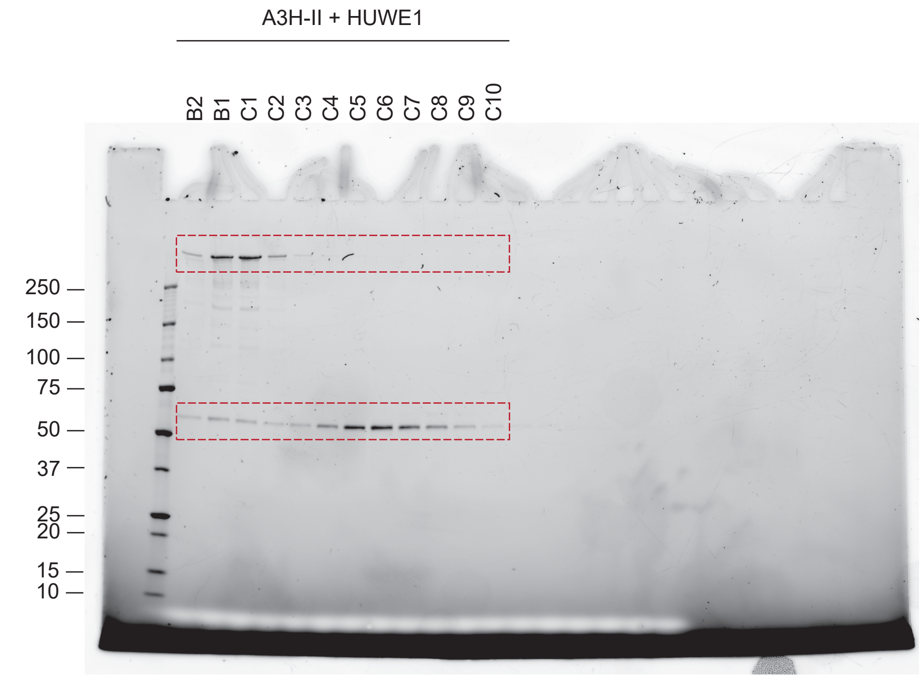

Trp Fluorescence

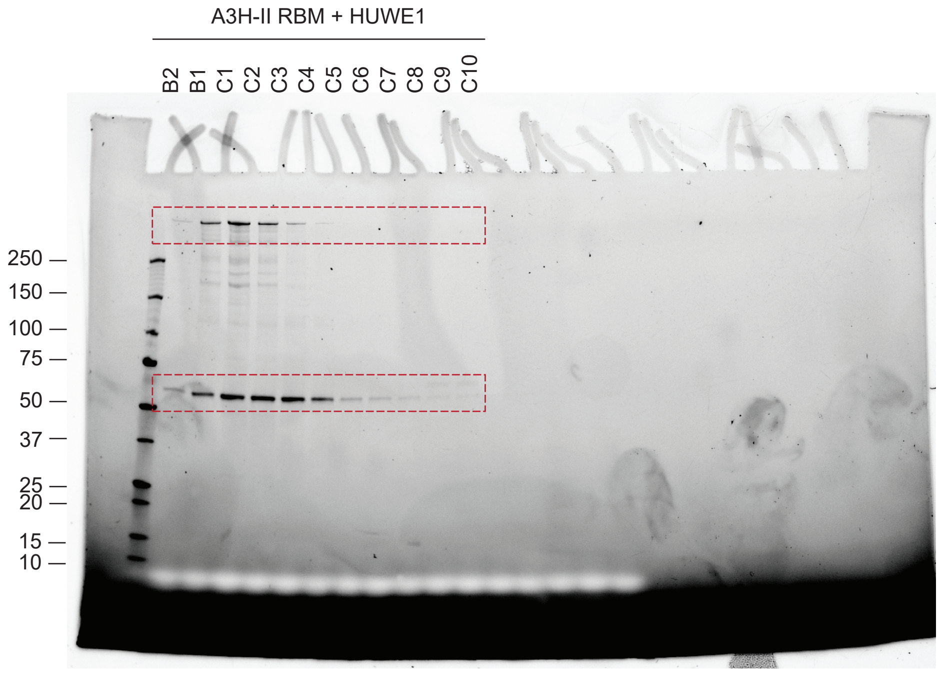

Trp Fluorescence
