# Supplementary material for: Guardian ubiquitin E3 ligases target cancer-associated APOBEC3 deaminases for degradation to promote human genome integrity
Source: Nat Commun. 2026 Jan 19;17:1723. doi: 10.1038/s41467-026-68420-5 (PMC12913773; doi:10.1038/s41467-026-68420-5)

Extended Data Supplementary Figure 4c

Boxes indicate regions shown in figure. Input and Streptavidin-enriched samples were loaded on three separate membranes: Membrane 1: Streptavidin-enriched probed for UBR5 and MYC (TID), Membrane 2: Streptavidin-enriched probed for HUWE1 and MYC (TID), Membrane 3 probed for UBR5, HUWE1, MYC (TID) and ACTIN.

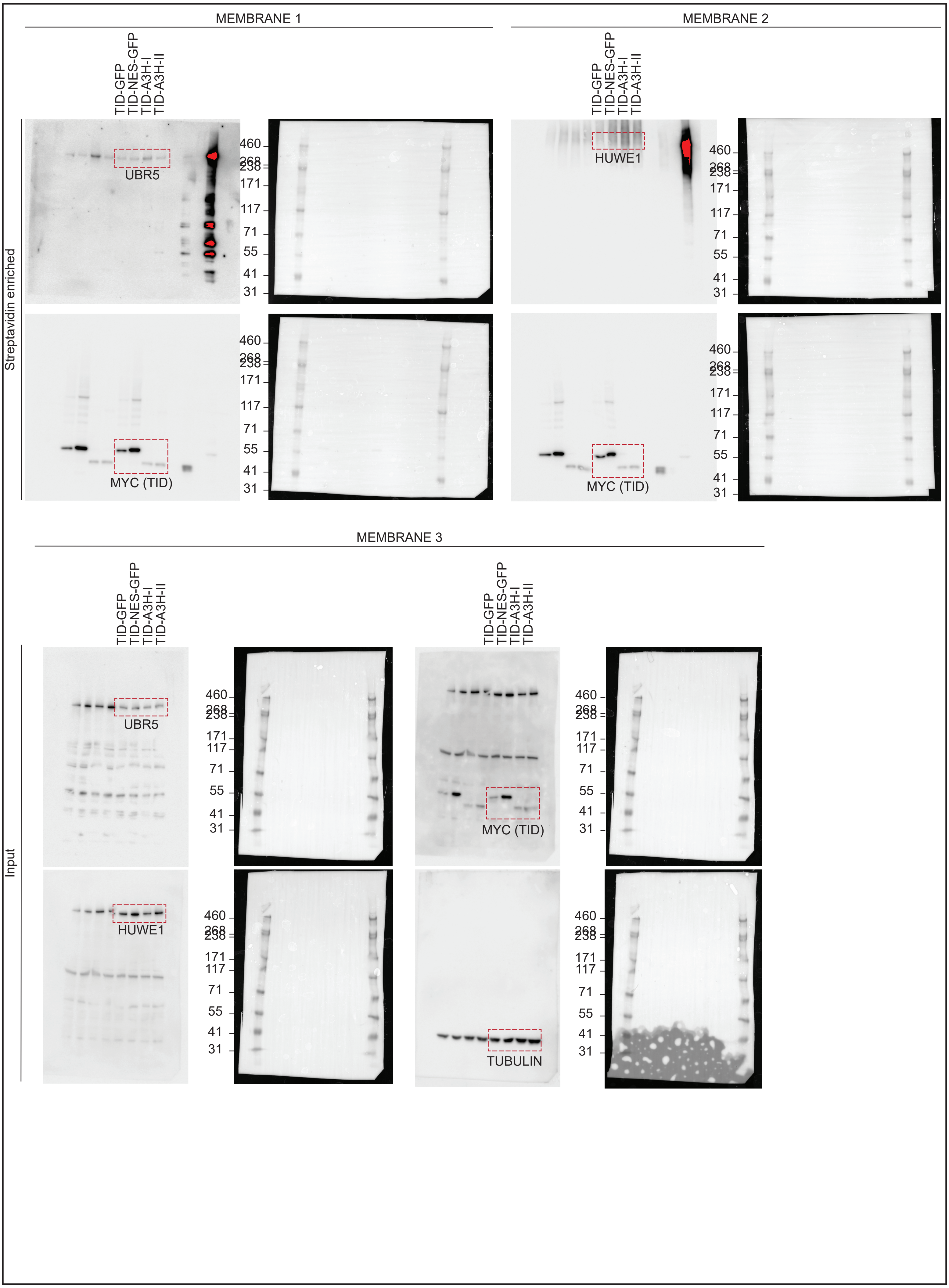

Extended Data Supplementary Figure 4d

Boxes indicate regions shown in figure. Input and immunoprecipitated samples (IP) were blotted on two separate membranes. Grouped images are the same membrane stained with the indicated antibody.

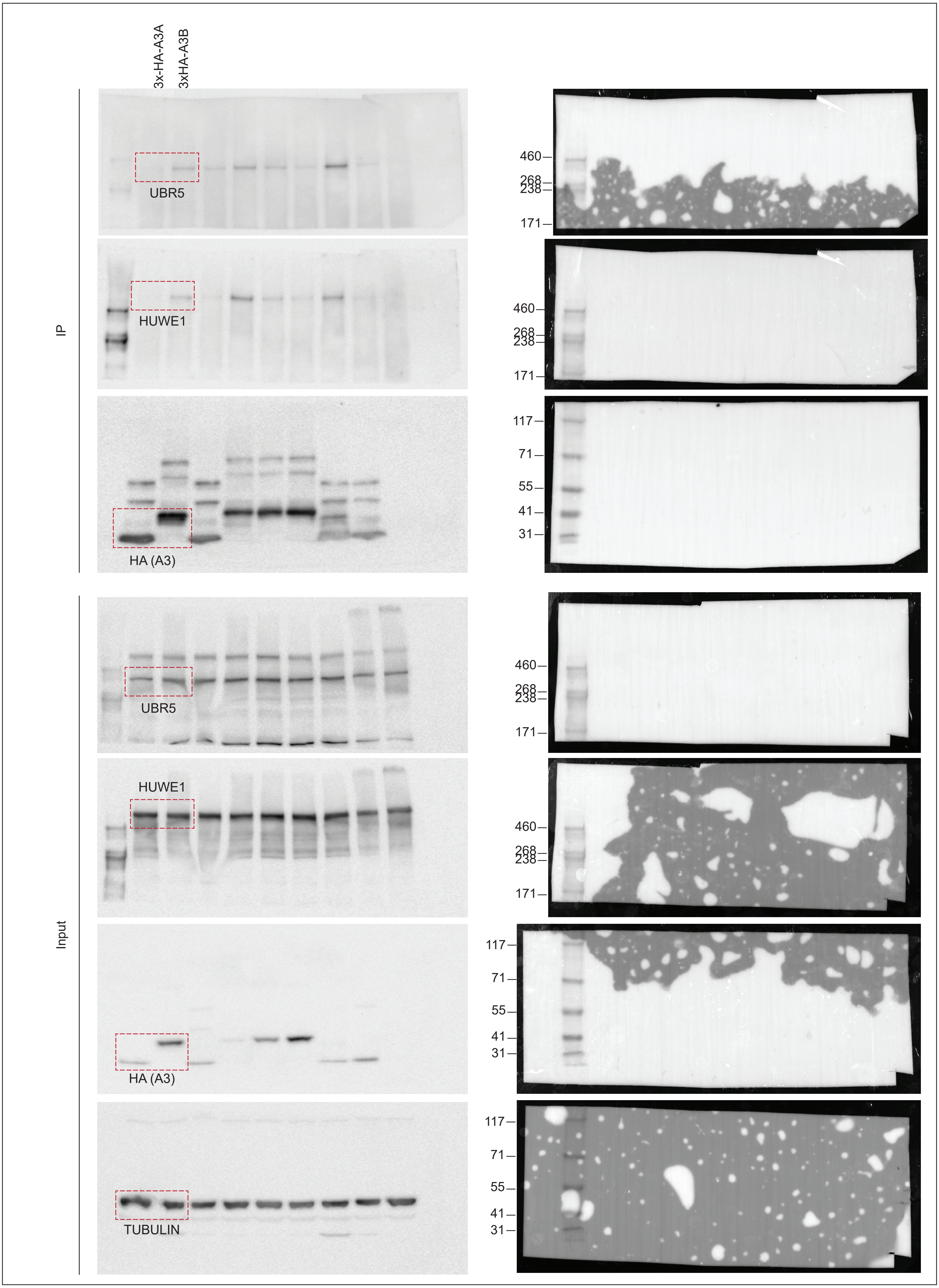

Supplement: Supplementary file 7 — Source data [file 41467_2026_68420_MOESM7_ESM.zip › Source data WB/Supplementary Figure 4/Supplementary Figure 4.pdf]
