# Supplementary material for: Guardian ubiquitin E3 ligases target cancer-associated APOBEC3 deaminases for degradation to promote human genome integrity
Source: Nat Commun. 2026 Jan 19;17:1723. doi: 10.1038/s41467-026-68420-5 (PMC12913773; doi:10.1038/s41467-026-68420-5)

Extended Data Supplementary Figure 6c

Boxes indicate regions shown in figure. Each image is the same membrane stained with the indicated antibody.

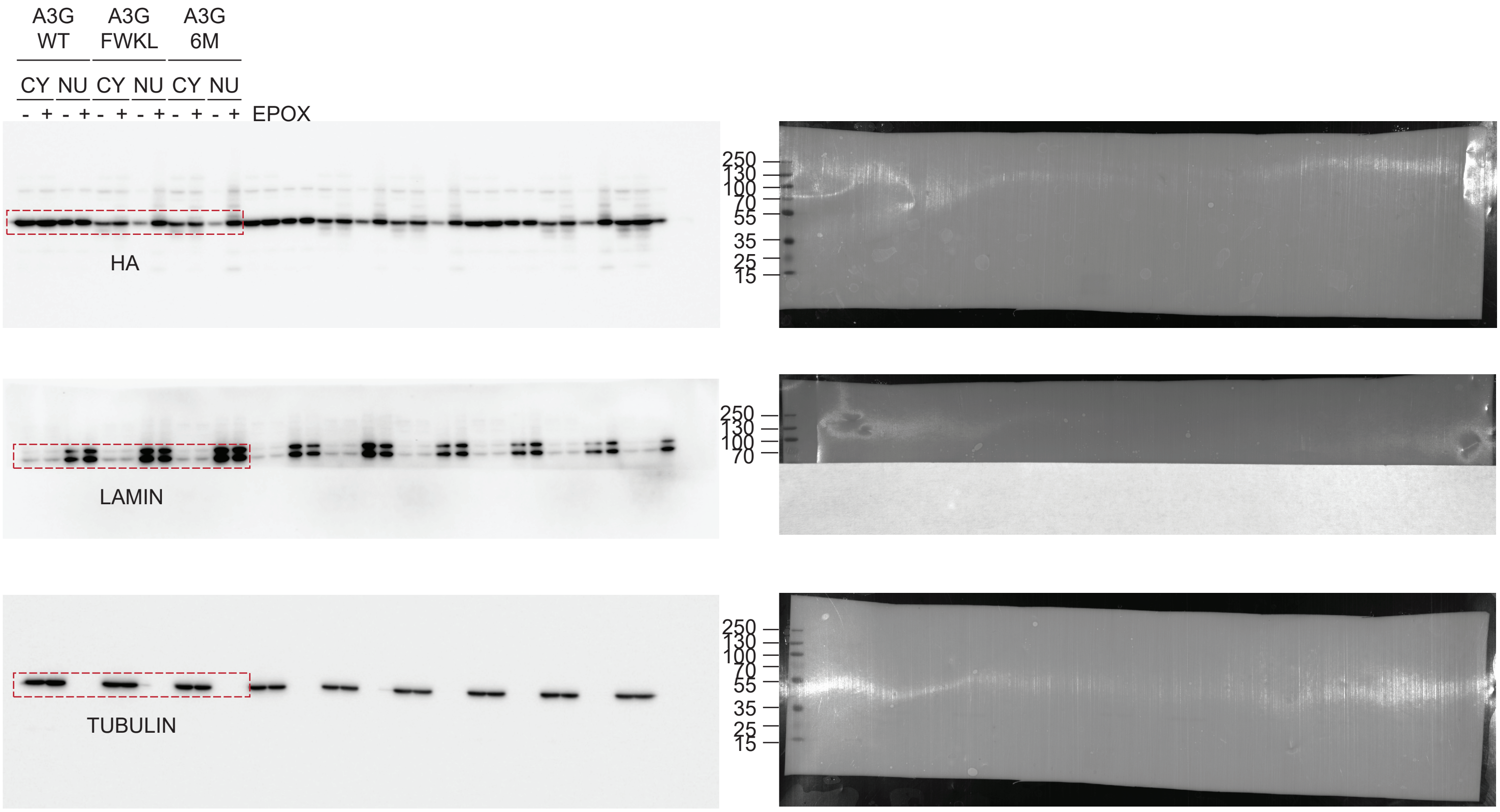

Extended Data Supplementary Figure 6e

Boxes indicate regions shown in figure. Each image is the same membrane stained with the indicated antibody.

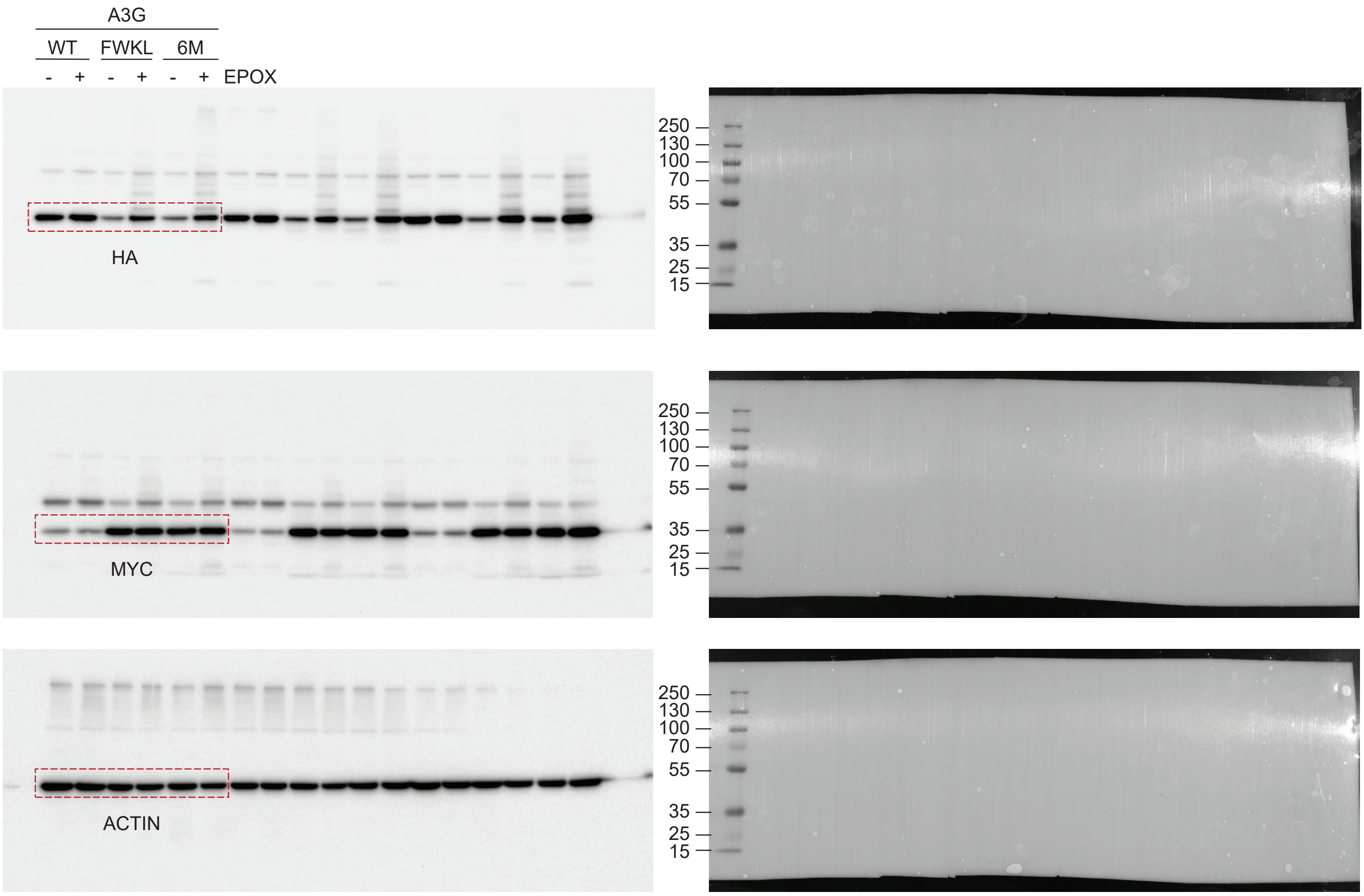

Extended Data Supplementary Figure 6g

Boxes indicate regions shown in figure. Each image is the same membrane stained with the indicated antibody.

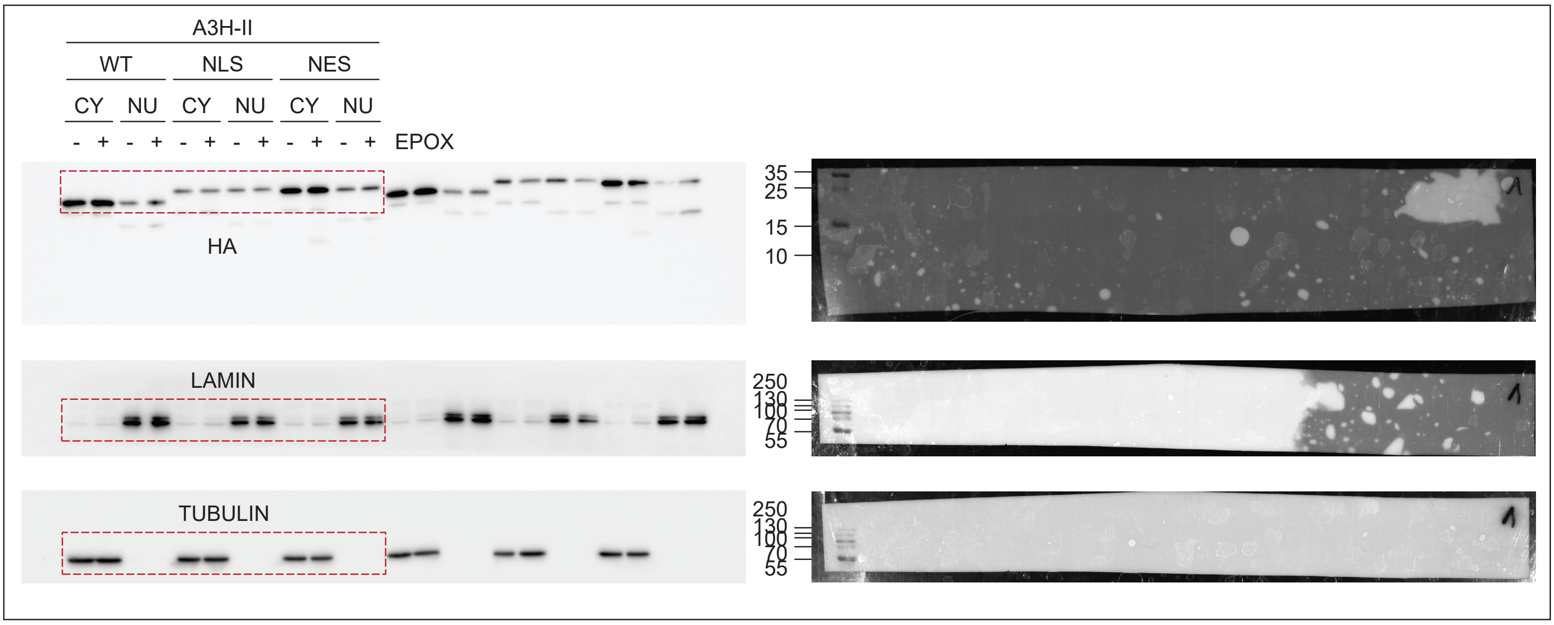

Extended Data Supplementary Figure 6h

Boxes indicate regions shown in figure. Each image is the same membrane stained with the indicated antibody.

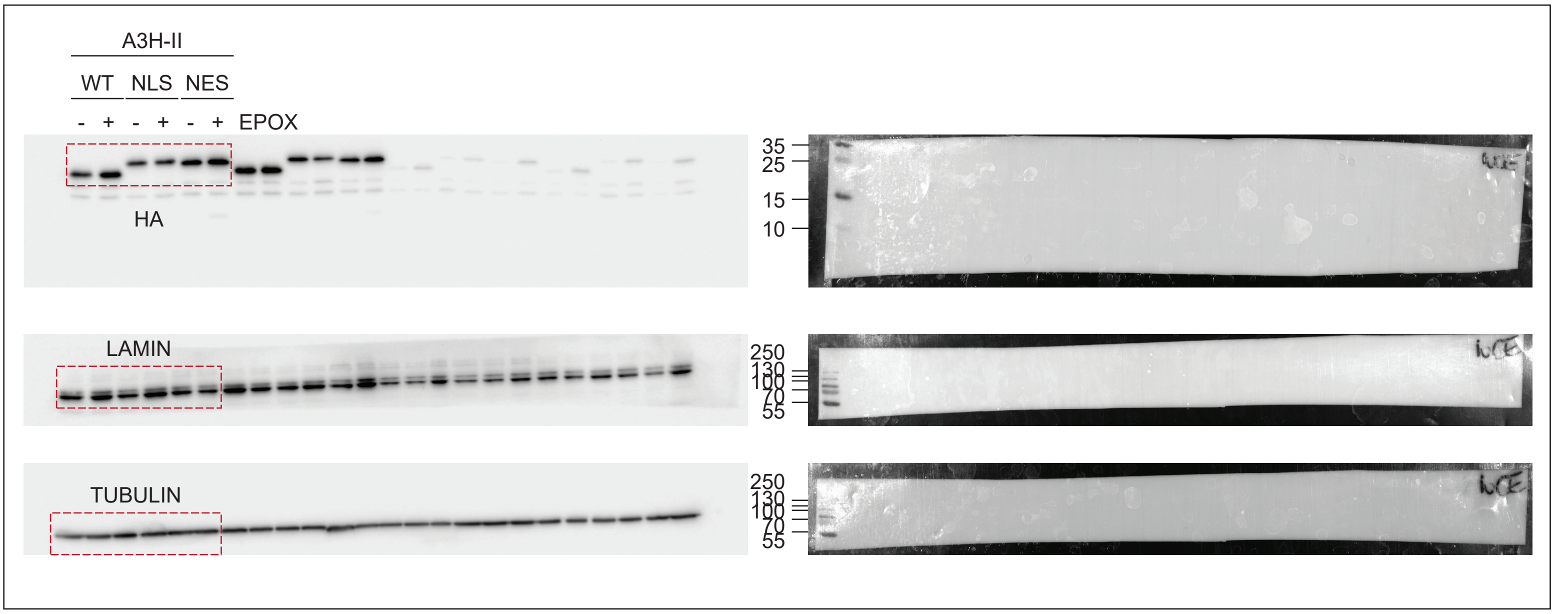

Extended Data Supplementary Figure 6j

Boxes indicate regions shown in figure. Each image is the same membrane stained with the indicated antibody.

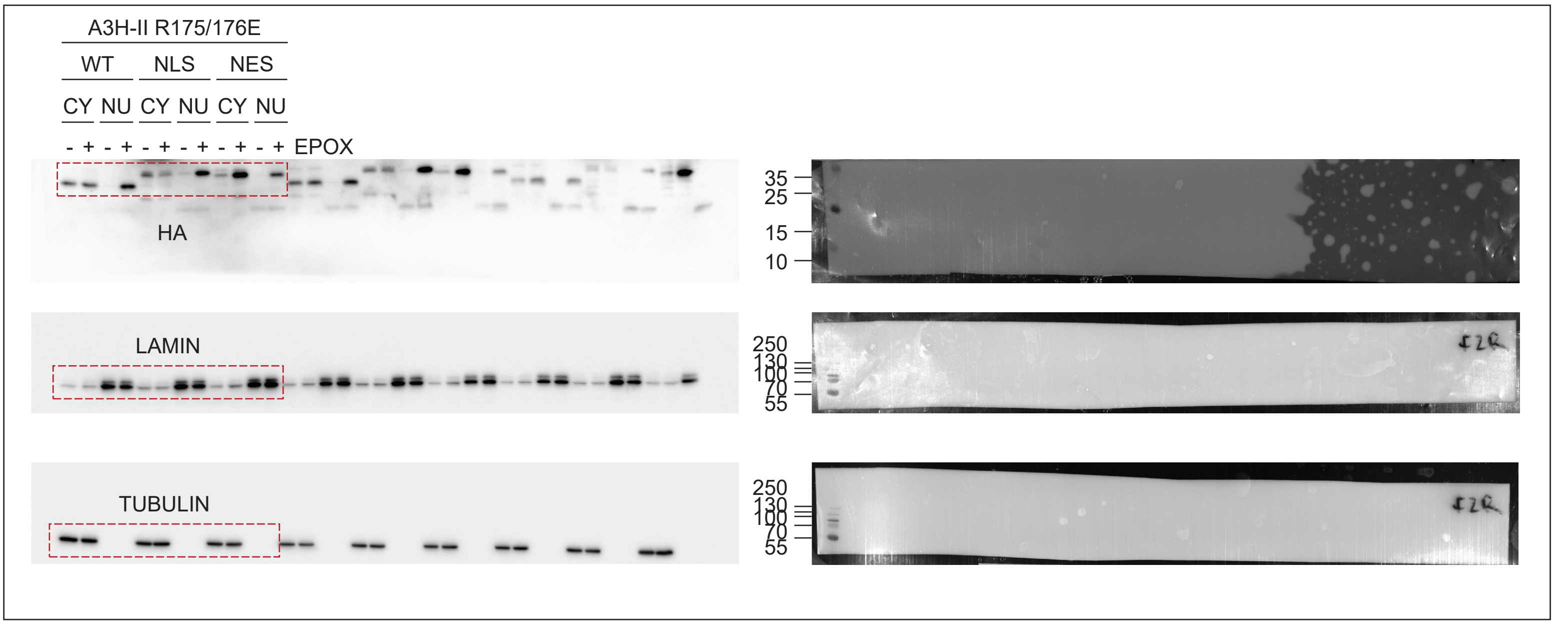

Extended Data Supplementary Figure 6k

Boxes indicate regions shown in figure. Each image is the same membrane stained with the indicated antibody.

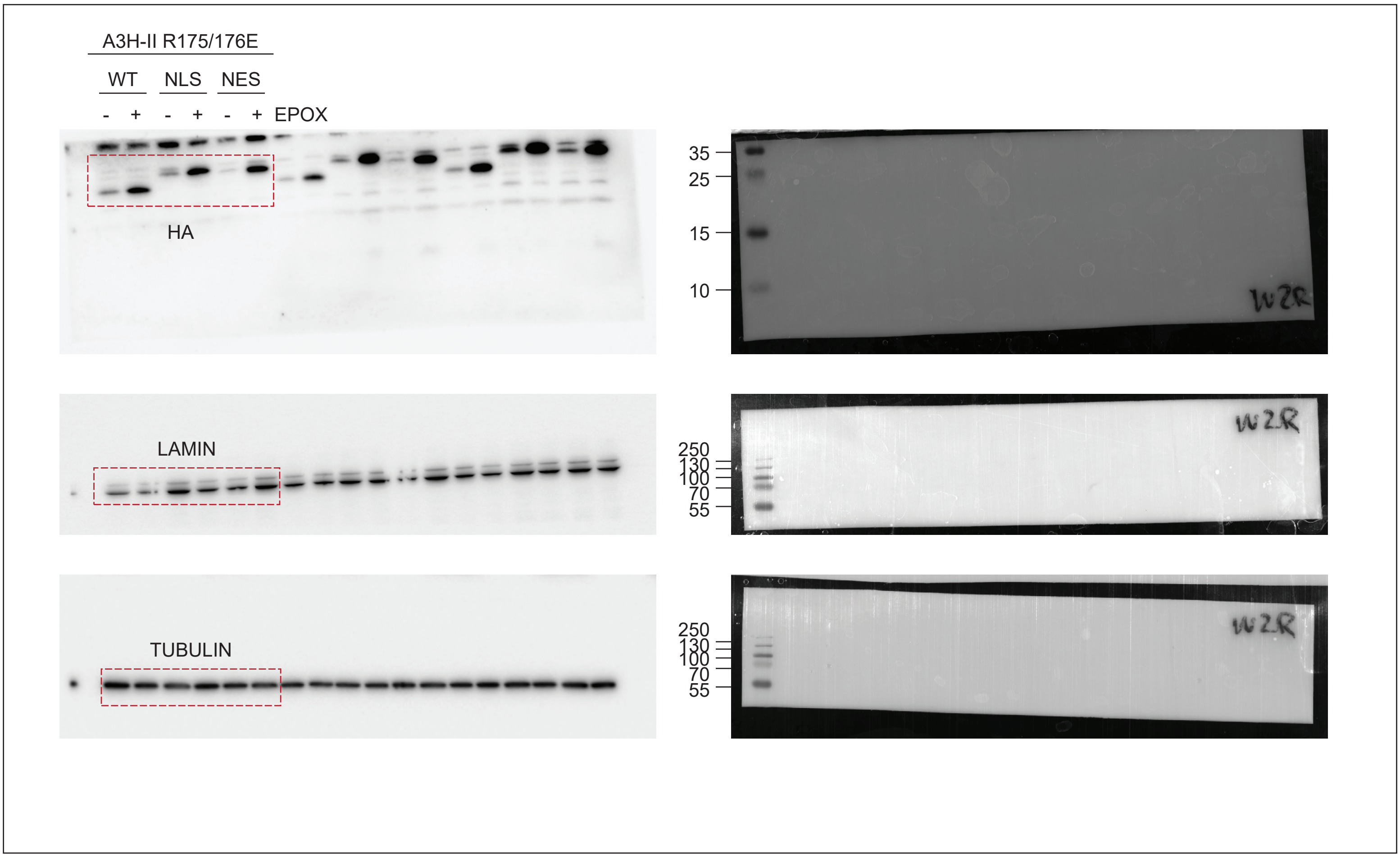

Supplement: Supplementary file 7 — Source data [file 41467_2026_68420_MOESM7_ESM.zip › Source data WB/Supplementary Figure 6/Supplementary Figure 6.pdf]
