# Supplementary material for: Guardian ubiquitin E3 ligases target cancer-associated APOBEC3 deaminases for degradation to promote human genome integrity
Source: Nat Commun. 2026 Jan 19;17:1723. doi: 10.1038/s41467-026-68420-5 (PMC12913773; doi:10.1038/s41467-026-68420-5)

Extended Data Supplementary Figure. 7b

Boxes indicate regions shown in figure. Coomassie stained SDS pages (10xHis-MBP-A3H-I, 10xHis-MBP-A3H-II, 10xHis-MBP-A3H-II-RBM).

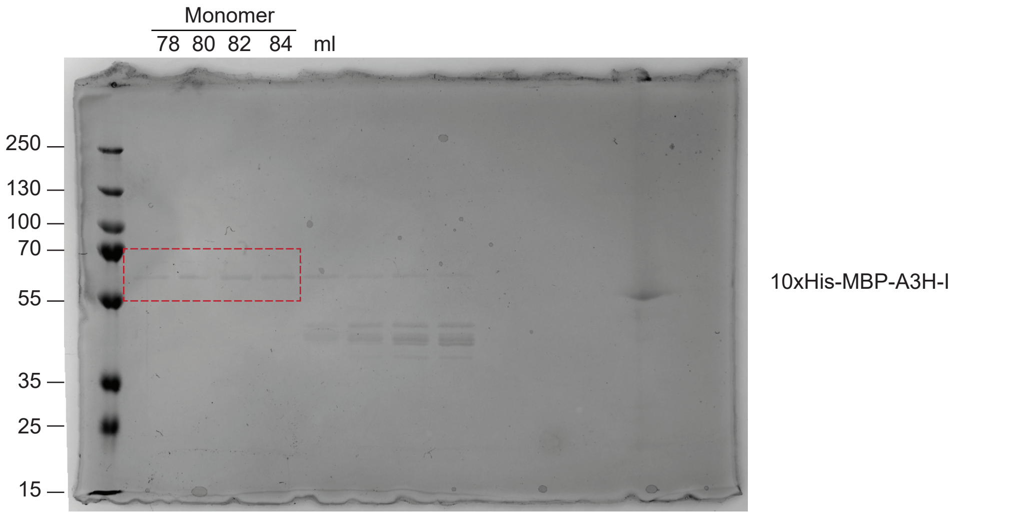

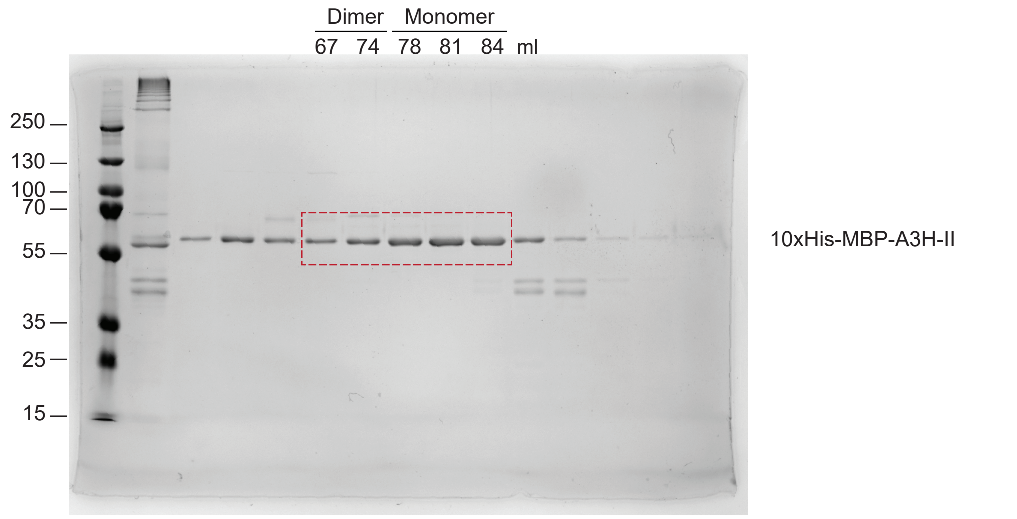

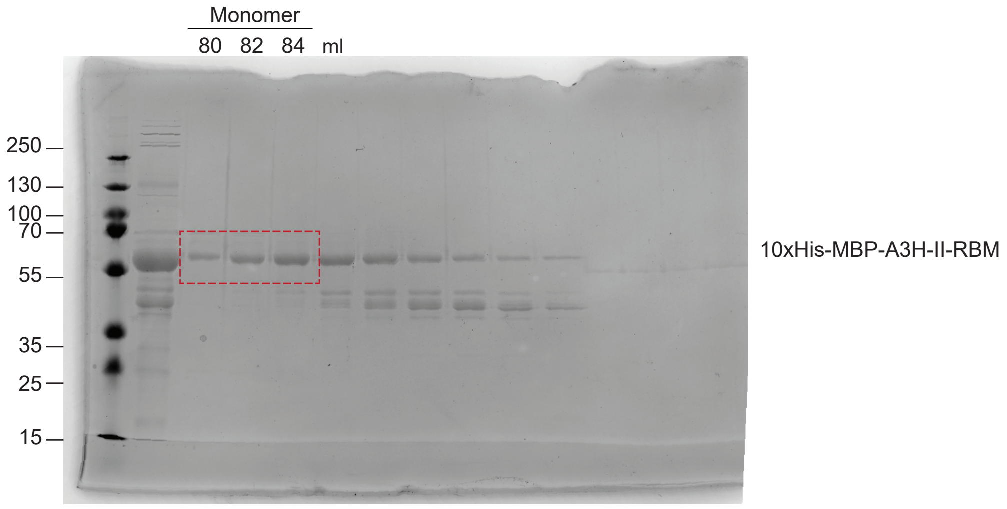

Extended Data Supplementary Figure 7c

Boxes indicate regions shown in figure. Gels were transfered onto a membrane and stained with the indicated antibody.

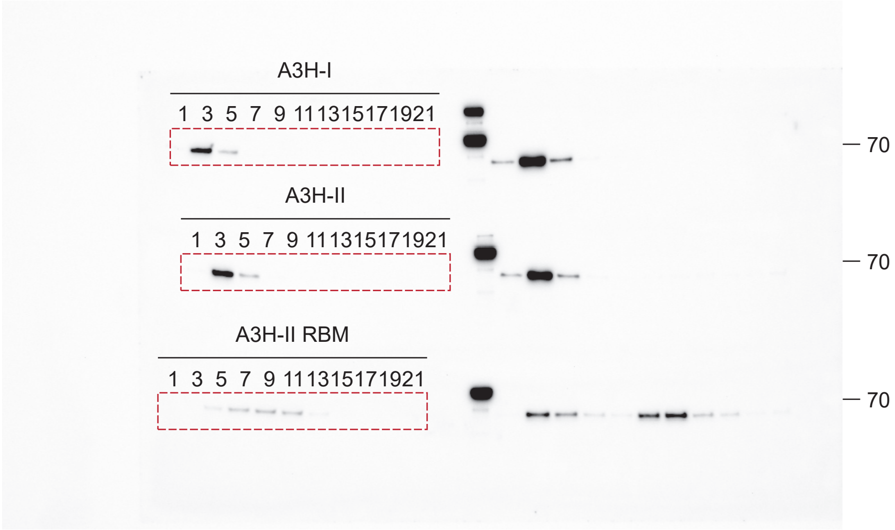

a-HIS

Extended Data Supplementary Figure 7d

Boxes indicate regions shown in figure. Gels were imaged for Trp fluorescence.

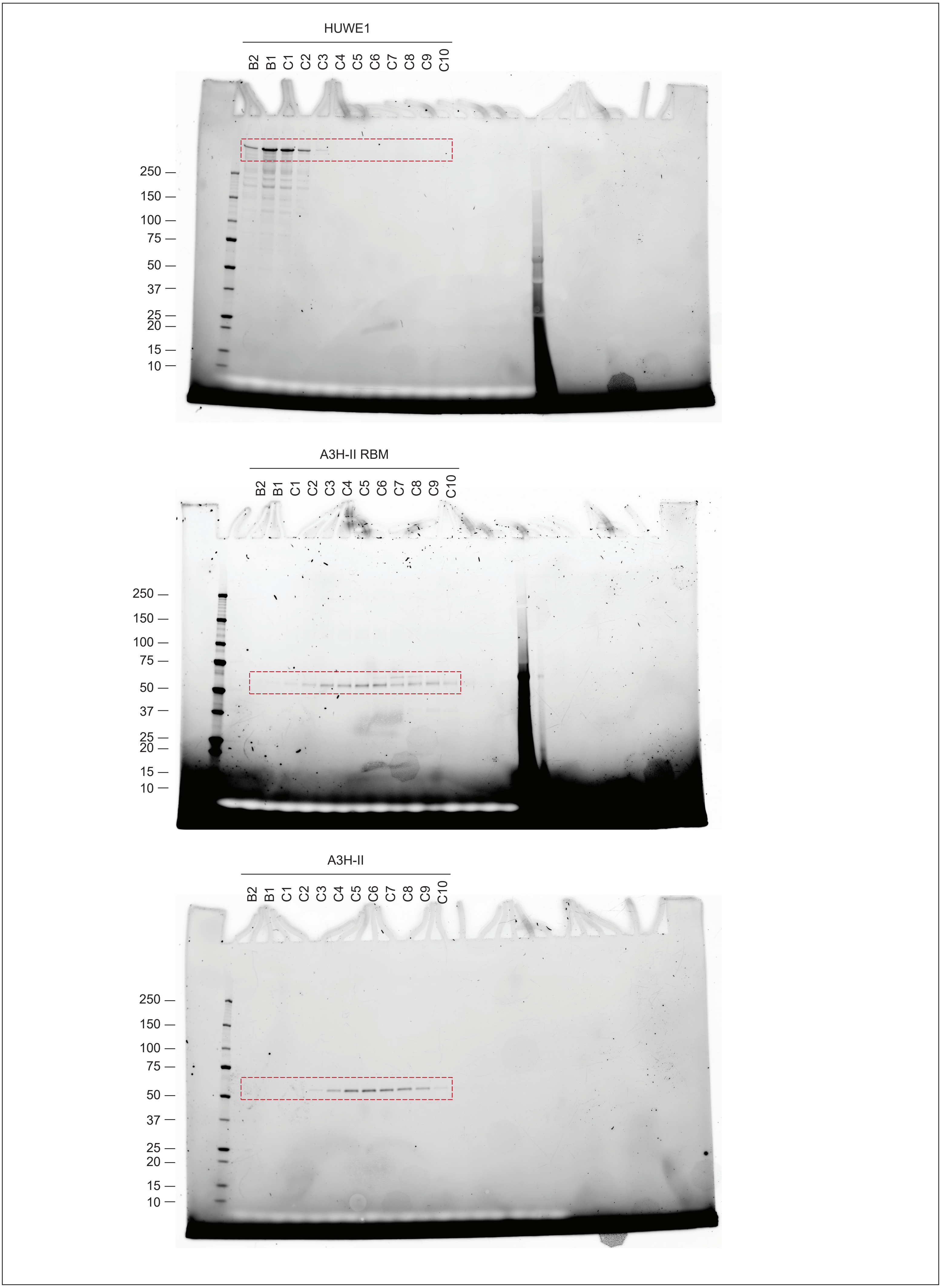

Extended Data Supplementary Figure 7e

Boxes indicate regions shown in figure. Coomassie stained SDS page (10xHis-MBP-A3A).

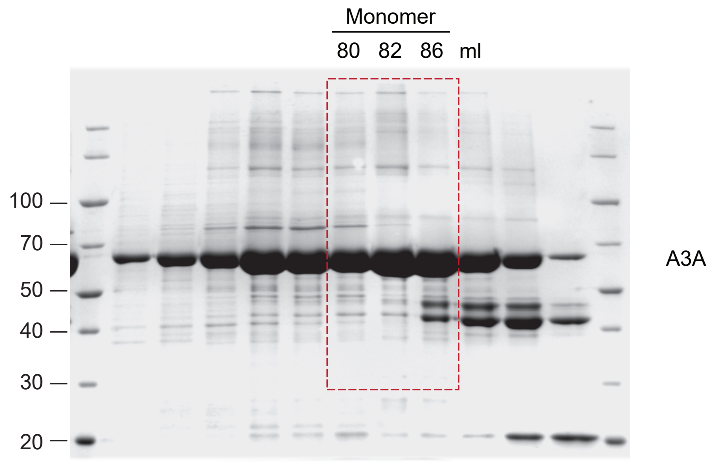

Extended Data Supplementary Figure 7f

Boxes indicate regions shown in figure. Coomassie stained SDS pages (10xHis-MBP-A3B CD1, 10xHis-MBP-A3B CD2).

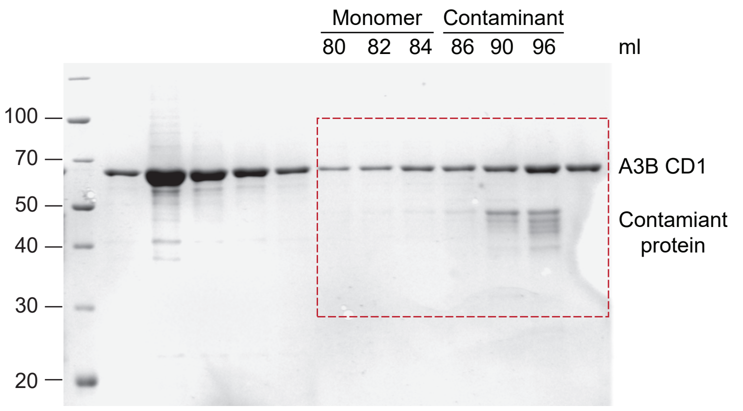

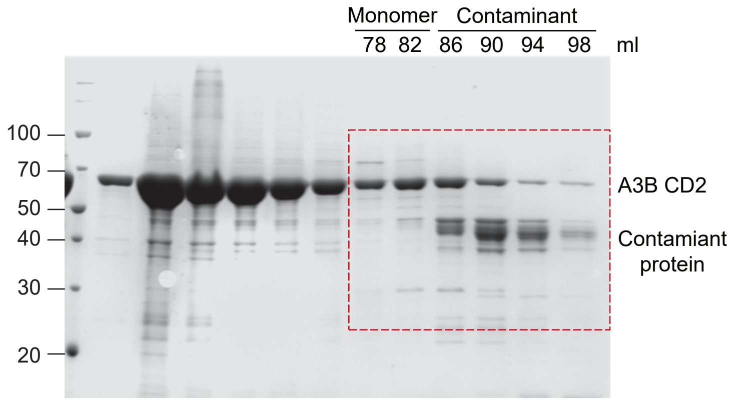

Supplement: Supplementary file 7 — Source data [file 41467_2026_68420_MOESM7_ESM.zip › Source data WB/Supplementary Figure 7/Supplementary Figure 7.pdf]
