# Supplementary material for: Guardian ubiquitin E3 ligases target cancer-associated APOBEC3 deaminases for degradation to promote human genome integrity
Source: Nat Commun. 2026 Jan 19;17:1723. doi: 10.1038/s41467-026-68420-5 (PMC12913773; doi:10.1038/s41467-026-68420-5)
Supplement: Supplementary file 7 — Source data [file 41467_2026_68420_MOESM7_ESM.zip › Source data WB/Supplementary Figure 8/Supplementary Figure 8.pdf]

Extended Data Supplementary Figure 8a

Boxes indicate regions shown in figure. Same gel was imaged for in-gel fluorescence for dighlight-488 and Trp fluorescence.

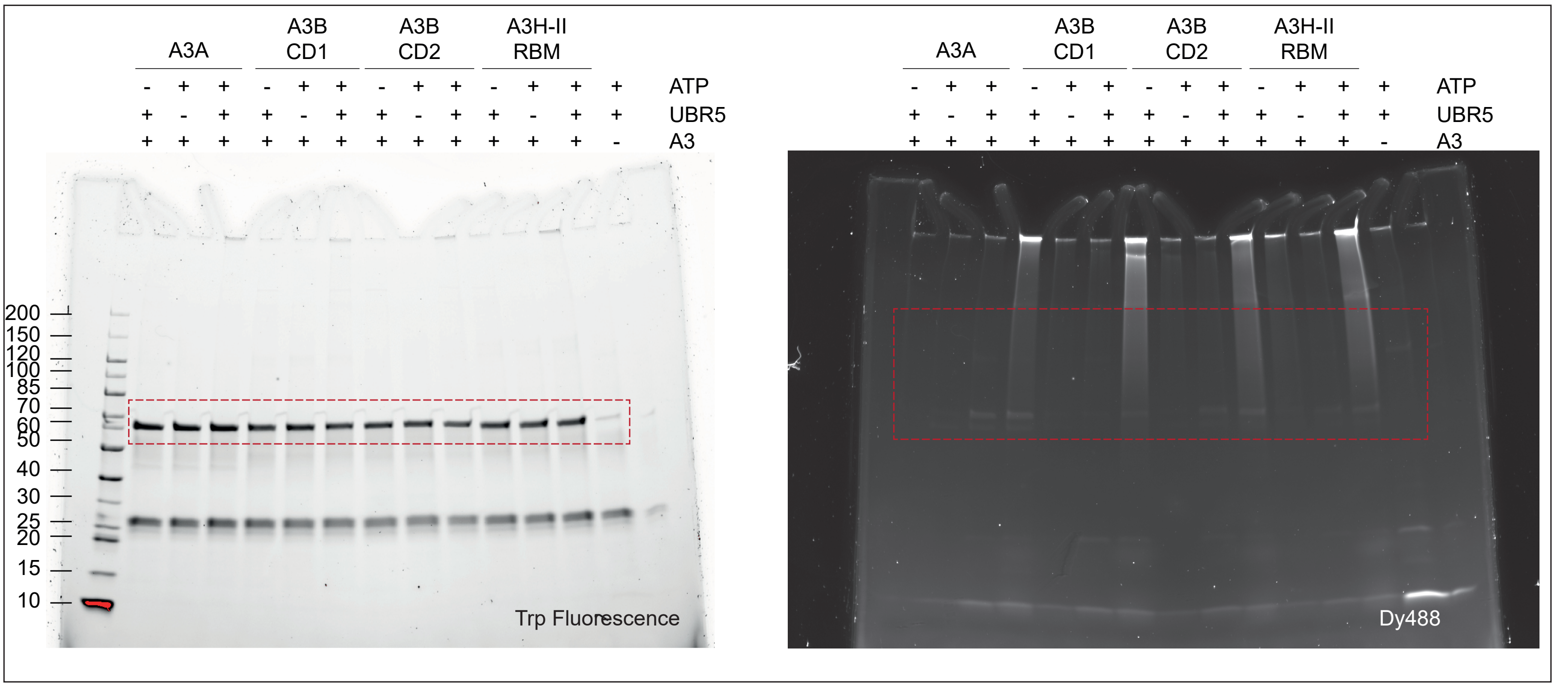

Extended Data Supplementary Figure 8c

Boxes indicate regions shown in figure. Same gel was imaged for in-gel fluorescence for dighlight-488 and Trp fluorescence.

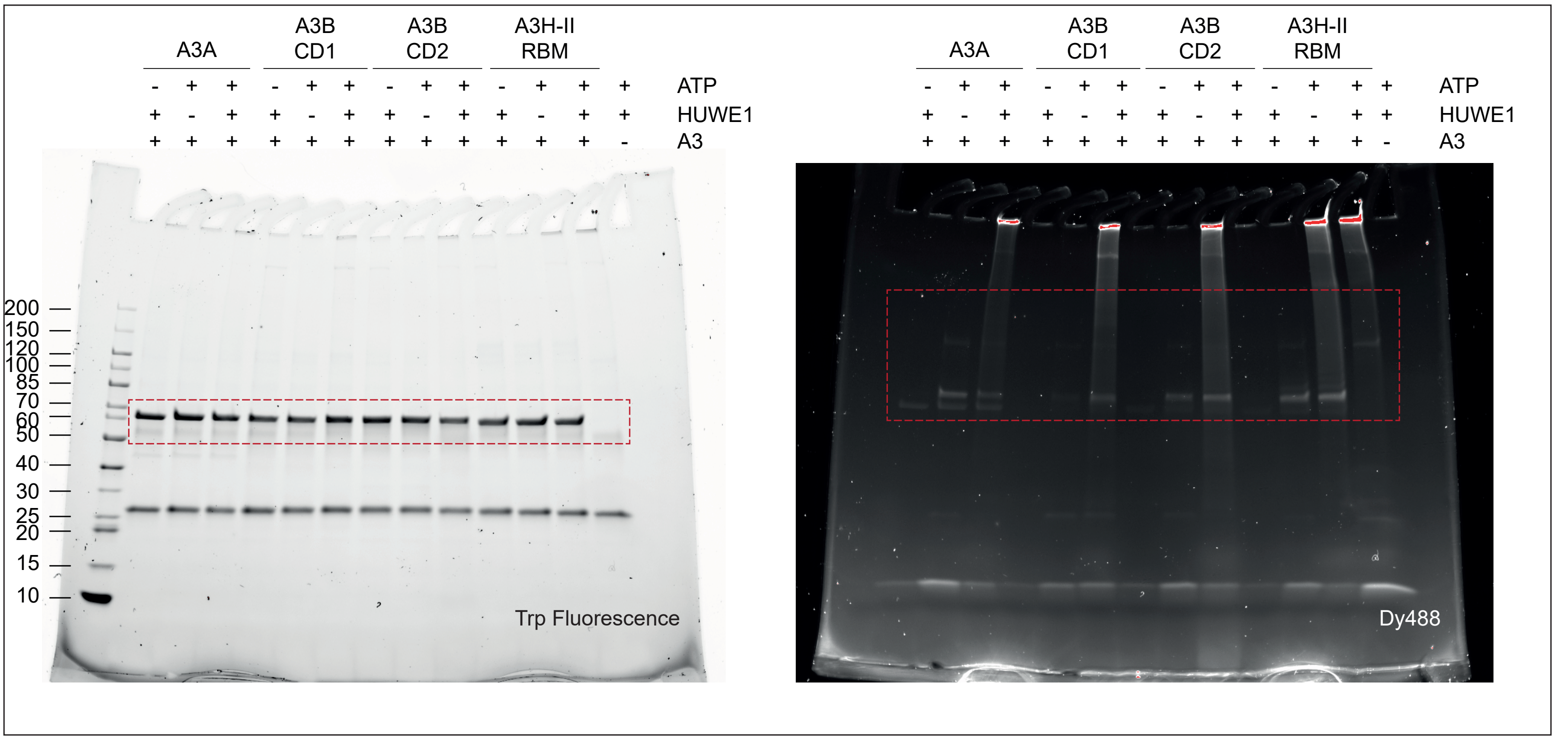

Extended Data Supplementary Figure 8e

Boxes indicate regions shown in figure. Same gel was imaged for in-gel fluorescence for dighlight-488 and Trp fluorescence.

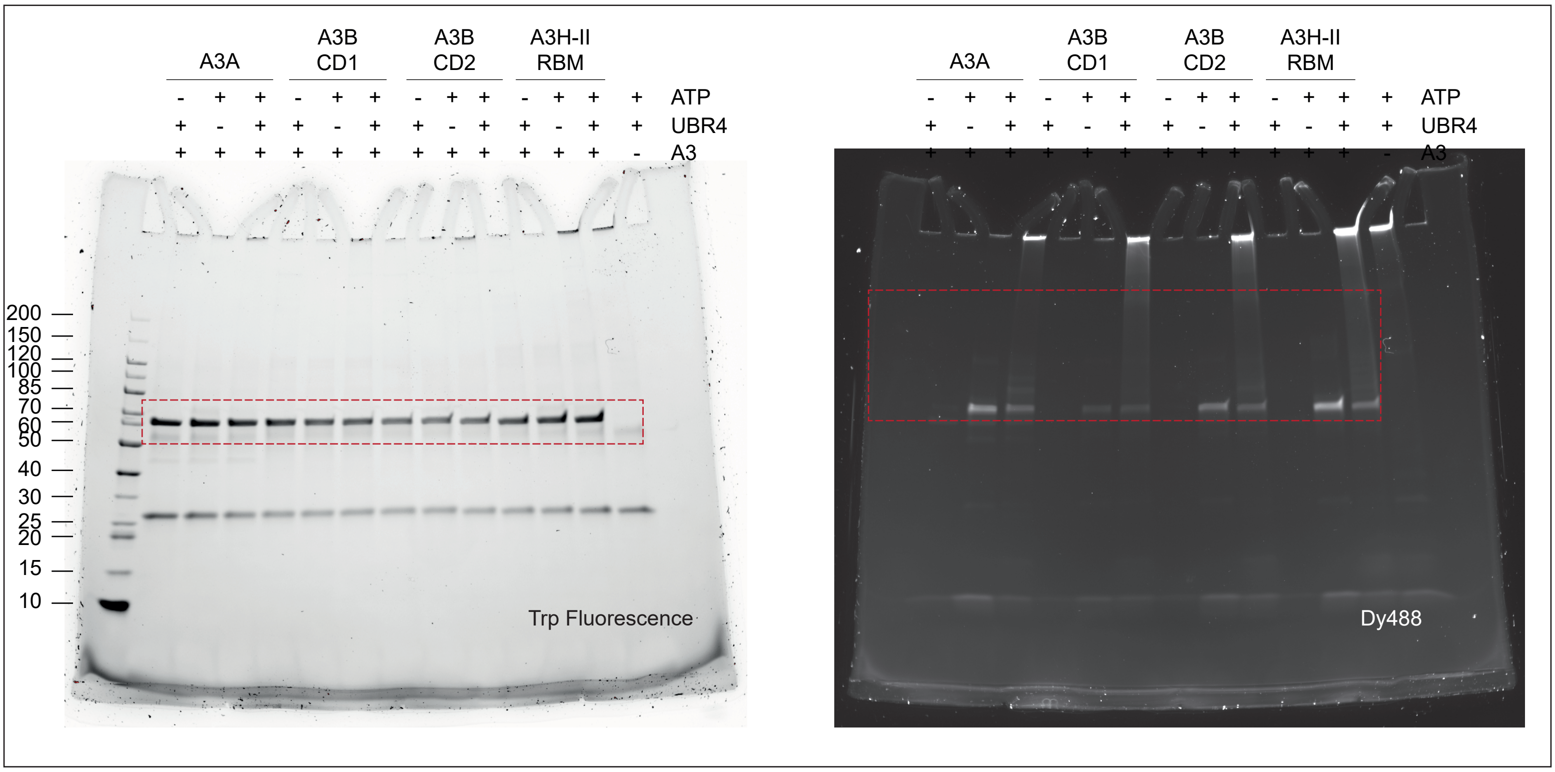

Extended Data Supplementary Figure. 9a
Boxes indicate regions shown in figure. Each image is the same membrane stained with the indicated antibody.

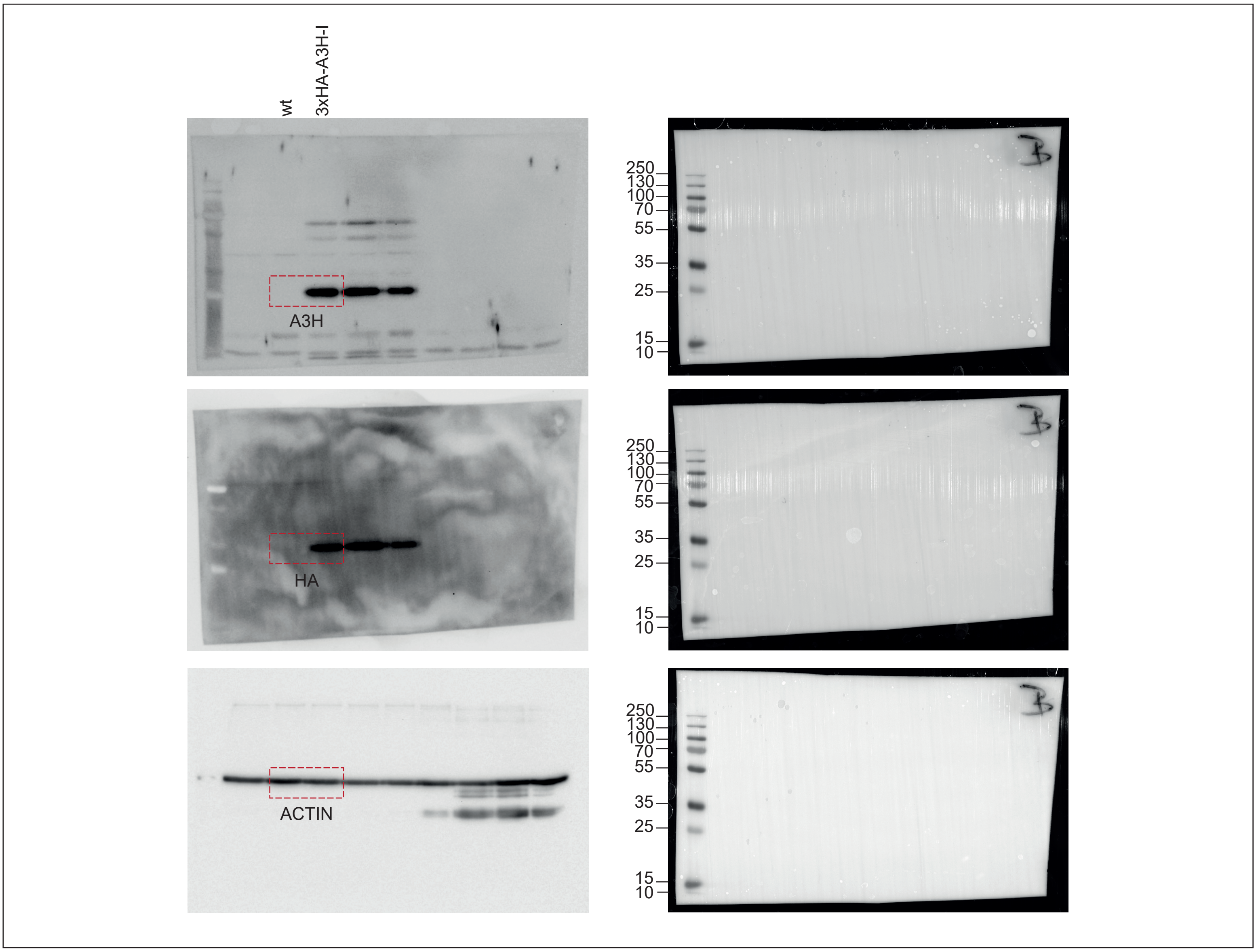

Extended Data Supplementary Figure 8g
Boxes indicate regions shown in figure. Gels were imaged for in-gel fluorescence and subsequently transfered onto a membrane and stained with the indicated antibody.

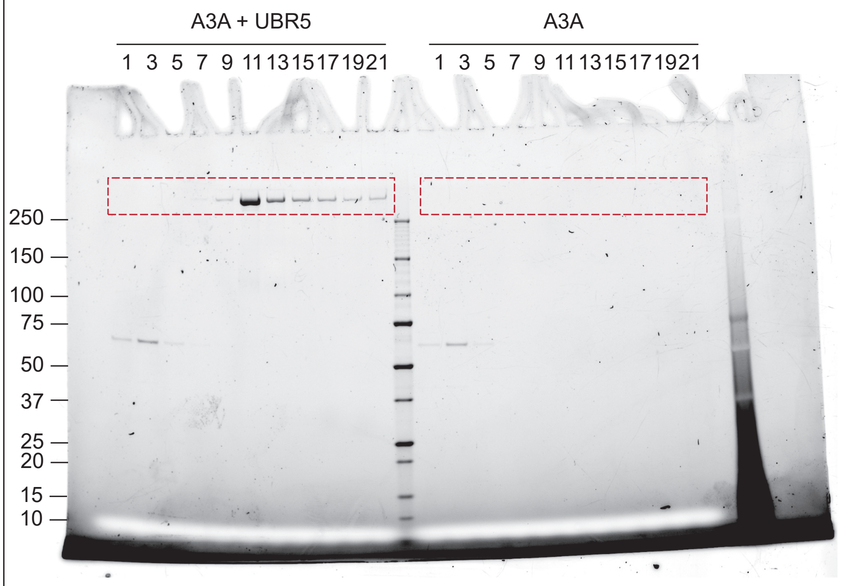

Trp Fluorescence

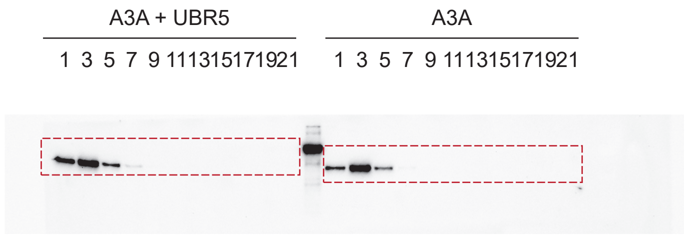

a-HIS

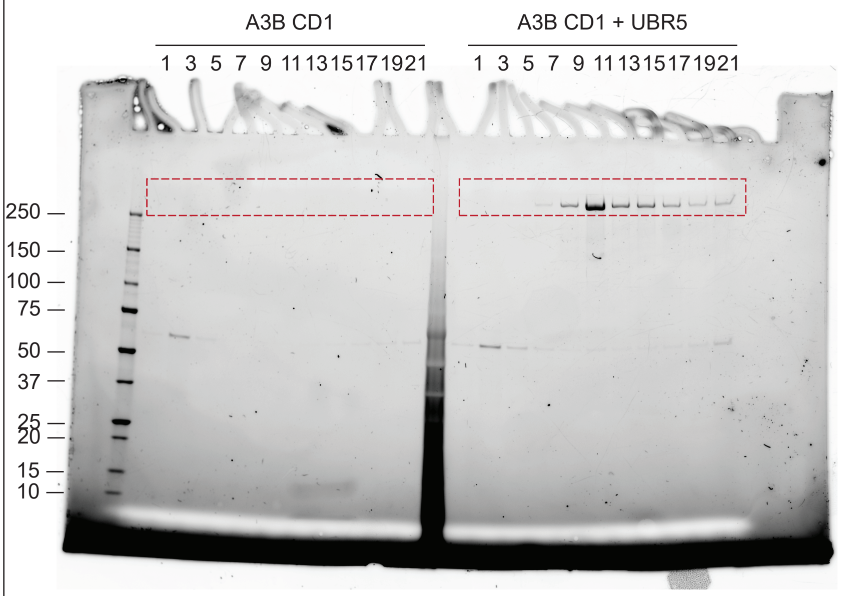

Trp Fluorescence

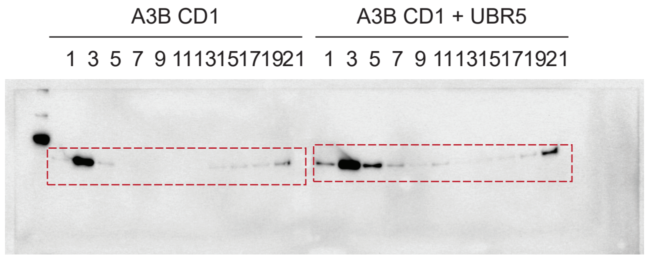

a-HIS

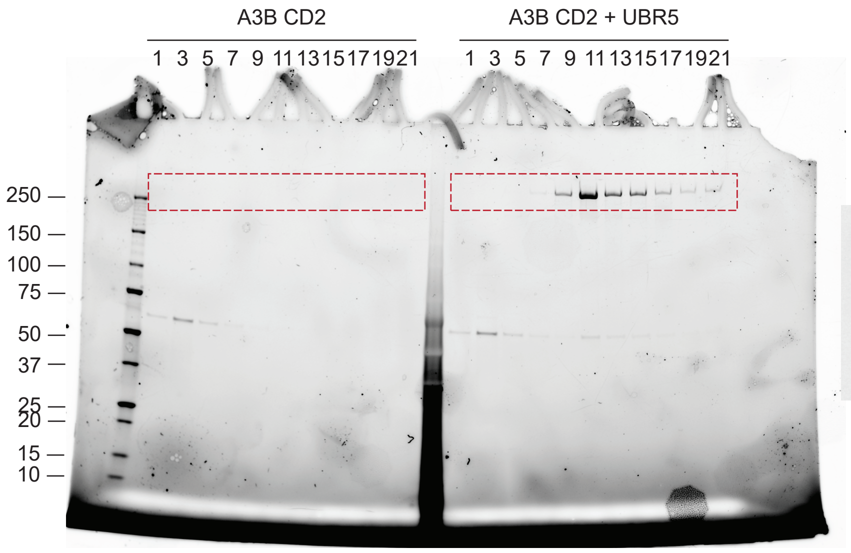

Trp Fluorescence

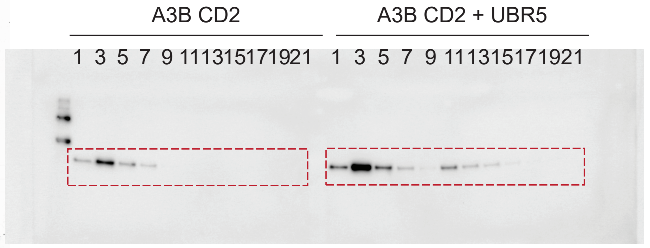

a-HIS
